# Chromosomal Passports Provide New Insights into Diffusion of Emmer Wheat

**DOI:** 10.1371/journal.pone.0128556

**Published:** 2015-05-29

**Authors:** Ekaterina D. Badaeva, Jens Keilwagen, Helmut Knüpffer, Louise Waßermann, Olga S. Dedkova, Olga P. Mitrofanova, Olga N. Kovaleva, Olga A. Liapunova, Vitaly A. Pukhalskiy, Hakan Özkan, Andreas Graner, George Willcox, Benjamin Kilian

**Affiliations:** 1 N.I. Vavilov Institute of General Genetics, Russian Academy of Sciences, Gubkina street 3, Moscow 119991, Russia; 2 Engelhardt Institute of Molecular Biology, Russian Academy of Sciences, Vavilova street 32, Moscow 119991, Russia; 3 Julius Kühn-Institut (JKI), Federal Research Centre for Cultivated Plants, Erwin-Baur-Straße 27, 06484 Quedlinburg, Germany; 4 Leibniz Institute of Plant Genetics and Crop Plant Research (IPK) Gatersleben, Corrensstrasse 3, D-06466 Stadt Seeland OT Gatersleben, Germany; 5 N.I. Vavilov Institute of Plant Industry (VIR), Russian Academy of Agricultural Sciences, Bolshaya Morskaya street 44, St. Petersburg 190000, Russia; 6 University of Cukurova, Faculty of Agriculture, Department of Field Crops. 01330 Adana, Turkey; 7 Archéorient CNRS UMR 5133, Université de Lyon II, Antenne d’Archéorient, Jalés, Berrias, F-07460 St-Paul-le-Jeune, France; Università Politecnica delle Marche, ITALY

## Abstract

Emmer wheat, *Triticum dicoccon* schrank (syn. *T*. *dicoccum* (schrank) schÜbl.), is one of the earliest domesticated crops, harboring a wide range of genetic diversity and agronomically valuable traits. The crop, however, is currently largely neglected. We provide a wealth of karyotypic information from a comprehensive collection of emmer wheat and related taxa. In addition to C-banding polymorphisms, we identified 43 variants of chromosomal rearrangements in T. dicoccon; among them 26 (60.4%) were novel. The T7A:5B translocation was most abundant in Western Europe and the Mediterranean. The plant genetic resources investigated here might become important in the future for wheat improvement. Based on cluster analysis four major karyotypic groups were discriminated within the *T*. *dicoccon* genepool, each harboring characteristic C-banding patterns and translocation spectra: the balkan, asian, european and ethiopian groups. We postulate four major diffusion routes of the crop and discuss their migration out of the Fertile Crescent considering latest archaeobotanical findings.

## Introduction

Emmer wheat, *Triticum dicoccon* Schrank (syn. *T*. *dicoccum* (Schrank) Schübl.), is one of the earliest domesticated crops. Along with einkorn wheat, barley, lentil, pea, and flax, it constituted the ‘founder crop assemblage’ on which ‘Old World’ agriculture was built [1]. *Triticum dicoccon* was domesticated in the Fertile Crescent from its wild progenitor *T*. *dicoccoides* (Körn. ex Asch. et Graebn.) Körn. ex Schweinf. between 12,000–10,000 years ago [2–5].

Emmer wheat was a staple crop of Neolithic agriculture and it was widely cultivated for over 7,000 years, until the second half of the third millennium BP, when it began to be replaced by higher-yielding and free-threshing bread wheat (*T*. *aestivum* L.) and durum wheat (*T*. *durum* Desf.) [1,2,6]. Several studies suggested that the initial domestication of emmer occurred independently at several sites in the Fertile Crescent [3,7,8]. Favorable alleles, which enabled adaptation to human-made habitats and husbandry as well as to new climate conditions, were selected under cultivation [1,9,10]. Hybridization events between domesticated emmer and its wild progenitor, as well as selection and enrichment of naturally occurring mutations provided a relatively wide genetic basis as the crop evolved [2]. Tetraploid domestic emmer wheat (genomic formula BBAA), which already harbored certain genome plasticity [11], was able to tolerate a wide range of biotic and abiotic stresses and to thrive under cultivation, well outside the Fertile Crescent.

Today *T*. *dicoccon* is a neglected crop, cultivated mainly in marginal areas of Ethiopia, Yemen, Morocco, Turkey, Iran, Transcaucasia, India, and the Balkans, often in mixed stands with other wheats [12–18]. Emmer was probably not affected by a severe genetic bottleneck of modern breeding strategies [19,20], as in the case of more intensely bred crops [21–23]. Therefore emmer wheat could provide a rich source of genetic variation, valuable agronomical traits and ecological plasticity [15,17,24]. Almost 4,800 emmer accessions are maintained in 52 *ex situ* genebank repositories all over the world [25], which could be utilized for wheat improvement.

Emmer wheat has been subdivided into four morphologically and ecologically distinct subspecies: (1) subsp. *maroccanum* Flaksb. (Moroccan emmer); (2) subsp. *abyssinicum* Vavilov (Ethiopian emmer); (3) subsp. *dicoccon* (= subsp. *europaeum* Vavilov; European emmer), including convar. *dicoccon* (European emmer) and convar. *serbicum* (Volgo-Balkan emmer), and (4) subsp. *asiaticum* (Eastern emmer) [13,26]. Other wheat classification systems (e.g. [27]) do not consider them as separate taxonomical units.

Molecular studies of *T*. *dicoccoides* clearly distinguished two wild emmer races or genepools, which are further subdivided: a wild western/southern race comprised materials sampled in Israel, Jordan, Lebanon and Syria; and a wild eastern/northern race consisted of samples collected in Turkey, Iraq and Iran [3]. The authors of this publication summarized the current knowledge on the geographic distribution, population structure and domestication history of wild emmer wheat. According to their data, domesticated emmer originated from the eastern wild emmer race which can be due to, for example, (a) late ripening or (b) easier threshing due to thinner glumes & shorter and weaker awns that confer them the adaptive advantages for cultivation.

Geographical expansion of domestic emmer was intimately associated with human migrations [28]. It was a long and complex process in which wheat genotypes became adapted to new habitats and climates. The genetic structure of local wild and domesticated emmer populations was affected, among other factors, by exchange of seed stock during migration and by gene flow between wild and domesticated wheats or between different locally adapted domestic emmer populations [10,19,29–31].

Beside molecular markers, genetic diversity of wheat species or populations can be assessed using cytogenetic approaches. The C-banding technique proved to be powerful in phylogenetic studies of cereal crops [32]. This is because the C-banding patterns are chromosome- and cultivar-specific and independent of environmental cues [33]. C-banding patterns are highly polymorphic, although the sense of this phenomenon is not known. High diversity of the C-banding patterns creates the uniqueness of karyotypes of each form. Despite this, C-banding patterns were never considered in population analysis due to impossibility of direct application of chromosomal images for statistical testing. This problem can be circumvented by using the recently developed approach of chromosomal passportization—a description of karyotypes in a digital form by comparing all chromosomes of each line with the generalized idiogram [34].

Earlier, using the C-banding technique, we observed some peculiarities of European emmer, in particular the abundance of the 5B:7A translocation [35,36]. Our previous studies however did not cover the entire geographic range of emmer cultivation. None of the accessions studied by us earlier were included in experiments by other laboratories using molecular markers [3,16,30,37,38], thus precluding a direct comparison.

The aim of this study was to perform a comprehensive cytogenetic analysis using chromosomal passports based on the C-banding patterns of karyotypes in a broad collection of *T*. *dicoccon* lines. We also aimed to include wild emmer wheats and other tetraploid wheat taxa in order to unravel phylogenetic relationships among domestic emmer populations and to obtain new insights into emmer domestication history by suggesting possible diffusion routes of the crop.

## Materials and Methods

### Plant material

In this study, we followed the wheat classification system of [13]. For the spelling “*dicoccon*”, see [39,40].

In total, karyotypes of 486 accessions of four tetraploid hulled wheat taxa and one free-threshing taxon were analyzed using the C-banding technique (S1 Table). Of them, 431 accessions were cytogenetically stable (thus consisting of one genotype only), whereas 36 accessions segregated into 2, eleven—into 3, six into 4, and two into 5 distinct genotypes that differed from one another in the C-banding patterns and/or in the presence of chromosomal rearrangements. All genetically distinct genotypes were treated as separate entities and designated as “lines”.

Thus, altogether we considered 570 lines. Among them we investigated 446 lines of *T*. *dicoccon* collected from 47 countries, 105 lines of *T*. *dicoccoides* from seven countries, covering its whole present-day natural distribution range, 10 lines of *T*. *durum*, 5 lines of *T*. *karamyschevii* Nevski ( *T*. *georgicum* Dekapr. et Menabde or *T*. *paleocolchicum* Menabde,) found in Georgia, and 4 lines of *T*. *ispahanicum* Heslot, originally collected near Isfahan in Iran [41]. Most botanists treat them as intraspecific taxa of domesticated emmer (i.e. *T*. *dicoccon* var. *georgicum* Dekapr. et Menabde and subsp. *ispahanicum* (Heslot) Dorof.) [27]. All samples came from *ex situ* genebank repositories (S1 Table).

### C-banding

Chromosomal preparations and C-banding analysis were carried out according to [42]. The slides were analyzed on a Zeiss Imager D1 microscope, and selected metaphase plates were photographed at 100× magnification with a black-and-white AxioCam HRm digital camera using the AxioVision software package, release 4.6. Chromosomes were classified according to the standard genetic nomenclature, and “chromosomal passports” were constructed for 545 lines as described in [34,43] for subsequent statistical analyses (25 lines were not considered because they were either taxonomically misclassified, or their authenticity was doubted, see S1 File for more details).

**Fig 1:**
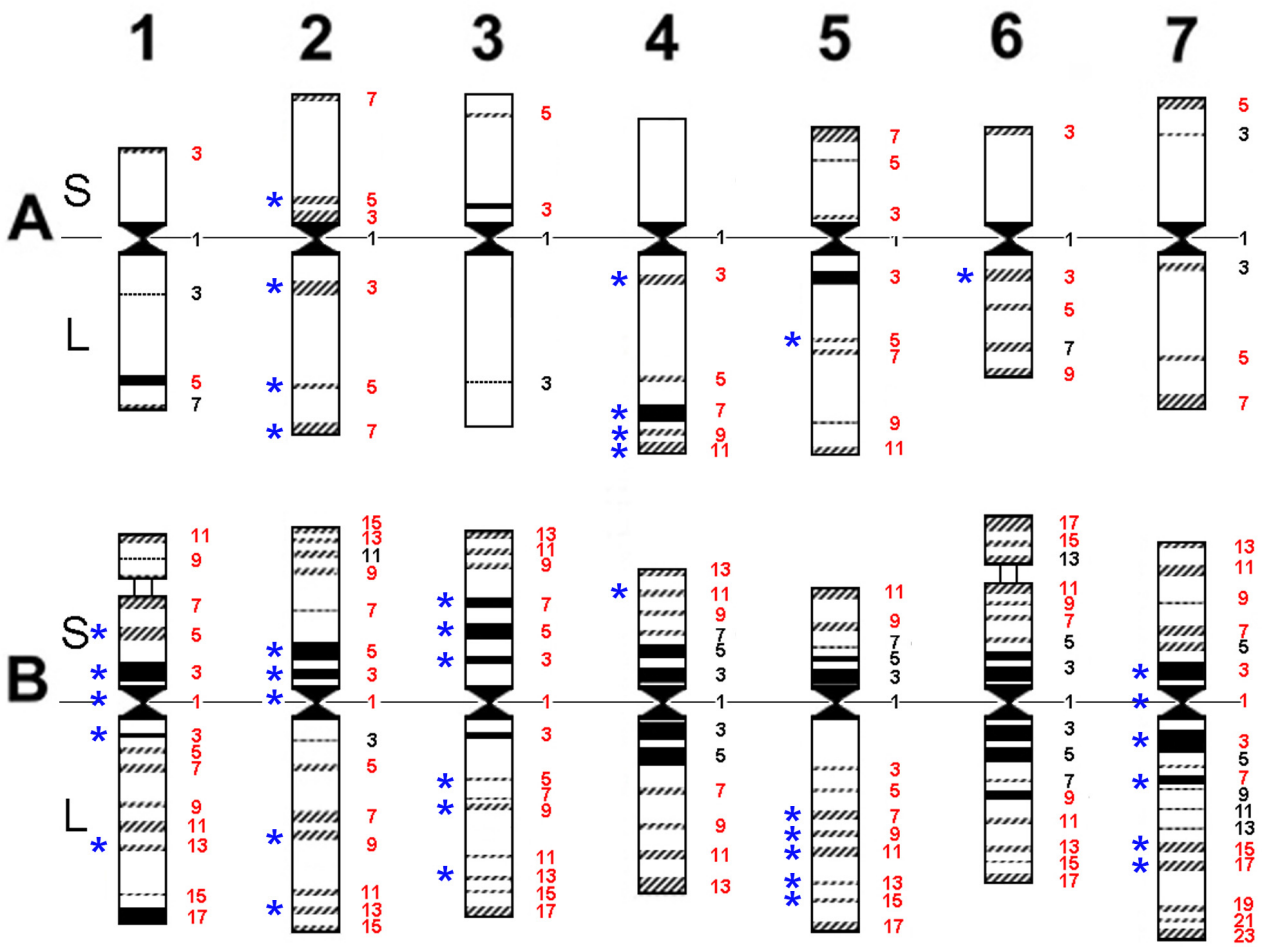
Generalized idiogram of wheat A and B genome chromosomes. A and B—wheat genomes; 1–7—homoeologous groups; S and L—short and long arm of a chromosome, respectively. Large permanent C-bands are shown as solid blocks; polymorphic C-bands are shown as shaded blocks; small inconsistent C-bands are indicated by dashed lines. Positions of C-bands are numbered at the right-hand side of each chromosome. The 112 C-bands considered in “chromosomal passports” are shown in red; 38 C-bands most essential for discrimination of karyotypic groups are indicated with *.

Briefly, chromosomal passports were constructed as follows. Each chromosome of a particular line was compared with a generalized idiogram of wheat chromosomes (Fig 1). An idiogram indicates all possible positions, in which Giemsa C-bands can be detected on a chromosome. Each position was numbered by an odd numeral from 1 (centromere) to 3–23 depending on the chromosome. Each band can be described by a simple formula that includes chromosome designations (1A–7B), chromosome arm (short, S or long, L) and band position on the arm. For example, the perinucleolar C-band on the chromosome 1B was defined as 1BS7 (Fig 1).

Among 147 positions of C-bands revealed for all lines we investigated, several bands were excluded from the analysis for either of the following reasons: (I) they were monomorphic, like C-bands constituting pericentromeric heterochromatin complexes of the B-genome chromosomes; or (II) bands were too small or inconsistent to score.

**Fig 2:**
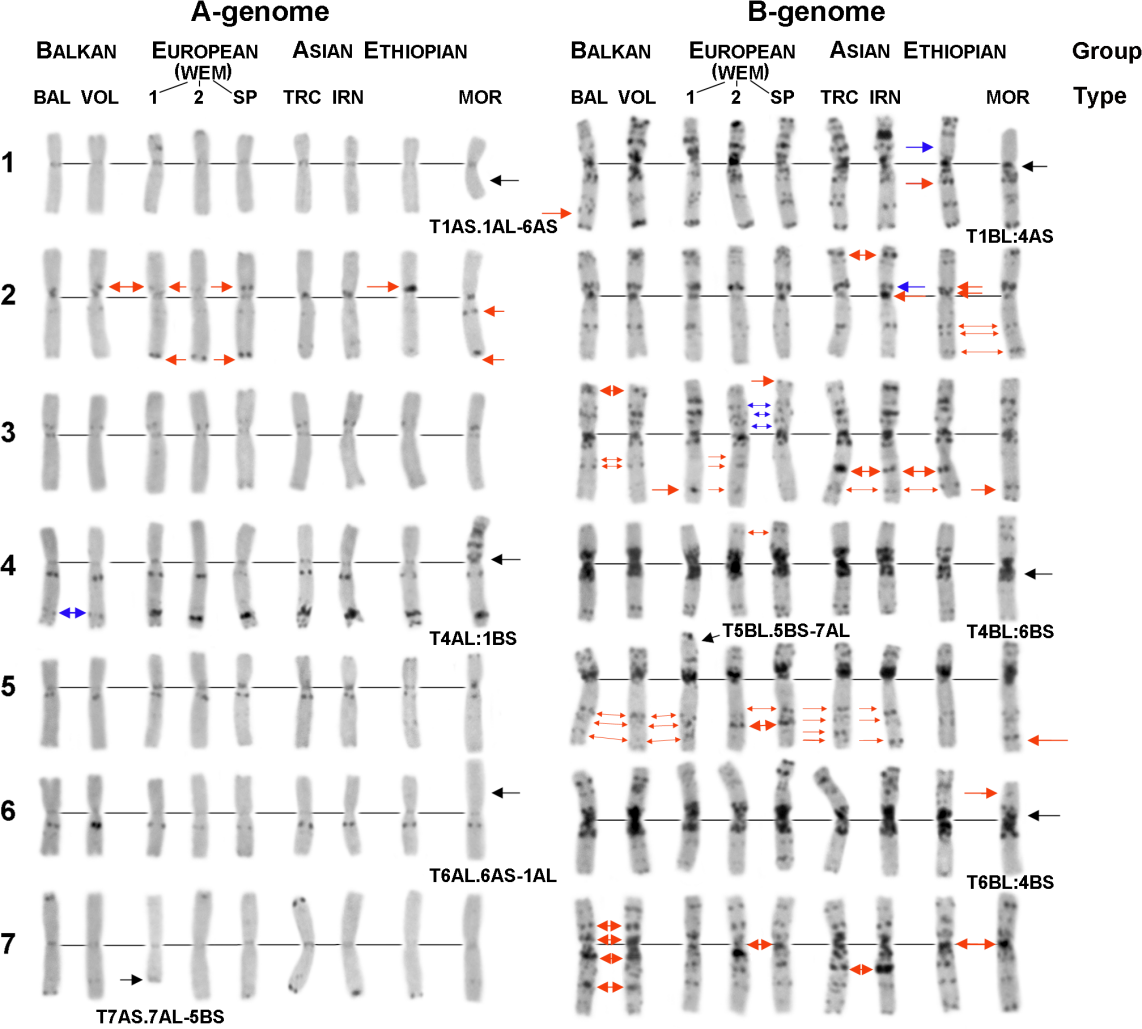
Main karyotypic groups/types distinguished among *T*. *dicoccon* accessions on the basis of visual analysis of C-banding patterns. Left side—A-genome chromosomes, right side—B-genome chromosomes; 1–7—homoeologous groups. Chromosomal groups: Balkan, European, Asian, Ethiopian (PI 94664, Saudi Arabia); chromosomal types: Bal (PI 434996, Montenegro), Vol (k-30728, Nizhny Novgorod); WEM-1 type prevailing in European-Mediterranean emmer lines with 7A:5B translocation (k-1730, Germany); WEM-2 (PI 352332, Belgium); WEM-Sp (PI 275998, Spain); Trc (k-14937, Georgia); Irn (TA10540, Iran), Mor (k-22246, Morocco). Small red arrows indicate positions of small group-specific bands; large red arrows show the positions of C-bands with an enlarged size or group-specific supernumerary C-bands; blue arrows indicate group-specific bands with a decreased size (small arrow) or the positions of bands that are missing in the group-specific chromosome variant (large arrow). Large black arrows show the breakpoint position in region-specific translocation variants: T7AS.7AL-5BS and T5BL.5BS-7AL—rearranged chromosomes formed as a result of the T7A:5B translocation; T4AS:1BL and T1BS:4AL—rearranged chromosomes formed as a result of the T4A:1B translocation; T1AS.1AL-6AS and T6AL.6AS-1AL—rearranged chromosomes formed as a result of the T1A:6A translocation; T4BS:6BL and T6BS:4BL—rearranged chromosomes formed as a result of the T4B:6B-1 translocation (see S3 and S4 Tables).

The size of all 112 informative bands (Fig 1, red numbers) was estimated as follows: “0”—absent; “1”—small, “2”—medium, and “3” large or very large (for example, band 1BS7 on Chr 1B of the Bal (= 1), Trc (= 2), and Irn (= 3) emmer, respectively (Figs 1 and 2)). Owing to the frequent occurrence of pericentric inversions on chromosome 4B, this parameter was considered in the chromosomal passports as an additional variance. It was estimated as the position of the centromere within four blocks of the centromeric heterochromatin complex: “0”—all four blocks are located on the long arm (inv4B); “1”—one block is located on the short and three on the long arm; “2”—two blocks are located on the long and two on the short arm; “3”—three or all four blocks are located on the short arm.

All these numbers were entered into an Excel spread sheet containing a matrix of 113 columns corresponding to the number of informative characters plus one column with the accession code, and 545 rows, each row corresponding to one line (S2 Table). This dataset was then used to infer the genetic clustering.

### Network Reconstruction and Genetic Clustering

First, we used the chromosomal passport data of *T*. *dicoccon* lines for estimating the number of main clusters in the collection. Based on this data, we utilized k-medoids using Hamming distance and compute the gap statistic [44,45]. For determining the number of main clusters, we utilize the criteria defined by Tibshirani *et al*. [44], but asked for twice standard error to be more strict. Shepard plot as well as R^2^ values indicated that it will be reasonable to use nmds statistics.

NMDS plot and Neighbor-joining tree were computed based on Hamming distance between all 545 lines using R project [45]. Accessions were colored according to the main cluster components of *T*. *dicoccon* lines determined by k-medoids, and one additional color (black) for other taxa. In the Neighbor-joining tree, we colored an edge with the unique color of all leaves in this subtree and otherwise grey (S12 Fig).

### Karyotype distribution map

Point localities were geo-referenced using the global gazetteer version 2.1 (www.fallingrain.com/world/) (S1 Table).

## Results

### Chromosomal polymorphisms of domesticated emmer

Karyotypes of domesticated emmer showed high diversity of C-banding patterns. B-genome chromosomes were more polymorphic than A-genome chromosomes. The lowest diversity of C-banding patterns was found for chromosome 3A (3 polymorphic variants), while chromosomes 2A and 4A proved to be most variable among the A-genome chromosomes (37 and 38 variants, respectively). On the B-genome, the lowest polymorphism was observed for chromosome 4B (25 variants) and the highest—for chromosomes 3B and 7B (78 and 53 variants, respectively) (S1–S6 Figs).

The comparison of emmer lines from different geographical regions enabled the detection of “region-specific” C-banding patterns (and thus karyotypes) (Fig 2). Visual karyotype comparison revealed that this regional specificity was mainly associated with variation out of 38 of 112 C-bands scored (Fig 1, indicated with *). Polymorphism of the remaining bands determined the uniqueness of karyotype of individual lines.

Emmer wheats from the Balkans provide a first good example for genotypes with “region-specific” banding patterns (Fig 2 and S1*A–*S1*D* Fig). Unique banding patterns were observed on Chr 4A, 1B, and 7B, which either contained additional C-bands (e.g. 1BL13), or C-bands with reduced (4AL7) or increased (7BS3, 7BL3, 7BL15) sizes (Figs 1 and 2). Based on high chromosome similarity among emmer from the Balkans, as well as based on “region-specific” polymorphic bands, these lines were assigned to the Balkan karyotypic group and designated as Bal chromosomal type. Similar peculiarities of C-banding patterns on Chr 4A, 2B, 3B, and 7B were observed in many emmer lines from the Volga region (Fig 2 and S2*A*, S2*F*, S2*J*, S2*K*, S2*M*, S2*N and* S2*Q* Fig). Therefore, these lines can be attributed to the Balkan group. However, none of them carried the third, additional C-band 1BL13 and also the C-banding patterns of Chr 2A, 6A, 7A, 5B were different (Fig 2 and S2 Fig). Based on these observations, we designated these lines as chromosomal Vol type.

A second group, consisting of *T*. *dicoccon* lines collected in Western Europe and the Mediterranean (WEM) was most diverse. However, all these lines showed similar C-banding patterns of Chr 2A, 4A, 2B and 5B (Fig 2 and S3 Fig) and were characterized by the abundance of the translocation T7A:5B (S3*E*, S3*J*, S3*K*, S3*M*, S3*N*, S3*P*, S3*Q and* S3*S* Fig). This translocation was identified (alone or in combination with secondary translocations) in 25 out of 93 genotypes (27%) from WEM (S3 Table). Three major chromosomal types were identified among the European karyotypic group: the Spanish (WEM-Sp) type prevailing in Spain (S3*A*, S3*B and* S3*D* Fig), and two more widely distributed types: WEM-1 (predominantly, but not obligatorily carrying the 7A:5B translocation; S3*E*, S3*K–*S3*N*, S3*P*, S3*Q and* S3*S* Fig) and WEM-2 (S1*K*, S1*M*, S1*S and* S1*T* Fig; S3*H–*S3*J*, S3*O*, S3*T and* S3*V* Fig; S5 *M* Fig; S6*D and* S6*K* Fig). The WEM-1, WEM-Sp and WEM-2 types can be distinguished by C-banding patterns on Chr 3B, Chr 5B and Chr 7B (Fig 2 and S3 Fig).

Lines from Transcaucasia and Iran showed region-specific banding patterns that defined the Asian group. Although karyotypically similar to the Balkan group, this group can be discriminated based on C-banding patterns of Chr 4A, 3B, 5B, and 7B (Fig 2 and S4 Fig). The Asian group can be divided into two chromosomal types: i) the Transcaucasian (Trc), and ii) the Iranian (Irn) type. The Irn type differed from the TRC type in the larger size of some interstitial and pericentromeric C-bands, especially on Chr 2B (Fig 2, indicated with red arrows). This chromosome carried a prominent pericentromeric C-band and a reduced 2BS5 band. Furthermore, chromosome 7B harbored often a significantly enlarged 7BL7 band (Fig 2 and S4*J*, S4*K and* S4*M Fig*).

Highly specific C-banding patterns, especially on Chr 2A, 1B, and 2B, defined the Ethiopian group (S5*O*, S5*P*, S5*R–*S5*S*, S5*V and* S5*X* Fig). In particular, Chr 2A was characterized by a prominent C-band 2AS5, the marker C-band 1BS5 on Chr 1B was missing, while Chr 2B contained an additional C-band 2BL3 between the pericentromeric and proximal blocks (Figs 1 and 2).

Domestic emmer from North Africa was highly heterogeneous and belonged to at least three chromosomal groups. Twelve lines were assigned to a separate group, which was defined as Mor chromosomal type. It differed from other emmer lines found in North Africa in the C-banding patterns of 2A, 2B, 4B, 5B and 7B chromosomes (Fig 2 and S5*D–*S5*J* Fig).

### Chromosomal rearrangements in domesticated emmer

**Fig 3:**
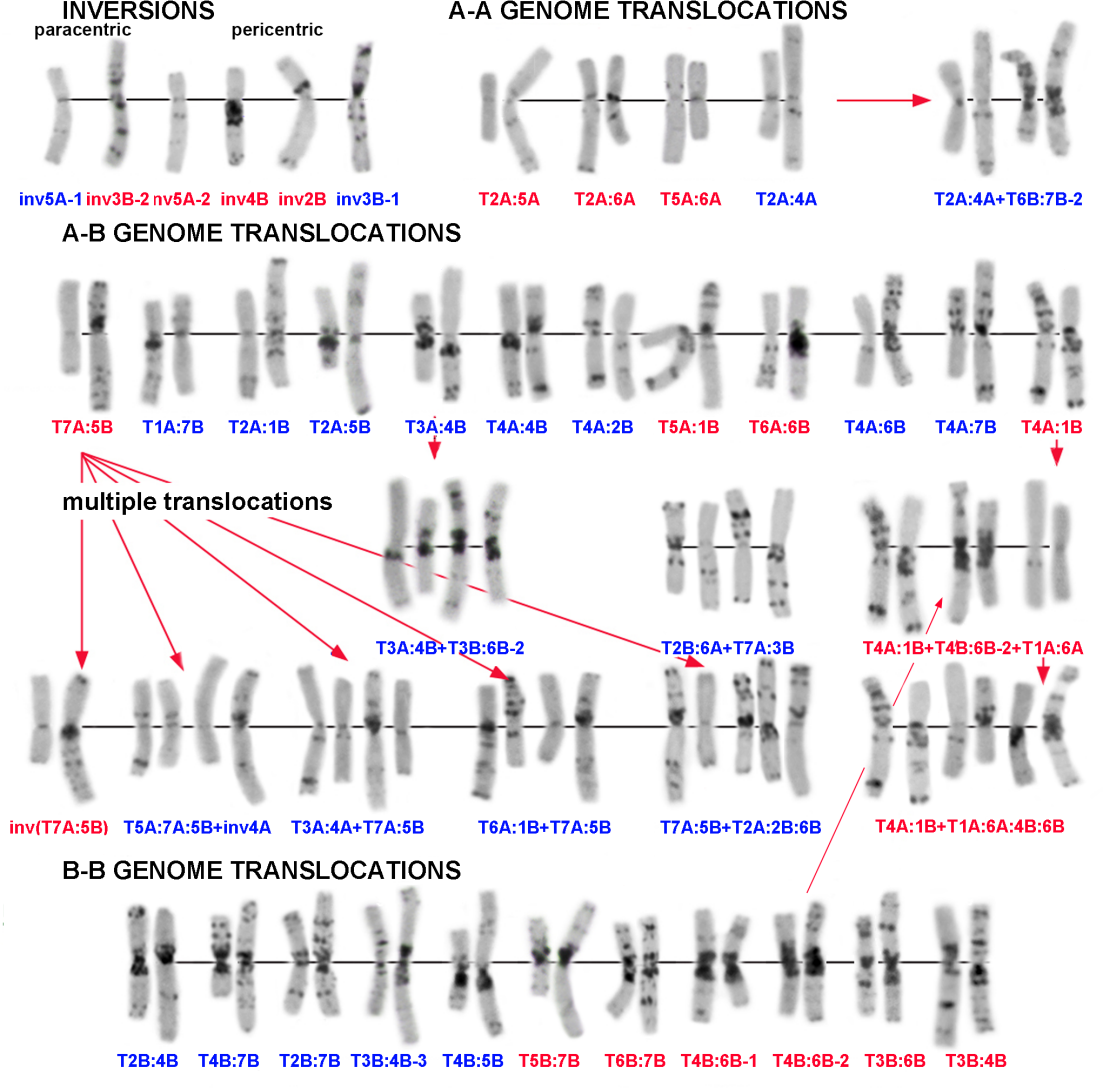
Diversity of chromosomal rearrangements and translocation lineages identified in domesticated emmer. Translocation types are designated according to S3 and S4 Tables. Translocation variants described in previous papers [35,46] are shown in red and novel translocation variants found in this study are indicated in blue. Red arrows define translocation lineages, i.e. a series of related translocations occurring one after another. A detailed description of all translocation variants is given in S3 and S4 Tables.

According to our C-banding analysis, 334 of the 446 lines of *T*. *dicoccon* had normal karyotypes. Forty-three chromosomal rearrangements were identified in the remaining 112 lines (25.1%) (S3 and S4 Tables), from which only 15 were listed in previous catalogues [46,47], and two other translocations were described in [36]. All hitherto known translocations as well as all 26 novel variants identified in our study are shown in Fig 3. Chromosomal rearrangements were represented by single translocations (27 variants), multiple translocations (11 variants), paracentric inversions (two variants) and pericentric inversions (three variants) (Fig 3, S3 and S4 Tables).

Most translocations (27 variants) were found in single lines, and only eight occurred with higher frequencies. The translocation T7A:5B detected in 48 lines from 20 countries was most frequent (S3 Table). Although T7A:5B was especially common in Europe but, interestingly, also detected in few domestic emmer lines from Turkey, Iran, Algeria and Russia (S2*I* Fig; S4*B and* S4*L Fig* and S5*A and* S5*B* Fig). The translocation T6B:7B-1 (3 lines) was found only in emmer lines collected in Bosnia and Herzegovina (S1*B* Fig). T3B:6B-1 (5 lines) was specific for the Asian group (S2*B* and S2*C* Fig and S4*I* Fig), while T4B:6B-1 (4 lines) was unique for the WEM-Sp type (S3*D* Fig). A whole series of translocations (T4A:1B → T4A:1B + T4B:6B → T4A:1B + T4B:6B + T1A:6A → T4A:1B + T4B:6B:T1A:6A) was specific for North African emmer (S3 Table, Fig 3 and S5*E–*S5*J* Fig). Most genotypes with similar translocation variants had similar banding patterns and probably derived from a common ancestral form.

### Chromosomal polymorphisms identified in Isfahan emmer and Colchic emmer

In addition to *T*. *dicoccon*, Dorofeev et al. [13] recognized two other tetraploid domesticated hulled wheat species: I) the Isfahan emmer and II) the Colchic emmer. In our study *T*. *karamyschevii* showed low chromosomal polymorphisms and all accessions had very similar banding patterns (S7*D–*S7*F* Fig), which is typical for endemic taxa. They differed from Georgian *T*. *dicoccon* (S4 *I* Fig) by C-banding patterns of Chr 2A, 3AS, 6A, 2B 3B, 4B and 7B (S4 and S7 Figs). Similarly, low diversity of the C-banding patterns was observed among *T*. *ispahanicum* accessions (S7*A–*S7*C* Fig); which were similar to Iranian *T*. *dicoccon*. No chromosomal rearrangements were observed in both taxa.

Free-threshing durum wheat was included in the analysis as an out-group. Our examination of 10 representative durum cultivars also revealed a low level of C-banding polymorphisms. We detected taxon-specific banding patterns on Chr 3B, 4B, 5B and 7B. Chromosomal rearrangements were not detected (S7*G–*S7*L* Fig).

### Chromosomal polymorphisms of wild emmer, *T*. *dicoccoides*

In order to assess C-banding polymorphisms in wild emmer, 105 lines from all known areas of the current species distribution range were analyzed. Wild emmer wheat showed an extremely high diversity compared to domesticated emmer based on C-banding patterns and translocation polymorphisms (S8 and S9 Figs, S5 Table). We identified population-specific and region-specific polymorphisms. Populations sampled in the eastern part of the species’ distribution range were less diverse than those collected in the Levant, which corresponded to the results obtained using molecular and biochemical markers [3,30,48–50].

According to C-banding patterns, the eastern group can be subdivided into two major chromosomal races: a race comprising populations from the Turkish provinces Kahramanmaraş and Gaziantep (S8*A–*S8*G* Fig); and a race consisting of samples found in the provinces Diyarbakır, Mardin, and Şanlıurfa (S8*H–*S8*Q* Fig), and also from Iran and Iraq (S8*R–*S8*X* Fig). The former race was characterized by highly specific C-banding patterns on Chr 2B, 5B and 6B that were not detected in *T*. *dicoccon*. On the contrary, the same chromosomes in accessions from eastern Turkey, Iran and Iraq resembled the respective chromosomes of domesticated emmer. Several types of chromosomal rearrangements were identified (S5 Table); interestingly, one of them was the translocation T7A:5B found in one Iraqi line (S8*R* Fig).

Wild emmer from the Near East (Israel/Palestine) showed the highest polymorphism (S9 Fig). Some accessions from the surroundings of the Lake of Galilee (subsp. *judaicum* (Vavilov) K. Hammer et A. Filat. [ 51 ]) were clearly distinct from others (S9*A–*S9*E* Fig). These individuals had very similar and highly specific banding patterns of 2A, 4A, 7A, 1B, 4B, 5B, and 7B chromosomes allowing their discrimination from the remaining gene pool of wild emmer. Different populations collected within the Levant showed population-specific C-banding patterns and translocation spectra. The most variable heterochromatin patterns were observed in wild emmer lines from the West Bank of the Jordan River. Most importantly, the translocation T7A:5B was observed in three wild accessions, namely, PI 355455 (Lebanon), k-17157 (Syria) and k-5198 (Israel) (S9*M and* S9*V* Fig).

### Inferring population structure of domesticated emmer

We utilized “chromosomal passport” data to infer the population structure of *T*. *dicoccon*. However, the following peculiarities of chromosomal datasets must be considered. First of all, the unit of segregation and inheritance is the entire chromosome, not a single band. Polymorphic variants of a chromosome could occur due to amplification/ elimination of nucleotide sequences in particular position(s), recombination between heteromorphic chromosomes in hybrids and minor chromosomal rearrangements. Thus, these polymorphic variants can be treated as “chromosomal haplotypes”. Secondly, C-banding analysis is not a proper method for detecting intra-chromosomal recombination. The third constrain lies in the nature of C-banding polymorphism. Variation of C-bands is not a discrete, but gradual process, and this complicates the assessment of C-band sizes. Algorithms allowing working with such datasets are not available. Therefore we adopted statistical approaches that were developed to estimate population structure based on polymorphic markers for the analysis of our results.

**Fig 4:**
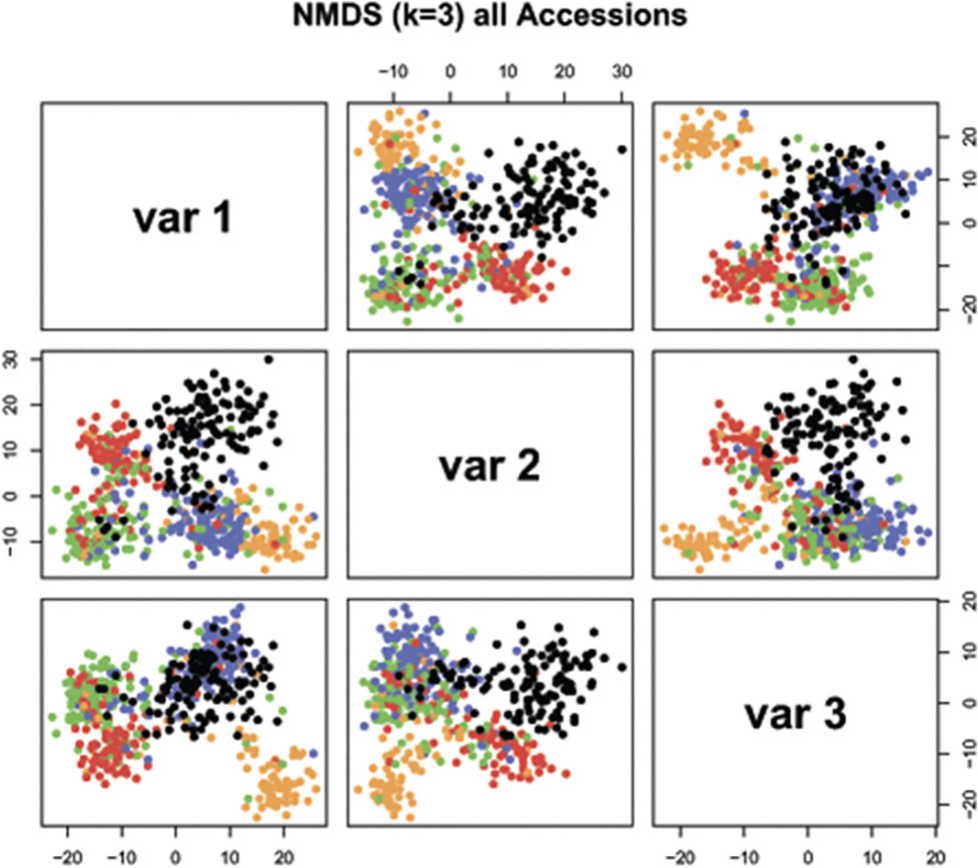
Embedding of 545 emmer wheat accessions into three dimensions by non-metric multidimensional scaling (NMDS). NMDS is based on the Hamming distance calculated on the C-banding patterns (S2 Table). Each point represents a single accession. The four colors blue, red, green and orange are based on k-medoids and represent the European, Balkan, Asian, and Ethiopian, respectively. Additionally, black is used for Dicoccoides and other taxa.

The population structure of domesticated emmer was first estimated on a collection of 421 *T*. *dicoccon* lines using k-medoids and gap statistic (S10 Fig). Shepard plot as well as R^2^ values indicated that it will be reasonable to use nmds statistics (S11 Fig). Four clusters can be distinguished among *T*. *dicoccon* lines (Fig 4).

The first cluster (Fig 4, orange color (tan1)) included 78 lines from Ethiopia (41), Yemen (9), Oman (6), India (5), Turkey (3) and 14 lines from 11 other countries. Most lines (>75%) were also assigned to Ethiopian karyotypic group based on visual karyotype analysis. Eight lines were considered hybrids between Ethiopian and other karyotypic groups (S1 Table), and six lines, predominantly from the Middle East, were visually classified as the WEM-2 type.

The second cluster (green1 color) consisted of 127 lines; of them 102 (>80%) belonged to Asian karyotypic group (85 to Trc-type and 17 to Irn type). Five lines were visually classified as Vol type, whereas 11 lines were considered hybrids between different karyotypic groups.

Cluster # 3 (blue1 color) included 129 lines predominantly of European origin. Based on visual karyotype comparison, 53 lines were assigned to WEM-1 type, 24 to WEM-2 and 17 to WEM-Sp types (altogether >72%). Besides European emmer, this cluster included 5 lines from Asian or Balkan karyotypic groups and 19 lines with hybrid or uncertain origin. Noteworthy, nine Mor lines grouped together with European lines, despite their obvious karyotypic (Fig 2) and morphological [13,26] differences.

Cluster # 4 (Fig 4, red1 color) consisted of 87 lines, all from the Balkan karyotypic group. Sixty one lines were visually assigned to the Bal chromosomal type, 24 to Vol-type and two lines were hybrids between these types.

To get an insight on phylogenetic relationships between five tetraploid wheat taxa a comprehensive collection of 545 lines was analyzed using Neighbor-Joining method. On the resulting tree, the *T*. *dicoccon* gene pool divided into five clusters, and nine “chromosomal types” were defined visually (S12 Fig). The Mor emmer, which was assigned by k-medoid analysis to the European group, was positioned on the NJ_545_ tree within Dicoccoides cluster, as a separate branch. Importantly, there was a good coincidence (87.7%) in the composition of clusters determined by NJ and nmds-analyses (67 lines Ethiopian group, 85 lines of the Balkan group, 106 lines of the Asian group and 114 lines of the European group were assigned to one and the same clusters). Most of the 52 *T*. *dicoccon* lines with inconsistent clustering probably represented hybrids between karyotypic groups or between wild and domestic taxa.

All *T*. *ispahanicum* lines grouped together within the Asian clusters, while *T*. *karamyschevii* clustered together with European emmer. All *T*. *durum* lines fell in the Dicoccoides cluster and grouped together with Mor forms.

Wild emmer clustered separately from domestic forms. Lines from Southern Levant, Northern Levant and subsp. *judaicum* tended to group distinctly within Dicoccoides. Only three domestic lines, all from Jordan were positioned within Dicoccoides cluster on the NJ_545_ tree (S12 Fig, red arrows). All of them are probably hybrids between wild and domestic forms. In turn, three wild emmer lines fell in different clusters of domestic wheat (S12 Fig, green arrows). Importantly, one of these lines, PI 355455 from Lebanon was also placed into domestic gene pool by Luo et al. [30], who used molecular markers.

## Discussion

### Chromosomal passports discriminate four major karyotypic groups within the *T*. *dicoccon* gene pool

Domestication and diffusion of emmer were complex and dynamic processes tightly associated with human history and resulted in the formation of locally adapted populations.

Four major karyotypic groups were discriminated by k-medoid analysis, in accordance with taxonomical [13,26] and molecular studies [30]. Importantly, our data coincided with the result of molecular analysis [30] in the assessment of a set of 49 shared accessions. Thus, chromosome analysis can be used for elucidating phylogenetic relationships among closely related taxa and the reconstruction of diffusion routes of crops. Interestingly, despite unique karyotypic features, the Mor emmer was assigned to the European group. This result, can be caused by i) the relatively small sample size of Mor emmer, constituting of only 2% of the whole population, or ii) the possible hybrid nature of Mor forms, which can be deduced from their karyotypic similarity with durum wheat.

Although characteristic for certain geographic regions, populations of domesticated emmer usually included representatives of more than one karyotypic group at different frequencies. This mixture of karyotypic groups probably came about due to multiple crop introductions by successive waves of colonizing civilizations, which swept across Europe, the Mediterranean and Asia during the second half of the Holocene. However, doubtful origins due to genebank handling errors may also contribute (see S1 File).

Lines belonging to the Asian group were collected, for instance, in north-eastern Turkey, Iran and Transcaucasia, where emmer continued to be cultivated until recent times. Genotypes of the Balkan group were collected from northern Anatolia (Kastamonu province), the Balkans and the Volga region. Our data suggested that these two groups represented the closest domestic emmer forms to the eastern race of wild emmer. In other words, based on C-banding patterns of 2A, 7B, and especially 5B chromosomes, the Balkan and Asian groups probably originated from the eastern wild emmer race. Closest wild relatives were found in the Turkish provinces Diyarbakır, Mardin, and Şanlıurfa (S8 Fig), thus largely supporting the results of Özkan et al. [3].

The European and Ethiopian groups can be discriminated from the Asian and Balkan groups by the C-banding pattern of Chr 2A, which lacks the C-band in the 2AS3 position, but acquired another band in position 2AS5. Most probably, such a specific polymorphic variant of chromosome 2A emerged as a result of a pericentric inversion or transposition of C-band from pericentromeric to the proximal region of the same arm without the disturbing the linkage group. A similar chromosomal variant was found in few *T*. *dicoccoides* lines from various geographical regions: IG 113301 (Iran), k-42632 (Iraq), PI 428063 (Diyarbakır, Turkey), and IG 46528 and PI 355455 (Lebanon), as well as in all lines of the subsp. *judaicum* (lines S9*A–*S9*E* Fig).

Chromosomal rearrangements played an important role in the intraspecific divergence of *T*. *dicoccon*, especially in North Africa. We found 43 different translocation variants, which were mainly restricted to geographic regions. Most translocation variants were found in single individuals and probably originated randomly in particular genotypes, but did not spread out from the area of their emergence. However, translocations, like T7A:5B or T3B:6B may confer selective or adaptive advantages to the respective genotypes. These translocations, especially T7A:5B, are found with high frequency in a broad geographical range. Alternatively, if favorable genes pre- existed in a line before the emergence of translocation, they will be fixed in translocated genotypes due to reproductive isolation and will drag translocation along. The T7A:5B was found in 10% of all emmer lines studied and which was especially frequent in WEM.

Although breeders usually avoid translocations because of decreased hybrid fertility [52] and reduced chromosome recombination [53,54], these genetic resources might be utilized in the future for wheat improvement.

**Fig 5:**
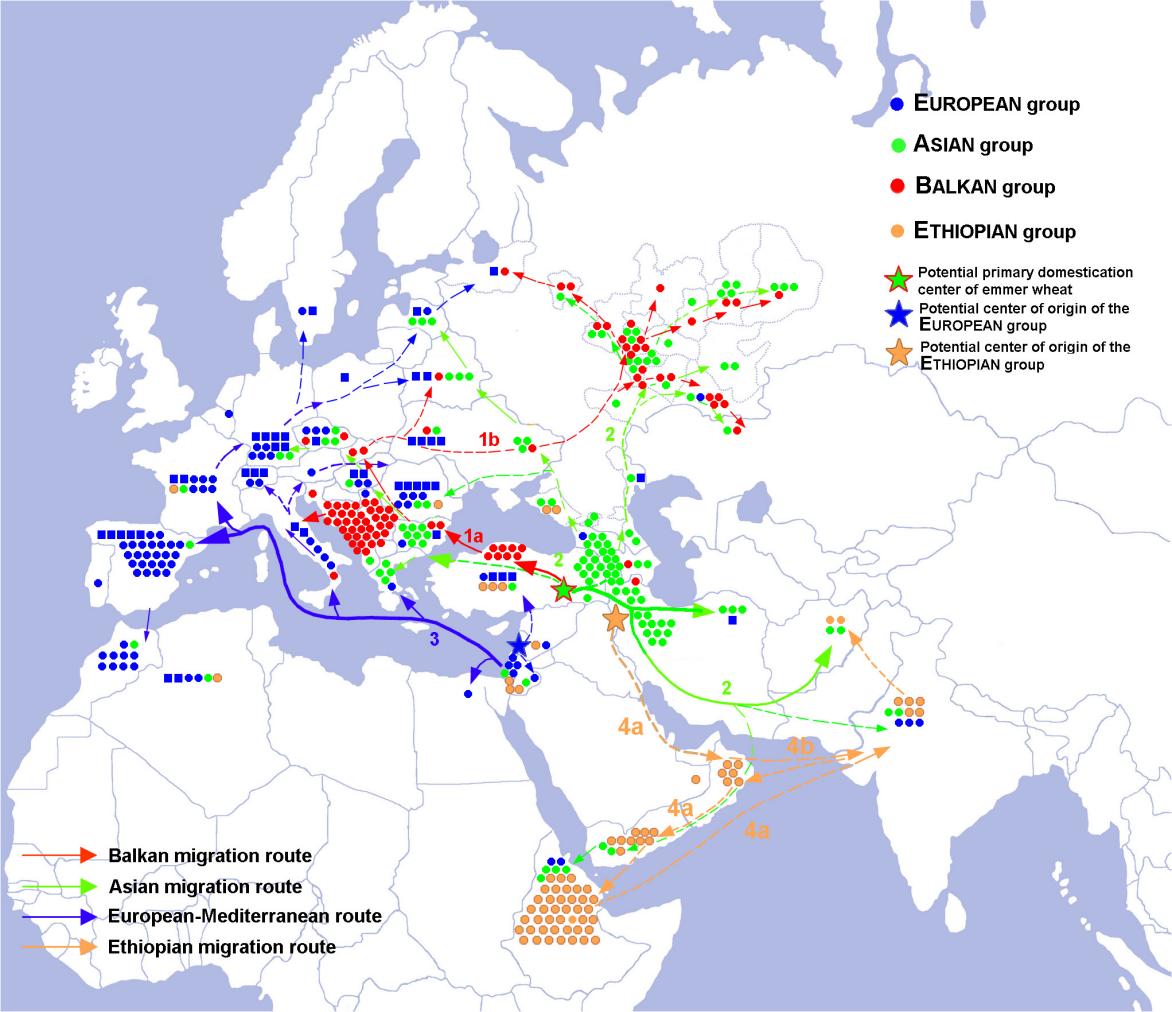
Distribution of karyotypic groups and possible migration routes of domesticated emmer. One dot corresponds to one line studied. The dots are colored according to k-medoids cluster position (see S1 Table). Square dots designate lines carrying the T7A:5B and round dots designate lines lacking the 7A:5B. Solid lines designate migration routes supported by our data, while dashed lines designate hypothetical migration routes, which were not confirmed in our study. Thick lines correspond to major migration routes, thin lines to secondary migration routes; 1a —possible migration route of Balkan emmer from Anatolia to the Balkans; 1b —possible migration route of Vol emmer from the Balkans to the Volga region; 4a —possible migration route of Ethiopian emmer from Zagros to Ethiopia via the Arabian trading route and to India by maritime route; 4b —possible migration route of Ethiopian emmer from Zagros through India and Oman to Ethiopia.

The current geographical distribution of chromosomal groups allows us to suggest four possible major diffusion routes for domesticated emmer (Fig 5).

### The first—the Balkan route—migration of the Balkan group from southeastern Anatolia to the Balkans and subsequently to Eastern Europe (red route in Fig 5)

Karyotypically, the Turkish population from Kastamonu province was very similar to emmer from the Balkans. Following emmer introduction into the Balkans by early farmers [55], the crop probably gradually spread to neighboring territories (Fig 5). A similar migration route has been proposed for common wheat [56,57] and agrees with the archaeological evidences. Subsequently, the spread of the Balkan karyotypic group in the Volga and Ural regions of Russia may have been accompanied by hybridization with local wheat genotypes from other chromosomal groups, in particular with the Asian group. Most lines from Volga region (26) fell in the Balkan cluster (S1 Table, cluster# 4): 25 lines were grouped together with Asian emmer (S1 Table, cluster # 2), and two lines carrying reciprocal translocations—with European. On the NJ_545_ tree the Vol type clustered within the Balkan group as a separate branch (S12 Fig).

### The second route—migration of the Asian group from southeastern Anatolia via Transcaucasia to the Volga region, via the Bosporus to Europe and via Iran to South Asia and India (green route)

Based on our chromosomal passports, we suggested that a second major route (Asian group) may have also started in southeastern Anatolia.

#### (I)—Via Transcaucasia to the Volga

The divergence of Transcaucasian forms from the putative original population of Turkish domesticates was associated with only minor karyotype changes (Fig 2 and S4 Fig). From another side, Asian emmer was karyotypically distinct from other wheat taxa growing in the Caucasus region [58] and exhibited low intra-population diversity [16,37]. These facts suggested that the Asian group probably derived directly from a domesticated Turkish emmer population and that its migration was not associated with hybridization events. According to archeological data, farming was introduced to the southern Caucasus from Eastern Anatolia or northern Iran approximately 7,500 BP, however hexaploid naked wheat became the dominant crop after a few millennia and *T*. *dicoccon* appeared to have survived as minor crop in this area [59]. Indications of farming appeared in archeological sites of the North Caucasus and in north-western plains of the Caspian shore dating to the second half of the 6^th^ millennium BP. These ancient farmers had trading and cultural links with South Caucasian and Middle Eastern communities [6,60]. Relationships between *T*. *dicoccon* lines from southern and northern Transcaucasia are supported by karyotype similarity of the Kabardino-Balkaria and Dagestan forms (S4*H* Fig) with lines from Armenia and Georgia (S4*F*, S4*G* and S4*I* Fig and S4*C* Fig).

The presence and the distribution pattern of Asian emmer in the Volga and Ural regions suggested that it was probably introduced from Transcaucasia northwards, along the Volga River. Emmer was an important crop in the Middle Volga region and the Ural, and was cultivated in Bashkiria, Tatarstan, Chuvashia, and Perm regions of Russia till the beginning of the 20^th^ century [15,18]. Twenty five of the 51 *T*. *dicoccon* lines from the Volga and Ural regions belonged to the Asian group. They co-occurred with the Balkan lines (26 lines) in the region from Samara to Nizhny Novgorod (Fig 5). Upstream of the Volga, the ratio of Asian / Balkan lines decreased gradually. The Balkan lines were probably the descendants of emmer that was brought into middle Volga from the Balkans, whereas the Asian lines probably originated from Transcaucasian emmer. According to visual karyotype comparison, Asian and Balkan lines often produce hybrids with various combinations of parental chromosomes (S2*H*, S2*L*, S2*R and* S2*V* Fig), which further complicates the analysis of the ancestry of Volga emmer.

#### (II) Via the Bosporus to Europe

The Asian type also occurred in Bulgaria, Greece and Albania (Fig 5, S1*E–*S1*H*, S1*J*, S1*Q and* S1*V* Fig and S3*W and* S3*X* Fig). We suggested that their ancestral forms probably reached south-eastern Europe directly from Anatolia, probably admixed with other karyotypes, especially the Balkan type.

#### (III) Via Iran to India

The Asian karyotypic group was broadly distributed in Iran and also found in Afghanistan and India (S4*P*, S4*R* and S4*W* Fig). Several lines from north-western Iran that we visually assigned to the Irn type were distinct from the remaining Asian lines by a prominent centromeric and relatively small 2BS5 block (Fig 2). On the NJ_545_ tree they tended to group together within a common Asian cluster (S12 Fig). The Irn type might have originated as a result of gene flow from Iranian wild emmer to the Asian group of domesticated emmer in the eastern part of the Fertile Crescent.

Unfortunately, collection site information was missing for most domestic Iranian lines and only few emmer accessions were available from countries further east. This impeded tracing emmer wheat dispersion east of Iran. However, our data suggested that this migration was associated with cytogenetic changes probably due to hybridization between different seed stocks along ancient trading routes.

### Third route—from the southern Levant to Europe and via the Iberian Peninsula or from Near East to Africa (European group, blue route)

Our data suggested that the domestic European emmer group was not a direct descendant of wild emmer wheat from Anatolia. Instead, European emmer could have been domesticated independently from Asian and Balkan groups, and probably originated directly from western wild emmer from within the Levant (S6*A* and S6*B* Fig and S9*Q* Fig).

We here proposed that the dispersion of European emmer to the Mediterranean and Western Europe started in the southern Levant (Fig 5). Archaeologists have identified two diffusion routes of agriculture into Europe from the Fertile Crescent, and in both cases the spread of agriculture involved several waves of colonists but also local populations, who adopted farming with introduced crops [61]. The first route was going through Turkey, the Balkans, Central Europe and into Western Europe following major river systems with emmer and einkorn [62,63]. So one may expect that Neolithic European emmer will be of the Balkan type. Indeed, the Balkan and Asian-type lines rarely occurred in West European countries (Fig 5), and these forms could be the remnants of the earliest migration wave. However, the majority of WEM lines belong to European karyotypic group. They were probably introduced later, as a result of migration going as a maritime route into southern Europe, Italy, France, Spain and Portugal, where emmer wheat was found mixed with free-threshing wheat. An independent migration route of common wheat to the Mediterranean and West-Europe has been suggested [56,57] based on analysis of microsatellite allelic diversity. The geography of these countries and the specific climatic conditions may have played a major role in the differentiation of germplasm [57]. Most probably, emmer genotypes belonging to European group may confer some selective/adaptive advantages and therefore replaced the Balkan emmer in these territories.

The maritime route is supported by the similarity of *T*. *dicoccon* from the Near East with the European group (S5 and S6*C–*S6*K* Fig) and by the presence of the 7A:5B translocation, which occurred at high frequency in European emmer and was found in few wild emmer accessions from the Levant (S9*M and* S9*V* Fig). Wild emmer from the Southern Levant was characterized by higher karyotype variation compared to Anatolian *T*. *dicoccoides* (S8 and S9 Figs). This might have facilitated the broader genetic background of the European group compared to all other karyotypic groups of domestic emmer. Karyotype diversity of the European group could have been additionally broadened by hybridization and recombination with lines from other karyotypic groups that grew sympatrically.

Vast majority of emmer lines from WEM countries (63 of 77) were attributed to group # 3 based on the k-medoid analysis (Fig 4, S1 Table). Visual chromosome comparison allowed dividing them into three chromosomal types, WEM-1, WEM-2, and WEM-Sp, which tended to group together within the European cluster on the NJ_545_ tree (S12 Fig). Lines carrying the 7A:5B translocation positioned within the WEM-1 group. High karyotypic similarity of these lines was probably caused by reproductive isolation, because translocations can lead to hybrid depression [64] and thus create genetic barriers between genotypes growing in one population. In addition, translocations prohibit chromosome recombination [53], and translocated chromosomes will not be modified compared to the original type (S1*G*, S1*L*, S1*N*, S1*R and* S1*U* Fig). T7A:5B probably originated in the western race of wild emmer in the Levant. In turn, this translocation could also have occurred in domestic emmer in Southern Levant and then been introgressed into local wild populations due to gene flow.

According to our study, domestic emmer in Spain belonged to European chromosomal group, and among them we visually discriminated the WEM-Sp type (S3*A–*S3*E* Fig). This type was indigenous for Spain and was found in 18 out of 29 lines investigated—mostly from Asturias and Navarra. Three lines carried a T4B:6B-1 translocation (S3 Table, line S3*D* Fig). On the NJ_545_ tree the WEM-Sp lines were grouped together within European group (S12 Fig).

#### The North African genepool

Archaeological finds suggested that agriculture was initially introduced into Morocco from the Iberian Peninsula [65], but there may have been many other later introductions.

Most lines from Algeria and Morocco showed unique banding patterns defining the Mor emmer, which were attributed to European karyotypic group by k-medoid analysis, but occupied a distinct position on the NJ_545_ tree. Translocations played an especially important role in the evolution of Mor emmer, as a series of four consequent translocations has been identified. Two lines carried T7A:5B and two lines T4A:1B. Genotypes with significantly rearranged karyotypes prevailed in Morocco (6 out of 10 lines), and all of them were derivatives of T4A:1B. Based on our findings, we suggested that Northwestern African emmer was probably formed by introduction from southern Spain followed by hybridization with local durum wheat and subsequent chromosomal rearrangements. This might have happened much later, when durum wheat was introduced into Africa. Few lines belonging to the Asian group could have been brought in by the Phoenicians as Carthage was a center of commerce in North Africa.

### Fourth route—from the Fertile Crescent via Oman to Ethiopia and to India (Ethiopian group, orange route)

Ethiopian emmer was karyotypically well separated from other karyotypic groups and possessed several diagnostic chromosomes. It was similar to the European group in the C-banding patterns of Chr 2A and 7B, however these groups differed in diagnostic features on all other chromosomes. The combination of two polymorphic variants of Chr 1B lacking the marker C-bands 1BS5 but harboring the additional proximal band 2BS3 was found to be unique for the Ethiopian group. These chromosomal variants were never found combined in wild emmer lines. However, either one or the other chromosomal variant occasionally occurred in wild lines from the southern Levant (S9*T*, S9*U and* S9*W* Fig), but also from Iraq and Iran (S8*R*, S8S, S8*U and* S8*V* Fig). Thus, our chromosomal passport data as well as other data available from literature [30], do not provide direct evidences, when and where the Ethiopian group originated. However, based on i) the geographical distribution of rare polymorphic chromosomal variants (2A, 1B and 2B chromosome heterochromatin patterns), as well as ii) considering the geographic distribution of the Ethiopian group, we propose that this group could have originated in Northern or Northeastern Mesopotamia. Additionally, gene flow from western wild emmer has probably contributed to the formation of this chromosomal group, thus supporting the reticulate domestication model by Civan et al. [29].

Arguments in favor of wild emmer having been independently domesticated in the Zagros region of western Iran are quite strong. Wild emmer was known to have been exploited in this area at the site of Chogha Golan about 11,400 years ago and by 9,800 years ago it was domesticated at the same site [66]. Emmer finds from southern Pakistan at the sites of Mehrgarh possibly date back to 9,000 years ago [67]. By 8,200 years ago emmer is found at the site of Jeitun in Turkmenistan [68]. Finds of wheat from India are much later and date to about 4,500 BP. However, these are often naked type wheats, but Fuller reports on some Neolithic wheat finds including those from southern India [69].

We have identified the Ethiopian group in few domesticates collected in the Near East, and in all lines from Saudi Arabia, Oman, and most lines from Yemen and (S4*O*, S4*S and* S4*U* Fig and S6*O–*S6*S and* S6*U* Fig). Our data thus provide further evidence that domestic emmer could have reached Ethiopia through Yemen via ancient trade routes (Fig 5, orange routes 4a, b). Also botanical characters supported the close relationships between *T*. *dicoccon* lines from Ethiopia and Yemen. These races were described as subsp. *abyssinicum* Vavilov [13,26].

Further support comes from domesticated barley, where the same migration route into Ethiopia was postulated by Pomortsev et al. [70]. These authors also showed that only few “founder” genotypes gave rise to the current limited diversity of barley in Ethiopia. We also observed very limited C-banding polymorphisms among Ethiopian *T*. *dicoccon* materials compared to samples from other geographic regions. Nucleotide diversity based on partial DNA sequences of single-copy nuclear genes also pointed to a limited genetic background of Ethiopian emmer [71]. As one karyotypic variant significantly dominated among domestic emmer lines, we suggested that Ethiopian emmer first underwent a severe genetic bottleneck, followed by local adaptation. Few Ethiopian lines belonging to the Asian or the European types could have been introduced recently, or they were probably mistaken during *ex situ* maintenance.

Earlier studies suggested that Ethiopian emmer was probably introduced to Ethiopia through Egypt and Sudan some 5,000 years ago [72]. From here domestic emmer probably reached the Arabian Peninsula [6]. A possibility of emmer introduction through Egypt cannot be ruled out, because we had access to just one emmer accession from this country. According to Stoletova [15], emmer that was still cultivated by very few Egyptian farmers in the beginning of the 20^th^ century was distinct from the remnant of emmer plants excavated from Egyptian pyramids, being more similar to modern Palestinian or Iranian forms. Therefore, the direct ancestry of emmer forms that were grown in Egypt in the recent past from Neolithic emmer is improbable.

The close morphological and genetic relationship between Ethiopian and Indian domestic emmer is well documented [13,26,30,31]. As hypothesized earlier, emmer was introduced into India during the 4^th^ or 3^th^ millennia BC [6]. It was suggested that emmer could have reached Kashmir: I) from the Middle East through Iran [73], and II) from Ethiopia via maritime trade [30]. We here provide evidence that both routes are possible: We found two lines in Afghanistan belonging to the Ethiopian group (S4 *O* Fig), although they were absent from Iran. On the basis of phenological and morphological examinations, Hammer et al. [39] assigned emmer sampled in Oman to the Asian eco-geographic group. However these lines also shared some morphological similarities with Abyssinian forms (K. Hammer, personal communication), thus providing support for a close relationship between lines collected in Ethiopia, Oman and India.

### The origin of *T*. *ispahanicum* and *T*. *karamyschevii*

In contrast to *T*. *dicoccon*, Colchic emmer and Isfahanian emmer had a very limited geographical distribution and are currently only maintained *ex situ* in genebanks, with 71 and 53 accessions in 25 and 16 genebanks, respectively [25]. Both taxa differ from *T*. *dicoccon* in several morphological traits.

*Triticum ispahanicum* was found in few villages from the Vazak Canton, Faridan district of the Isfahan province, Iran at an altitude of 2,000–2,500 meters. Isfahanian emmer was collected by Kuckuck in 1952–1954 [74], a French expedition in 1957 [75], and a Japanese expedition [76]. Afterwards, despite intensive searches, *T*. *ispahanicum* was never found again in cultivation [41]. Close similarities in chromosomal characteristics suggested that *T*. *ispahanicum* cannot be treated as an independent taxonomical unit; it should be considered a variety of *T*. *dicoccon* that was probably derived from Asian domesticated emmer as a result of gene mutation affecting the glume length (*P*_*2*_ gene, [77]).

*Triticum karamyschevii* was cultivated in Western Georgia, often in mixed stands with *T*. *macha*. The origin of *T*. *karamyschevii* is less clear. This taxon has a dense spike, which is prone to branching, and plants are morphologically similar to *T*. *macha* [13]. Some authors suggested that *T*. *karamyschevii* was domesticated locally from wild emmer populations [13,78]. However this is unlikely, because *T*. *dicoccoides* does not occur naturally in Georgia [3]. Alternatively, the taxon could have originated from *T*. *dicoccon* and then been selected for Western Georgia (Dekaprelevich 1941, cit. in [13]). However, the following facts contradicted this assumption: Although the remnants of domesticated emmer have been found in many archeological sites from Transcaucasia [78], this taxon was not recorded in Western Georgia, where *T*. *karamyschevii* is distributed [79]. Our data showed that these two wheats significantly differed in their C-banding patterns, and *T*. *karamyschevii*, like Georgian *T*. *dicoccon*, displayed low karyotypic polymorphism. Karyotypic differences and low polymorphism due to geographic isolation suggested that *T*. *karamyschevii* is not a direct descendant of Georgian emmer. Some karyotypic similarities of *T*. *karamyschevii* with the European group of domesticated emmer, especially in the C-banding pattern of Chr 2A, indicated that Colchic emmer could have originated from the European group, which was introduced into Georgia in the past.

### New insights into the origin of *T*. *dicoccoides* subsp. *judaicum*

In our analysis, 102 of 105 wild emmer lines formed a common cluster on the NJ_545_ tree (S12 Fig, dicoccoides),which was further subdivided corresponding to the wild emmer races defined by Özkan et al. [3], which are in good agreement with Luo et al. [30].

Morphologically distinct from other wild emmer lines, *T*. *dicoccoides* subsp. *judaicum* was hypothetically thought to derive as a result of hybridization of *T*. *dicoccoides* with durum wheat [80] or with *T*. *dicoccon*. All lines belonging to subsp. *judaicum* were karyotypically very similar and showed specific banding patterns on Chr 2A, 7A, 4B, 5B, and 7B, allowing their discrimination from all other wild [58] and domesticated wheats. On the NJ_545_ all *judaicum* lines grouped together within a common cluster dicoccoides (S12 Fig). Subsp. *judaicum* was similar to European and especially to Ethiopian domesticated emmer in the C-banding pattern Chr 2A, indicating their relatedness. Based on morphological similarity, Vavilov [81] suggested a possible ancestry of domesticated emmer from subsp. *judaicum*. However, this hypothesis was later rejected [3,30]. Based on our data, we propose that subsp. *judaicum* evolved naturally in the Levant, and not from hybridization between *T*. *dicoccoides* and *T*. *durum*.

## Supporting Information

S1 Fig Chromosomal polymorphism of *T*. *dicoccon* from Eastern Europe: Montenegro (*a*), Bosnia and Herzegovina (*b*, *c*), Serbia (*d*), Albania (*e*), Bulgaria (*f*–*i*), Romania (*j–l*), Hungary (*m–o*), former Czechoslovakia (*p–t*), Poland (*u*), Latvia (*v–x*). a—PI 434996, b—PI 434995b, c—PI 434999b, d—PI 362501b, e—TRI 17634, f—k-35926, g—PI 295065, h—INRA 27089, i—k-14236, j—PI 306535, k—PI 306531, l—IG 45926, m—PI 252527, n—INRA 26654, o—PI 252528, p—PI 352369 (Czech Republic), q—k-29606-5, r—INRA 26651, s—TRI 9868 (Czech Republic), t—TRI 10324 (Slovakia), u—PI 286061, v—k-38185-2, w—k-38185-1; x—k-19091. 1A–7B —chromosomes. Chromosomal rearrangements are indicated with arrows and designated according to S3 and S4 Tables.
(TIF)

S2 Fig Chromosomal polymorphism of *T*. *dicoccon* from republics of the former USSR: Belarus (*a–b*), Ukraine (*c–e*), Russia (*f–v*), Kazakhstan (*w*, *x*). a—k-39300-3, b—k-18774-4; c—k-14999, d—k-15007, e—k-19361, f—PI 94676, g—IG 45355 (Krasnodar Region), h—k-47795, i—k-9934 (Leningrad1 Region), j—IG 45354, k—k-94660-1 (Yaroslavl Region), l—k-30728-3 (Nizhny Novgorod Region), m—k-7492 (Vyatka Region), n—k-25516-6 (Chuvashia), o—k-6246-2, p—k-6249 (Ulyanovsk Region), q—k-42065 (Udmurtia), r—k-7490, s—k-33153 (Perm Region), t—k-10456 (Tatarstan), u—k-7508-3 (Yekaterinburg Region), v—PI 94616 (Ural Region), w—k-46995, x—k-34678. 1A–7B —chromosomes. Chromosomal rearrangements are indicated with arrows and designated according to S3 and S4 Tables. ^1^Leningrad Region—region around St. Petersburg.
(TIF)

S3 Fig Chromosomal polymorphism of *T*. *dicoccon* from Western Europe and Mediterranean countries: Spain (*a–e*), Portugal (*f*), Great Britain (*g*), Belgium (*h*), Sweden (*i*), France (*j–l*), Germany (*m–o*), Switzerland (*p*, *q*), Austria (*r*), Italy (*s–v*), Greece (*w*, *x*). a—PI 275999, b—PI 276013b, c—k-21177, d—PI 191091, e—k-20541, f—IG 45337, g—PI 278644, h—PI 532322, i—TRI-19294, j—INRA 6807, k—INRA 26648, l—k-21589c, m—INRA 26642, n—k-1730, o—k-21433, p—k-12946, q—PI 355467, r—PI 323435, s—INRA 27097, t—INRA 27088, u—INRA 27096, v—k-21419, w—IG 88757, x—IG 94682. 1A–7B —chromosomes. Chromosomal rearrangements are indicated with arrows and designated according to S3 and S4 Tables.
(TIF)

S4 Fig Chromosomal polymorphism of *T*. *dicoccon* from Transcaucasia and Asia: Turkey (*a–e*), Armenia (*f*, *g*), Kabardino-Balkaria (*h*); Georgia (*i*), Iran (*j–m*), Uzbekistan (*n*), Afghanistan (*m–o*), India (*p–u*), China (*v–x*). a—PI 355507, b—TRI 584, c—IG 45336, d—INRA 27113, e—TA 10480, f—k-13648, g—k-14039, h—k-38152, i—TRI 16608, j—PI 624908, k—TA 10504, l—k-7146, m—k-45542a, n—k-51768, o—IG 45318a, p—IG 88750, q—IG 45318b, r—k-44167, s—k-44154, t—k-46482, u—k-45514c, v—INRA 23799, w—PI 79899, x—KU-112. 1A–7B —chromosomes. Chromosomal rearrangements are indicated with arrows and designated according to S3 and S4 Tables.
(TIF)

S5 Fig Chromosomal polymorphism of *T*. *dicoccon* from Africa: Algeria (*a–f*), Morocco (*g–l*), Egypt (*m*), Ethiopia (*n–x*). a—INRA 26893, b—INRA 26894, c—INRA 26895, d—INRA 26896, e—INRA 26897, f—INRA 27898, g—IG 127703, h—k-15837, i—IG 45317, j—k-22246, k—k-15840a, l—k-15840b, m—27970, n—IG 45393, o—INRA 27098, p—INRA 27100, q—k-43771, r—IG 45315, s—IG 45303a, t—IG 45303b, u—INRA 27234, v—k-19256, w—INRA 27087, x—PI 577791. 1A–7B —chromosomes. Chromosomal rearrangements are indicated with arrows and designated according to S3 and S4 Tables.
(TIF)

S6 Fig Chromosomal polymorphism of *T*. *dicoccon* from the Middle East and Arabia: Jordan (*a*, *b*), Syria (*c*, *d*), Palestine and Israel (*e–k*), Saudi Arabia (*l*, *m*), *Oman* (*o–s*), Yemen (*t–x*). a, b—IG 45254a, b, c—PI 352348, d—PI 355498, e—IG 45363, f—IG 45444, g—TRI 3424, h—TRI 16880, i—PI 352367, j—PI 355496, k—TRI 16879, l—INRA 27085, m—PI 94664, n——IG 45091, o—IG 45069, p—IG 45070, q—IG 45073, r—IG 45068, s—PI 532305, t—TA 10514, u—TRI 28027a, v—TRI 28072a, w—IG 99244, x—k-25459. 1A–7B —chromosomes. Chromosomal rearrangements are indicated with arrows and designated according to S3 and S4 Tables.
(TIF)

S7 Fig Chromosomal polymorphism of *T*. *ispahanicum* (*a–c*), *T*. *karamyschevii* (*d–f*) and *T*. *durum* (*g–l*). *a—*TRI 6177, *b—*TRI 7260, *c—*TRI 7117, *d—*TRI 4568, *e—*TRI 7496, *f—*TRI 4607, *g—*PI 470897 (Algeria), *h—*‘Krasnokutka-10’ (k-62422, Russia), *i* and *j*—landraces from Egypt, *k—*‘Chakinskaya-226’ (k-39099, Russia), *l—*‘Valentina’ (k-62650, Russia).
(TIF)

S8 Fig Karyotype diversity of the eastern race of *T*. *dicoccoides*. Turkey (*a–q*), Iraq (*r–u*), Iran (*v–x*). a—KU-1972b, b—KU-1991, c—KU-1952, d—KU-1955 (Kahramanmaraş), d—IG 116171, e—IG 11671, f—IG 116174, g—IG 116181, h—IG 116179b (Gaziantep), i—IG 46149, j—IG 46183, k—IG 46185, l—IG 46171 (Şanlıurfa), m—IG 46250, n—PI 428051, o—PI 428063, p—PI 428045 (Diyarbakır), q—PI 428145 (Mardin), r—k-46632, s—IG 109085, t—IG 131232, u—IG 109085, v—KU-8942, w—IG 113301, x—IG 113302. 1A–7B —chromosomes. Chromosomal rearrangements are indicated with arrows and designated according to S5 Table.
(TIF)

S9 Fig Karyotype diversity of the Central-Eastern race of *T*. *dicoccoides*. Israel (*a–j; a–e—*subsp. *judaicum*), Lebanon (*k–n*), Jordan (*o–s*), Syria (*t–x*). a—UH-J4-2, b—UH-J5-1, c—PI 467019, d—UH-G1-6, e—PI 467014, f—PI 414719, g—TA1057, h—UH-H-8-1, i—UH-NM6, j—PI 538699, k—IG 46531a, l—IG 46531b, m—PI355455, n—IG 46526a, o—IG 115808a, p—IG 139130, q—IG 46486a, r—IG 139129, s—IG 115807, t—IG 119450, u—IG 119408, v—k-17157, w—IG 45506, x—IG 117894. 1A–7B —chromosomes. Chromosomal rearrangements are indicated with arrows and designated according to S5 Table.
(TIF)

S10 Fig Gap curve of k-medoids for estimating the number of clusters in the 421 emmer wheat-accessions. Four main clusters (black filled circle) were identified using gap statistic and the criteria of Tibshirani [43] with twice standard error (cf. material and methods section).
(TIF)

S11 Fig Shepard plot comparing the original and embedded Hamming distance of the 545 emmer wheat-accession. (TIF)

S12 Fig Neighbor-Joining-Tree (NJ_545_) of karyotype differences between 545 tetraploid wheat lines belonging to five taxa. The NJ-tree is based on the C-banding patterns (S2 Table). The four colors blue, red, green and orange are based on k-medoids (Fig 4) and represent the European, Balkan, Asian, and Ethiopian groups, respectively. Additionally, black is used for Dicoccoides and other taxa. Edges are colored with the unique color of all leaves in the respective subtree or otherwise grey. The line codes are the same as in S1 Table. Groups of accessions are named according to the origin of the material: Dicoccoides: western/southern race, ssp. *judaicum*, eastern/northern race, durum wheat, Mor emmer; Ethiopian group; European group, including Wem-1, Wem-2, WEM-Sp types and *T*. *karamyschevii;* Balkan group, including Bal and Vol types; Asian-group, including Irn and Trc types and *T*. *ispahanicum*). Lines of *T*. *dicoccoides* which clustered within domesticated lines are indicated with green arrows; *T*. *dicoccon* lines which clustered within wild emmer are indicated with red arrows.
(TIF)

S1 File Germplasm. (DOCX)

S1 Table Overview of *T*. *dicoccon* accessions and their origin. (DOCX)

S2 Table Chromosomal passports of 545 tetraploid wheat lines. Columns: positions of 101 C-bands according to idiogram (1AL3–7BL23; Fig 1); rows dcn001–kar006—code of the line (dcn = *T*. *dicoccon;* dcs = *T*. *dicoccoides;* dur = *T*. *durum;* isp = *T*. *ispahanicum;* kar = *T*. *karamyschevii*). 0—bands absent; 1—very small band; 2—distinct band; 3—large and very large band.
(XLSX)

S3 Table List of chromosomal rearrangements identified in *Triticum dicoccon* and their geographical distribution. Translocations described earlier. No.: ^1^chromosomal rearrangements described in [46]; ^2^chromosomal rearrangements described in [35], Translocation type: the superscripts correspond to the numbers of the respective translocations in a catalogue [46]. Structure of rearranged chromosomes—formed as a result of translocation/inversion; #: total number of lines carrying the respective translocation type; Geographical distribution: country in which the translocation type was identified (the number of lines carrying the respective translocation type is given in parenthesis).
(DOCX)

S4 Table List of chromosomal rearrangements identified in *Triticum dicoccon* and their geographical distribution. Novel translocations not included in the catalogue [46].
(DOCX)

S5 Table Translocations identified in *T*. *dicoccoides*. (DOCX)
