# Supplementary figures and images for: Chromosomal Passports Provide New Insights into Diffusion of Emmer Wheat
Source: PLoS One. 2015 May 29;10(5):e0128556. doi: 10.1371/journal.pone.0128556 (PMC4449015; doi:10.1371/journal.pone.0128556)

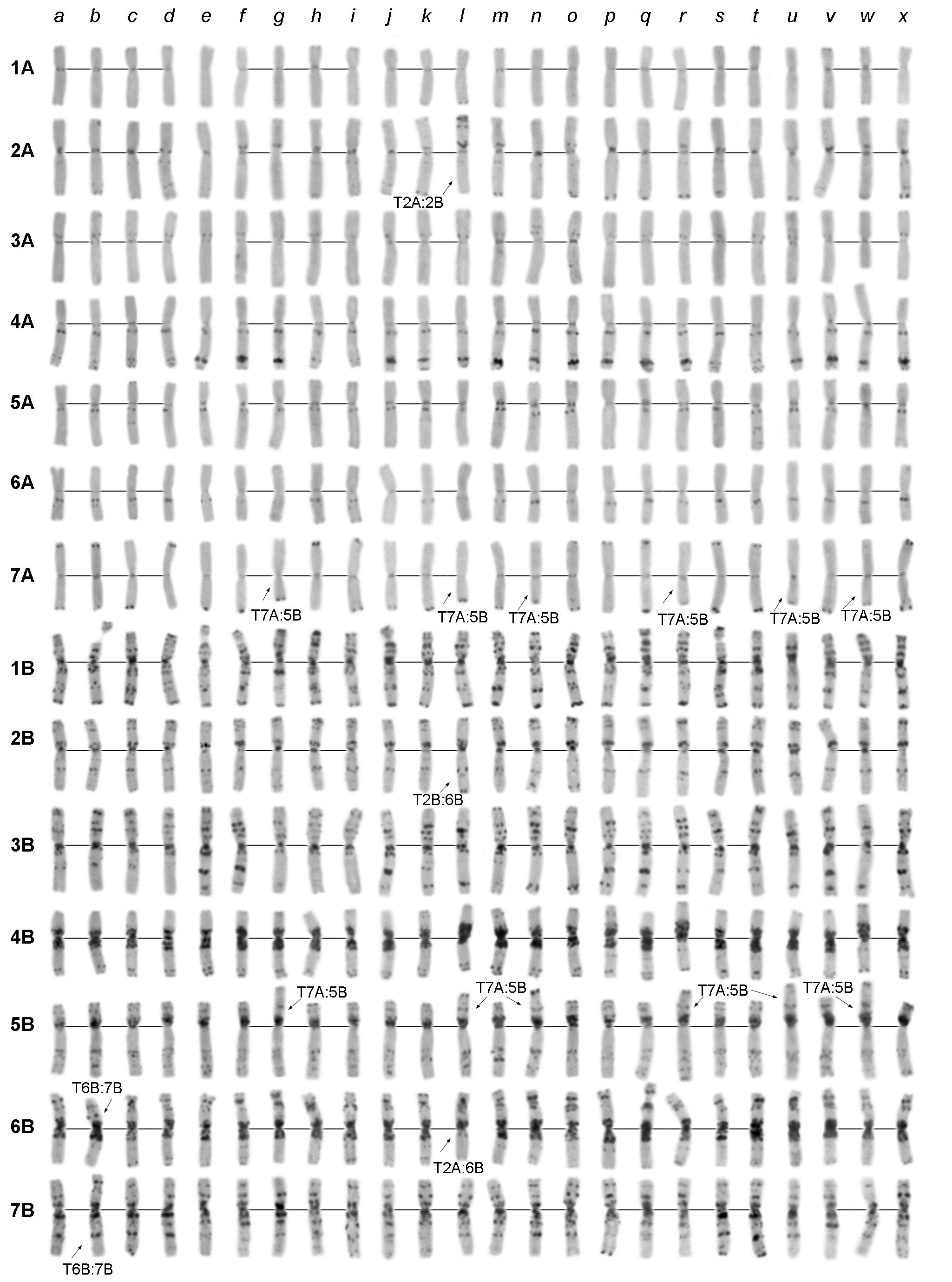

Supplement: S1 Fig — a—PI 434996, b—PI 434995b, c—PI 434999b, d—PI 362501b, e—TRI 17634, f—k-35926, g—PI 295065, h—INRA 27089, i—k-14236, j—PI 306535, k—PI 306531, l—IG 45926, m—PI 252527, n—INRA 26654, o—PI 252528, p—PI 352369 (Czech Republic), q—k-29606-5, r—INRA 26651, s—TRI 9868 (Czech Republic), t—TRI 10324 (Slovakia), u—PI 286061, v—k-38185-2, w—k-38185-1; x—k-19091. 1A–7B —chromosomes. Chromosomal rearrangements are indicated with arrows and designated according to S3 and S4 Tables. (TIF) [file pone.0128556.s001.tif]

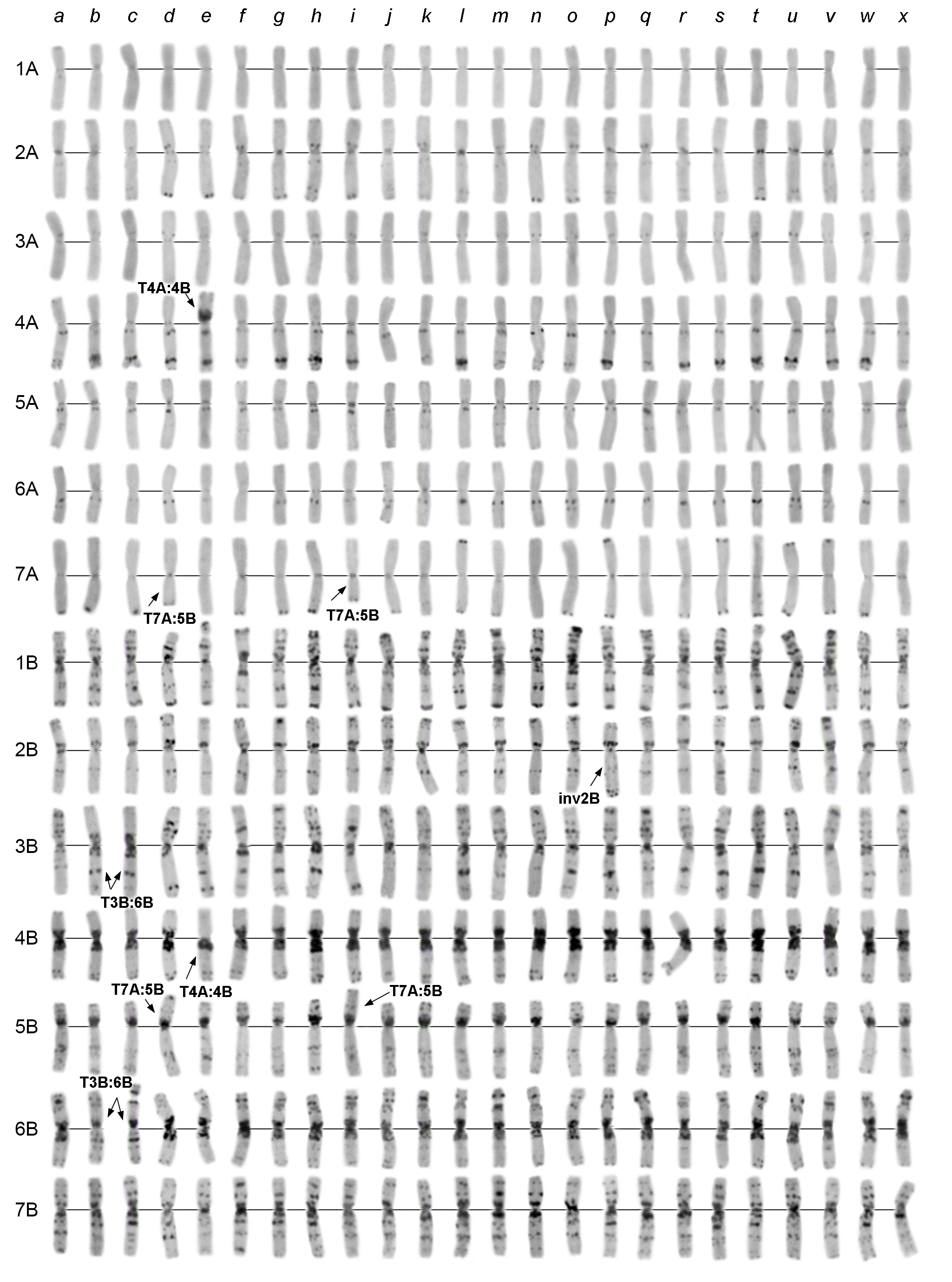

Supplement: S2 Fig — a—k-39300-3, b—k-18774-4; c—k-14999, d—k-15007, e—k-19361, f—PI 94676, g—IG 45355 (Krasnodar Region), h—k-47795, i—k-9934 (Leningrad1 Region), j—IG 45354, k—k-94660-1 (Yaroslavl Region), l—k-30728-3 (Nizhny Novgorod Region), m—k-7492 (Vyatka Region), n—k-25516-6 (Chuvashia), o—k-6246-2, p—k-6249 (Ulyanovsk Region), q—k-42065 (Udmurtia), r—k-7490, s—k-33153 (Perm Region), t—k-10456 (Tatarstan), u—k-7508-3 (Yekaterinburg Region), v—PI 94616 (Ural Region), w—k-46995, x—k-34678. 1A–7B —chromosomes. Chromosomal rearrangements are indicated with arrows and designated according to S3 and S4 Tables. 1Leningrad Region—region around St. Petersburg. (TIF) [file pone.0128556.s002.tif]

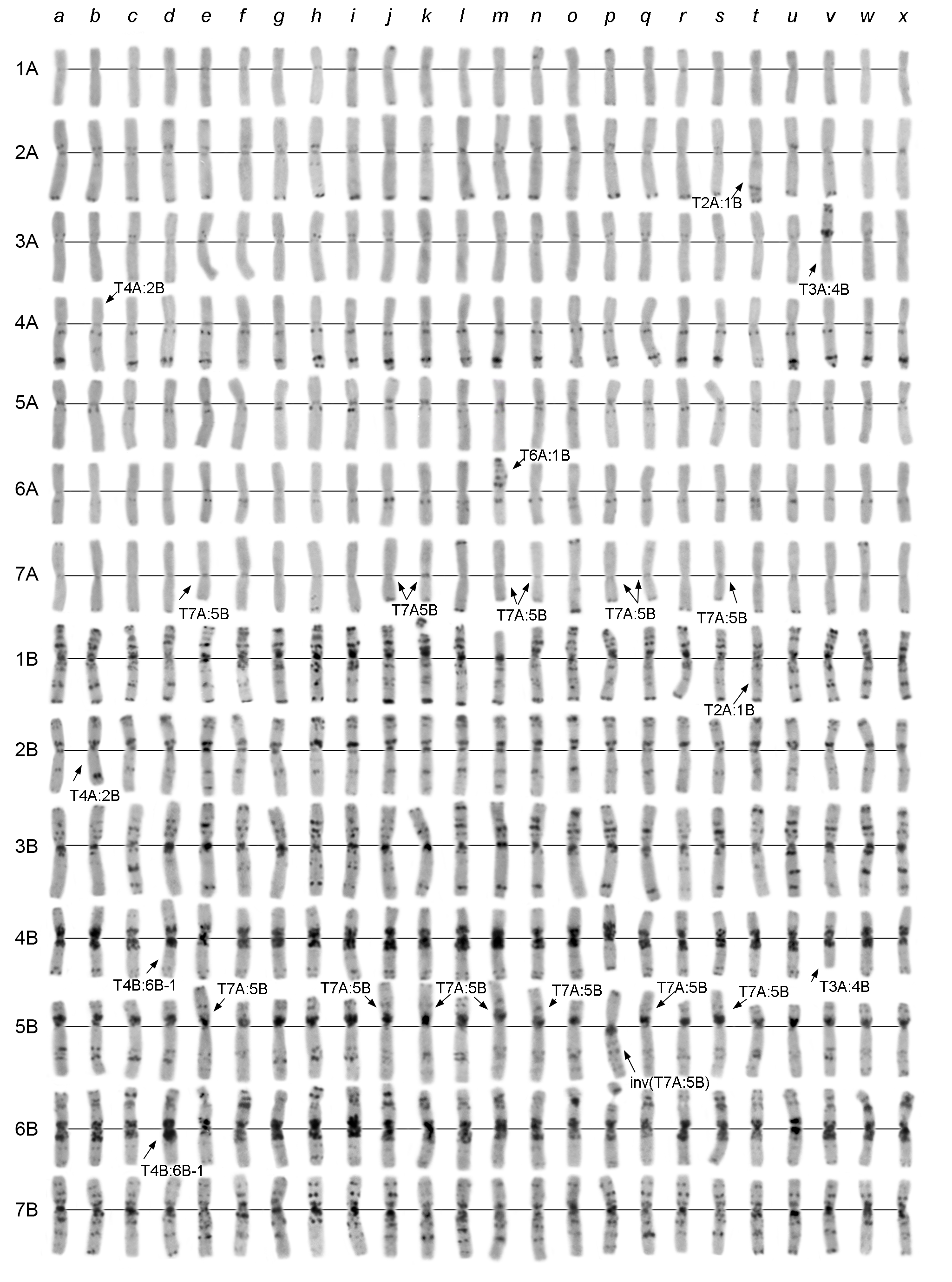

Supplement: S3 Fig — a—PI 275999, b—PI 276013b, c—k-21177, d—PI 191091, e—k-20541, f—IG 45337, g—PI 278644, h—PI 532322, i—TRI-19294, j—INRA 6807, k—INRA 26648, l—k-21589c, m—INRA 26642, n—k-1730, o—k-21433, p—k-12946, q—PI 355467, r—PI 323435, s—INRA 27097, t—INRA 27088, u—INRA 27096, v—k-21419, w—IG 88757, x—IG 94682. 1A–7B —chromosomes. Chromosomal rearrangements are indicated with arrows and designated according to S3 and S4 Tables. (TIF) [file pone.0128556.s003.tif]

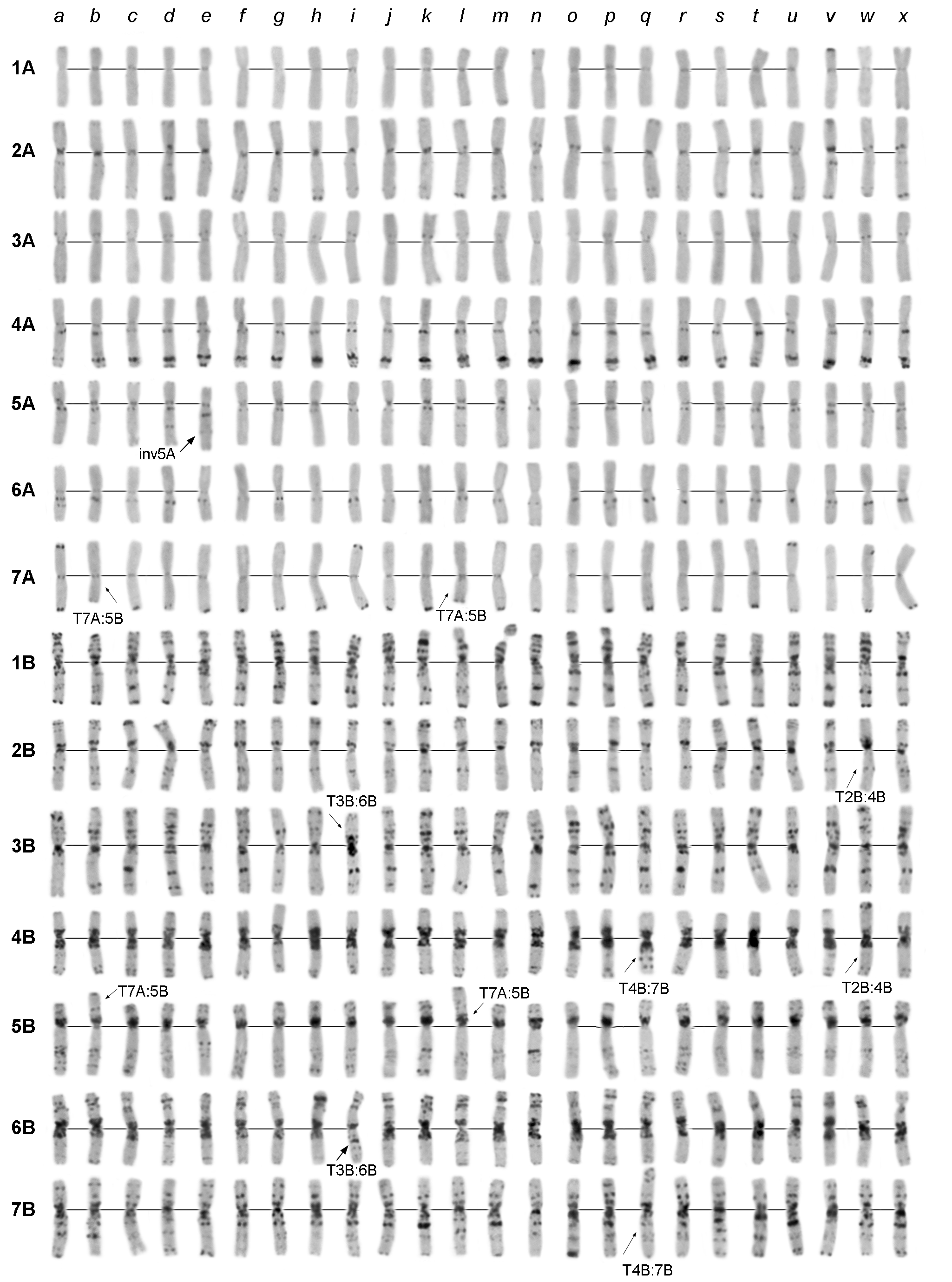

Supplement: S4 Fig — a—PI 355507, b—TRI 584, c—IG 45336, d—INRA 27113, e—TA 10480, f—k-13648, g—k-14039, h—k-38152, i—TRI 16608, j—PI 624908, k—TA 10504, l—k-7146, m—k-45542a, n—k-51768, o—IG 45318a, p—IG 88750, q—IG 45318b, r—k-44167, s—k-44154, t—k-46482, u—k-45514c, v—INRA 23799, w—PI 79899, x—KU-112. 1A–7B —chromosomes. Chromosomal rearrangements are indicated with arrows and designated according to S3 and S4 Tables. (TIF) [file pone.0128556.s004.tif]

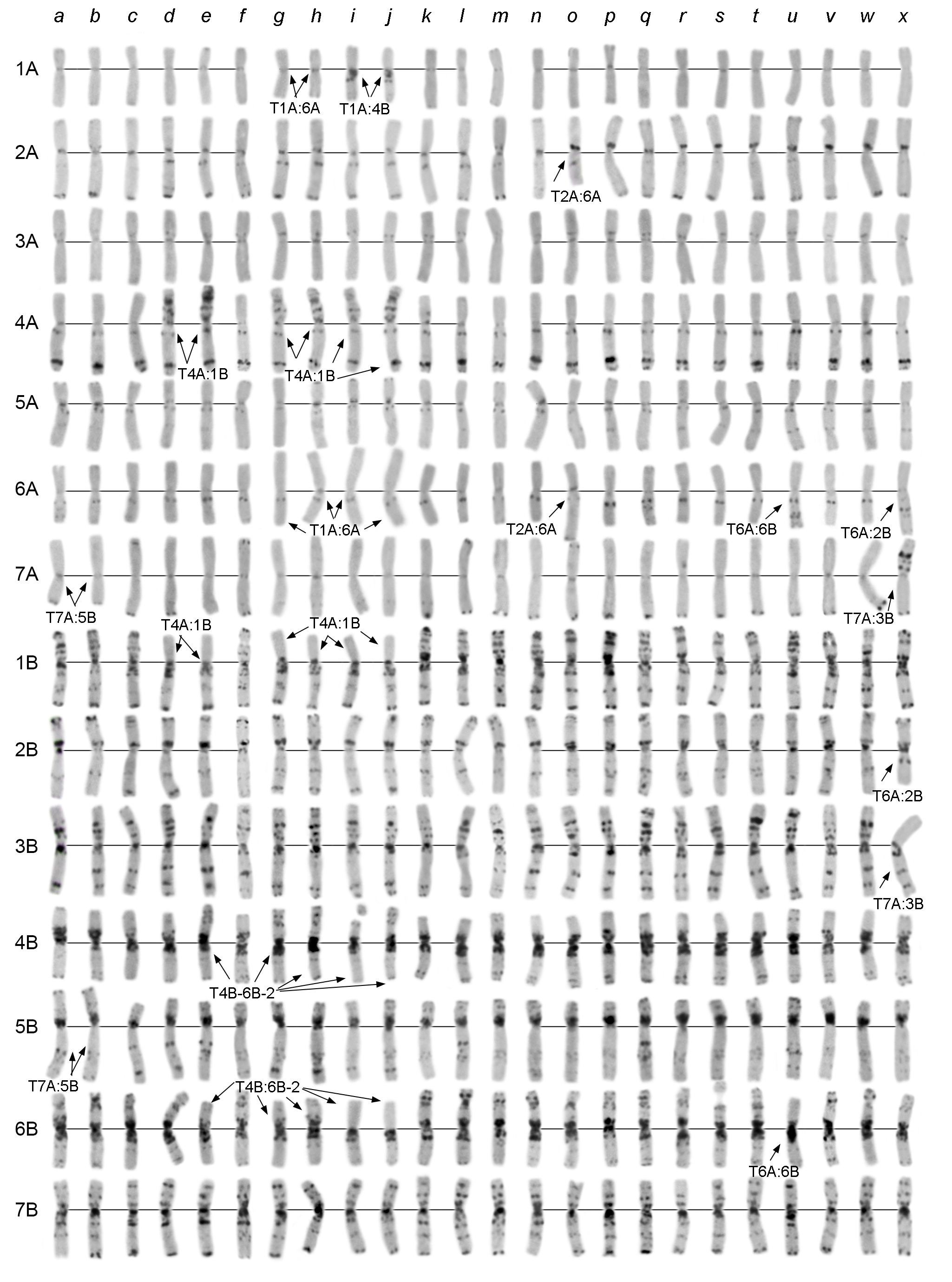

Supplement: S5 Fig — a—INRA 26893, b—INRA 26894, c—INRA 26895, d—INRA 26896, e—INRA 26897, f—INRA 27898, g—IG 127703, h—k-15837, i—IG 45317, j—k-22246, k—k-15840a, l—k-15840b, m—27970, n—IG 45393, o—INRA 27098, p—INRA 27100, q—k-43771, r—IG 45315, s—IG 45303a, t—IG 45303b, u—INRA 27234, v—k-19256, w—INRA 27087, x—PI 577791. 1A–7B —chromosomes. Chromosomal rearrangements are indicated with arrows and designated according to S3 and S4 Tables. (TIF) [file pone.0128556.s005.tif]

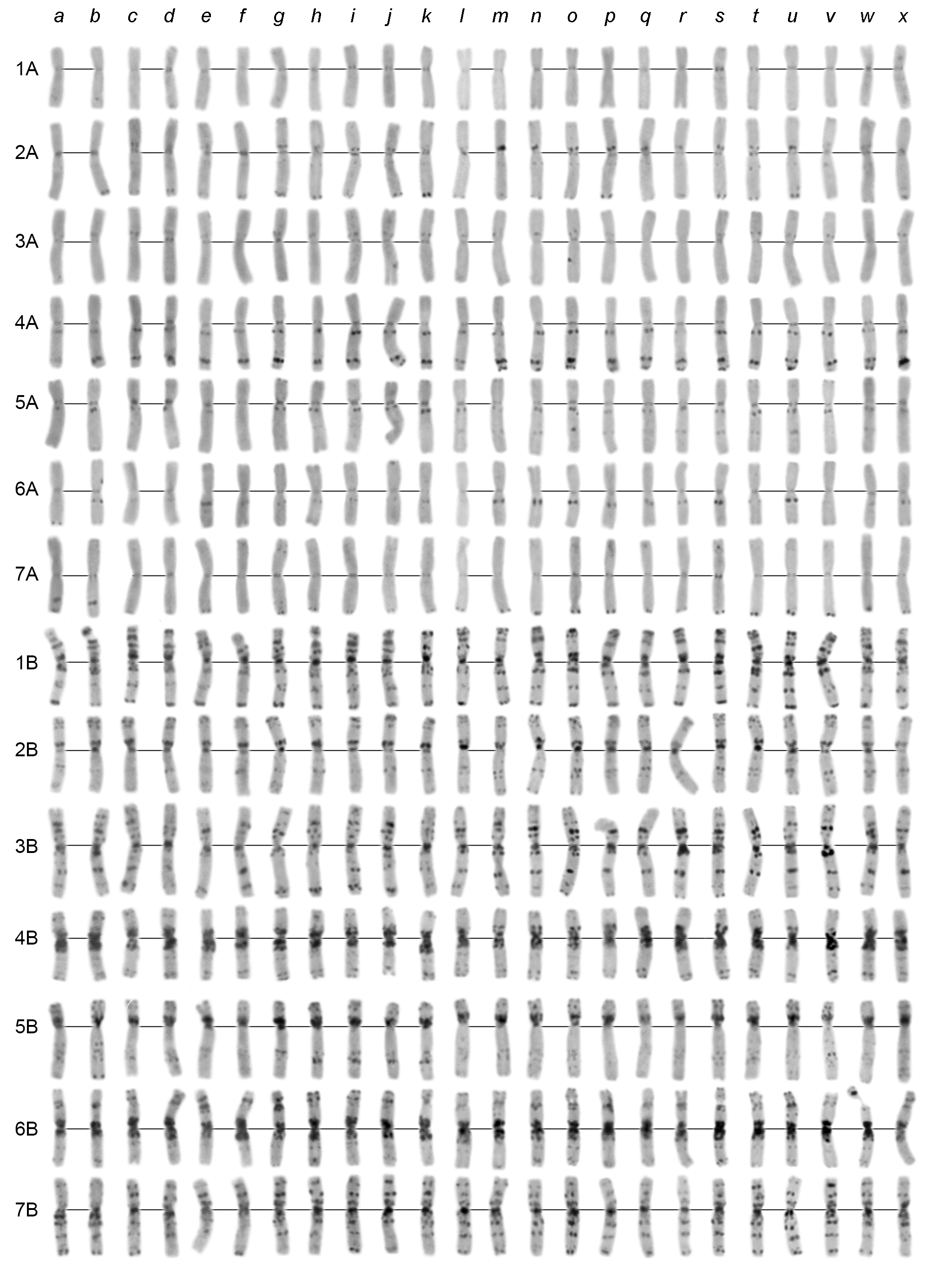

Supplement: S6 Fig — a, b—IG 45254a, b, c—PI 352348, d—PI 355498, e—IG 45363, f—IG 45444, g—TRI 3424, h—TRI 16880, i—PI 352367, j—PI 355496, k—TRI 16879, l—INRA 27085, m—PI 94664, n——IG 45091, o—IG 45069, p—IG 45070, q—IG 45073, r—IG 45068, s—PI 532305, t—TA 10514, u—TRI 28027a, v—TRI 28072a, w—IG 99244, x—k-25459. 1A–7B —chromosomes. Chromosomal rearrangements are indicated with arrows and designated according to S3 and S4 Tables. (TIF) [file pone.0128556.s006.tif]

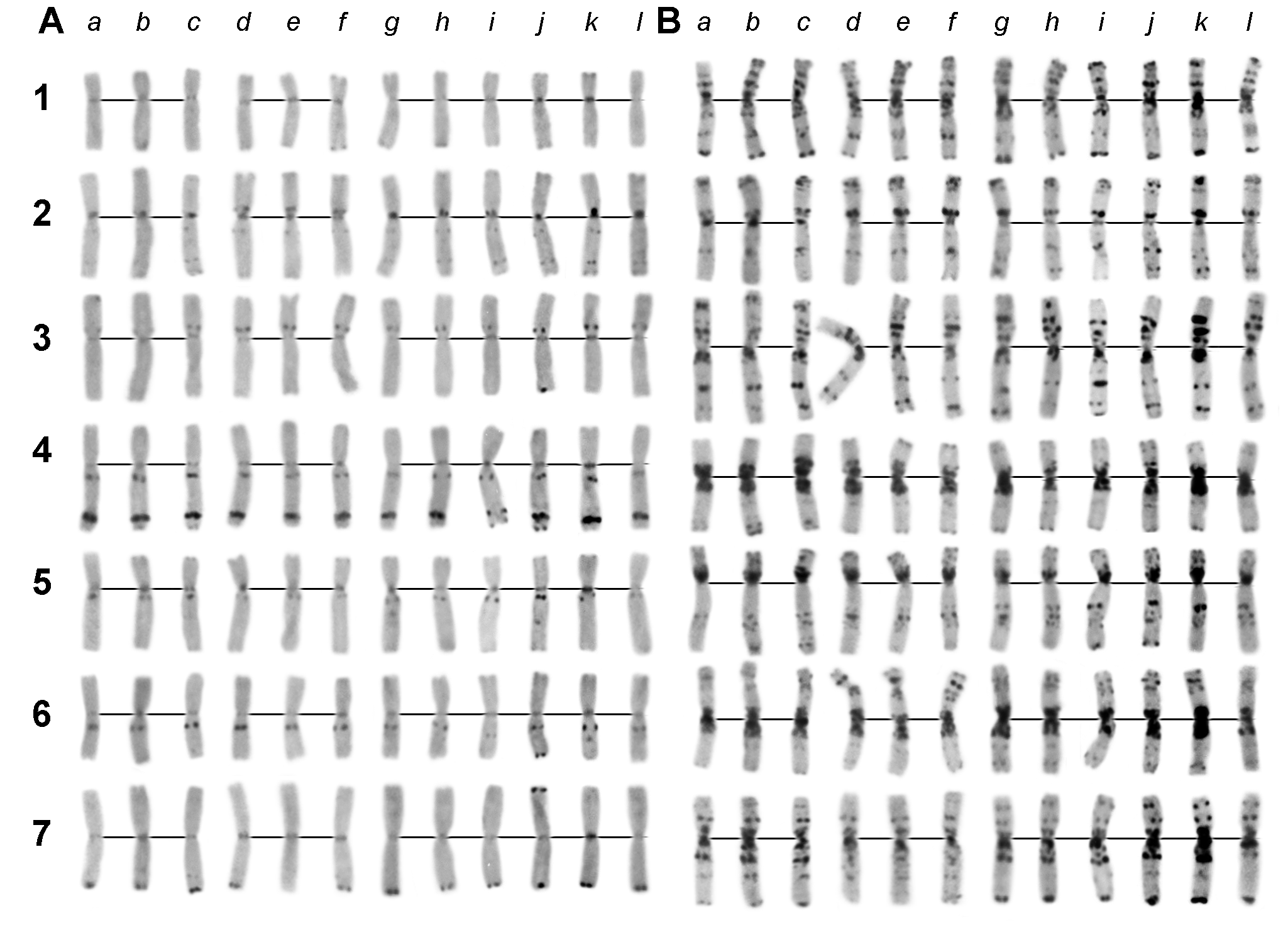

Supplement: S7 Fig — a—TRI 6177, b—TRI 7260, c—TRI 7117, d—TRI 4568, e—TRI 7496, f—TRI 4607, g—PI 470897 (Algeria), h—‘Krasnokutka-10’ (k-62422, Russia), i and j—landraces from Egypt, k—‘Chakinskaya-226’ (k-39099, Russia), l—‘Valentina’ (k-62650, Russia). (TIF) [file pone.0128556.s007.tif]

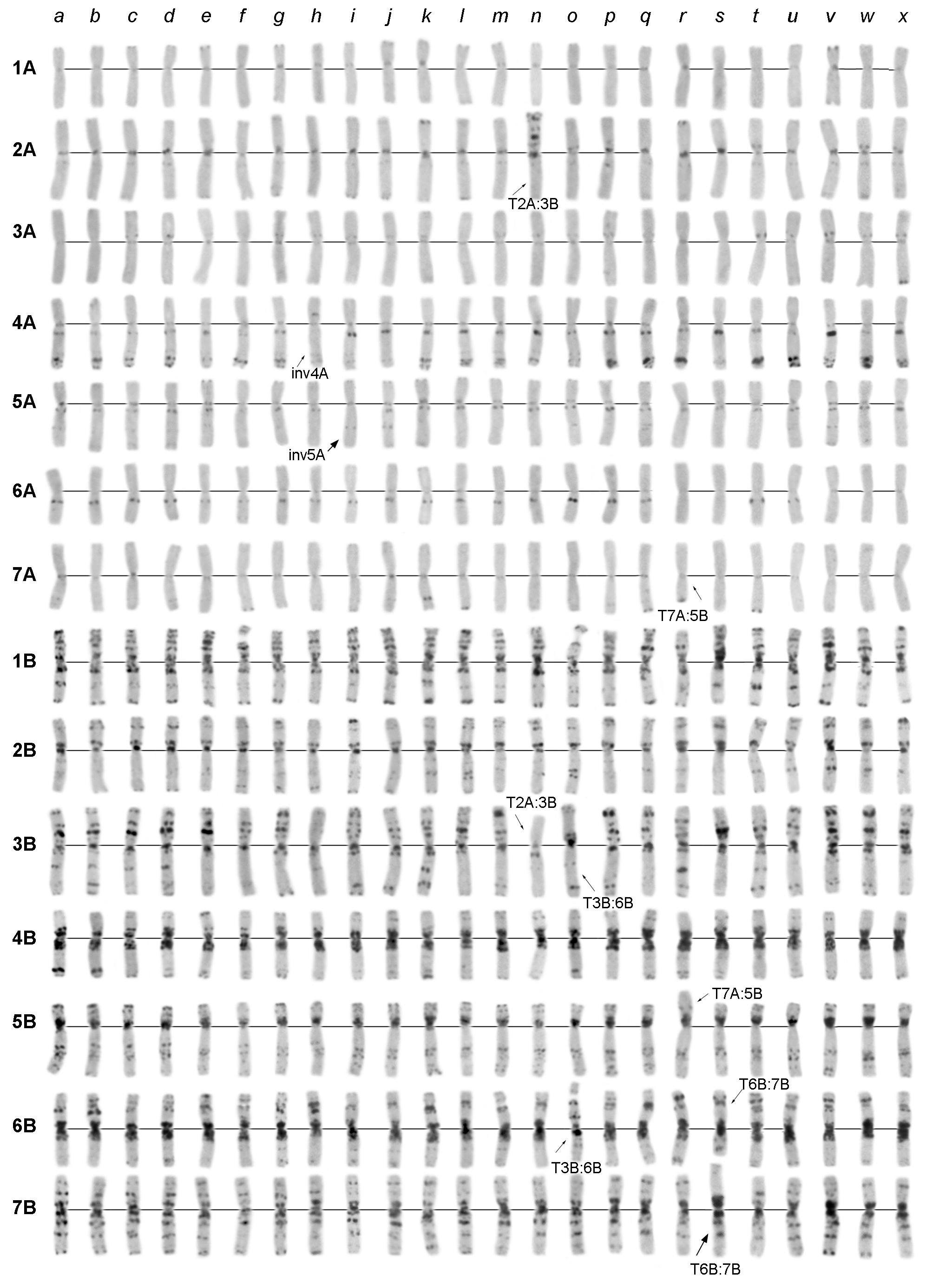

Supplement: S8 Fig — a—KU-1972b, b—KU-1991, c—KU-1952, d—KU-1955 (Kahramanmaraş), d—IG 116171, e—IG 11671, f—IG 116174, g—IG 116181, h—IG 116179b (Gaziantep), i—IG 46149, j—IG 46183, k—IG 46185, l—IG 46171 (Şanlıurfa), m—IG 46250, n—PI 428051, o—PI 428063, p—PI 428045 (Diyarbakır), q—PI 428145 (Mardin), r—k-46632, s—IG 109085, t—IG 131232, u—IG 109085, v—KU-8942, w—IG 113301, x—IG 113302. 1A–7B —chromosomes. Chromosomal rearrangements are indicated with arrows and designated according to S5 Table. (TIF) [file pone.0128556.s008.tif]

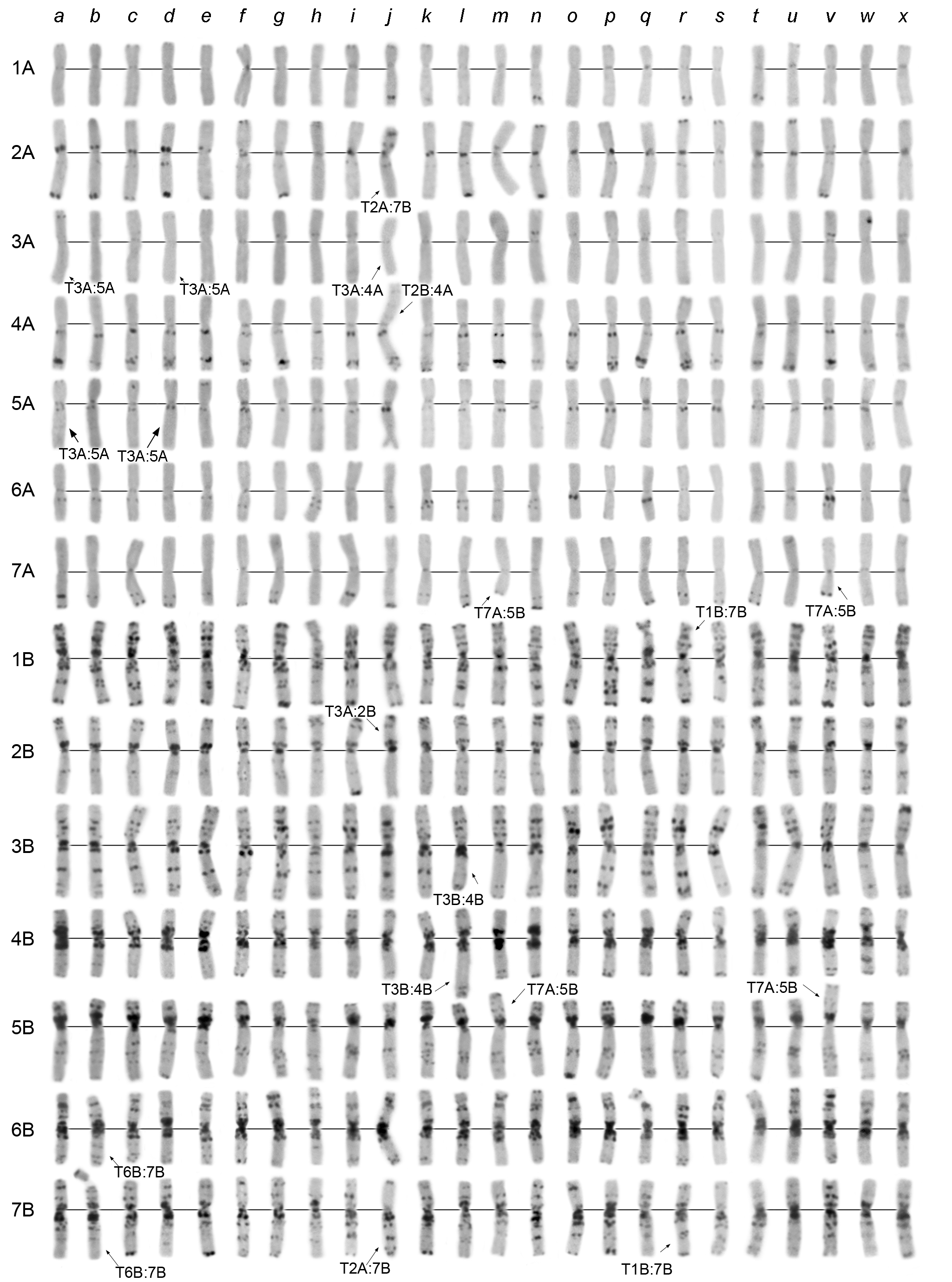

Supplement: S9 Fig — a—UH-J4-2, b—UH-J5-1, c—PI 467019, d—UH-G1-6, e—PI 467014, f—PI 414719, g—TA1057, h—UH-H-8-1, i—UH-NM6, j—PI 538699, k—IG 46531a, l—IG 46531b, m—PI355455, n—IG 46526a, o—IG 115808a, p—IG 139130, q—IG 46486a, r—IG 139129, s—IG 115807, t—IG 119450, u—IG 119408, v—k-17157, w—IG 45506, x—IG 117894. 1A–7B —chromosomes. Chromosomal rearrangements are indicated with arrows and designated according to S5 Table. (TIF) [file pone.0128556.s009.tif]

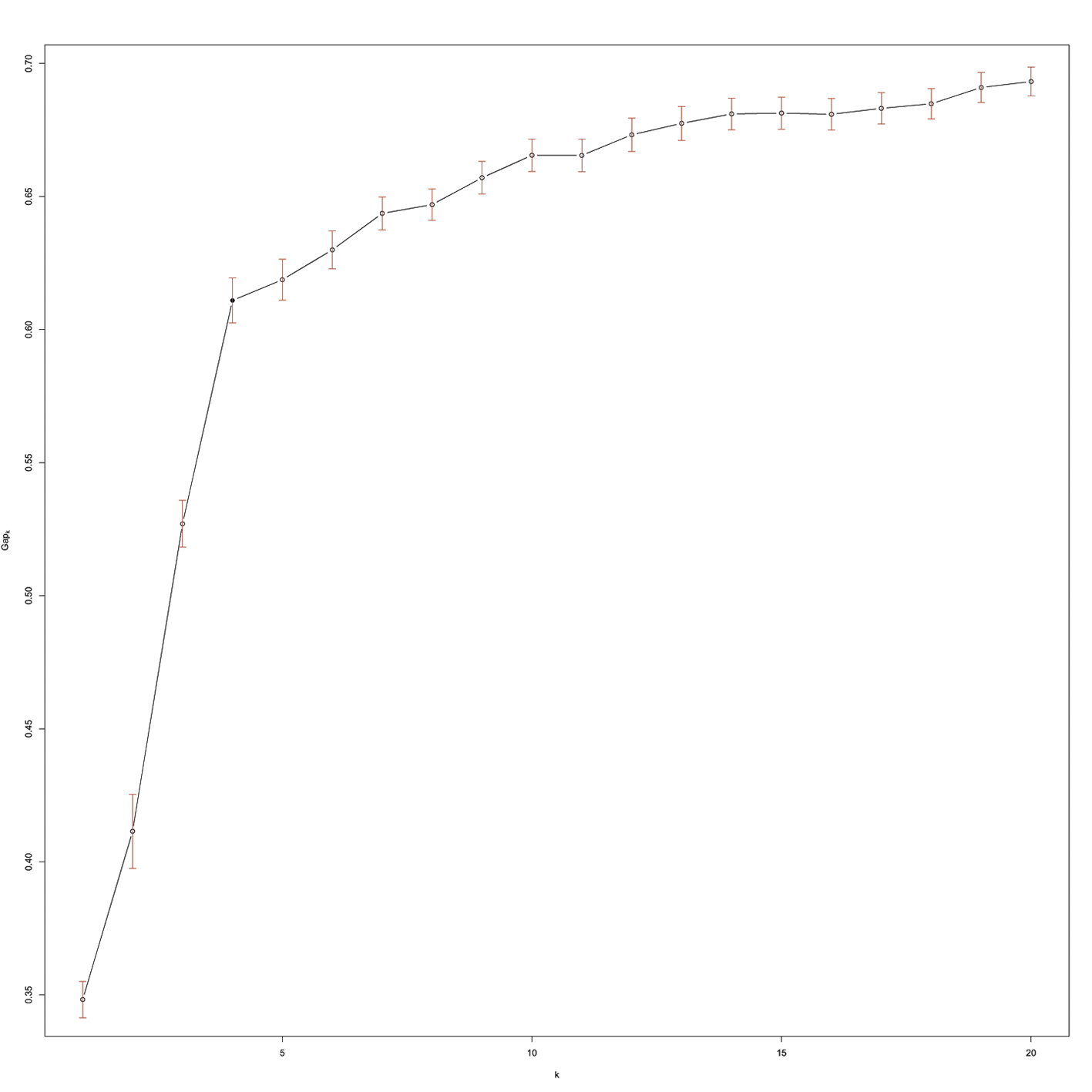

Supplement: S10 Fig — Four main clusters (black filled circle) were identified using gap statistic and the criteria of Tibshirani [43] with twice standard error (cf. material and methods section). (TIF) [file pone.0128556.s010.tif]

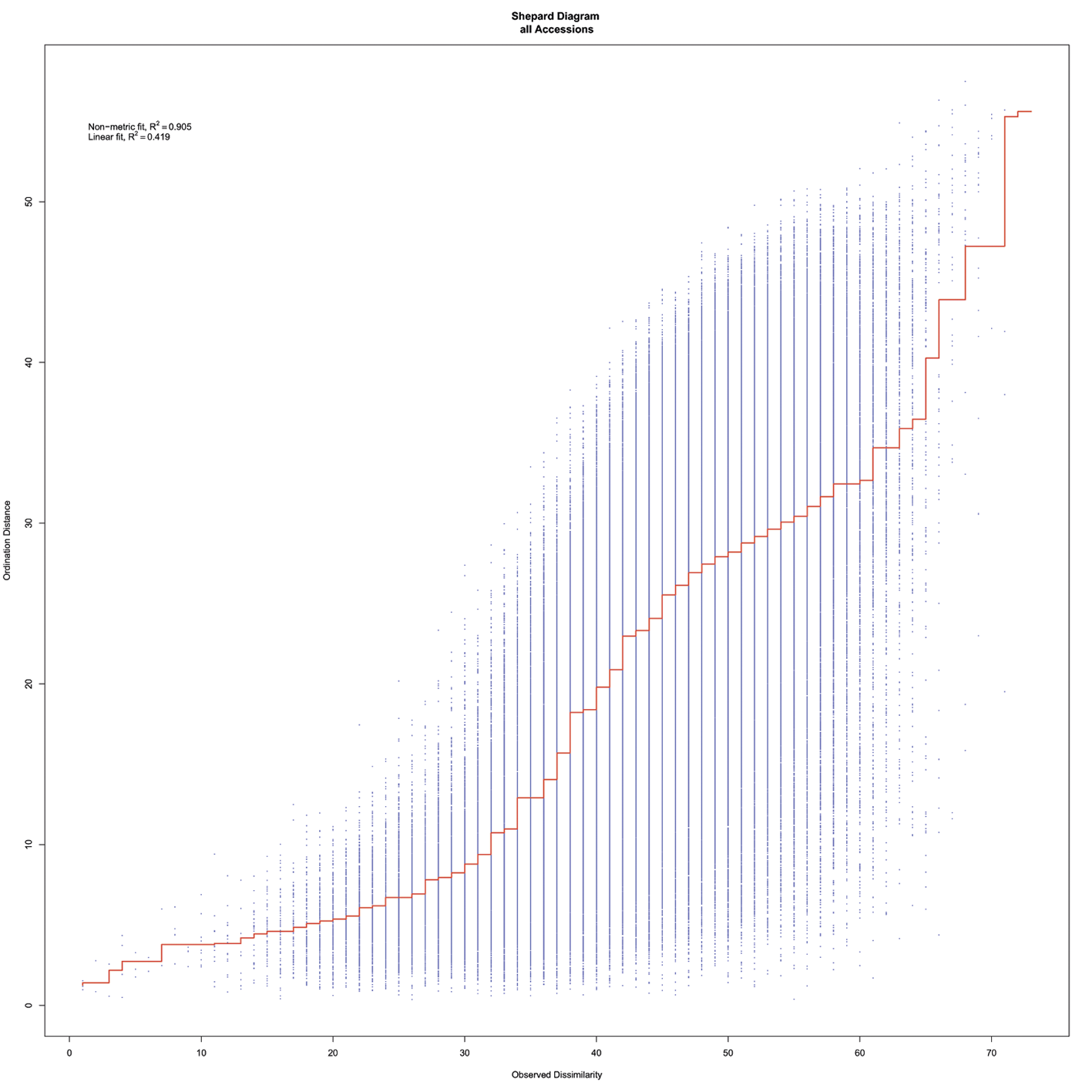

Supplement: S11 Fig — (TIF) [file pone.0128556.s011.tif]

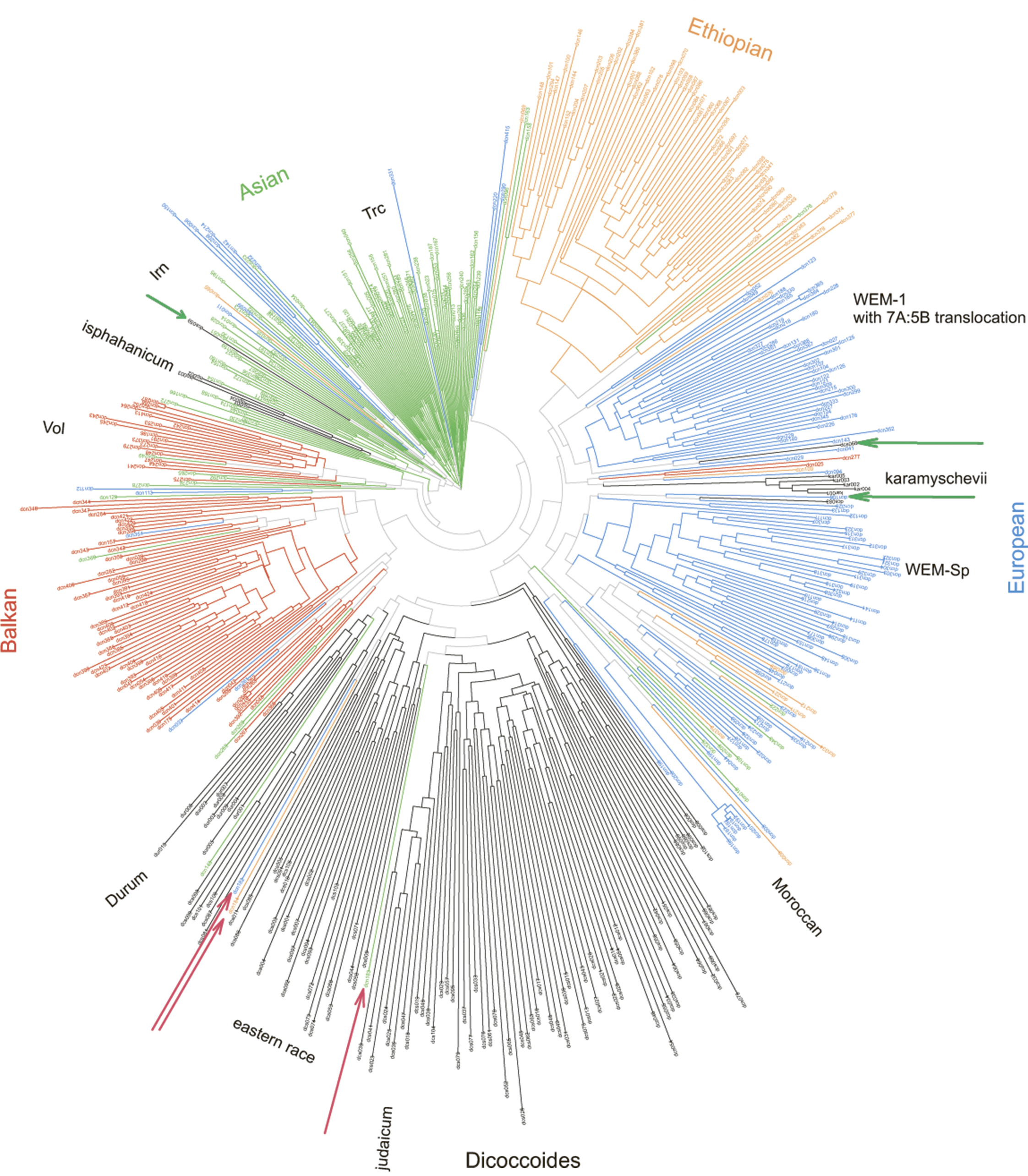

Supplement: S12 Fig — The NJ-tree is based on the C-banding patterns (S2 Table). The four colors blue, red, green and orange are based on k-medoids (Fig 4) and represent the European, Balkan, Asian, and Ethiopian groups, respectively. Additionally, black is used for Dicoccoides and other taxa. Edges are colored with the unique color of all leaves in the respective subtree or otherwise grey. The line codes are the same as in S1 Table. Groups of accessions are named according to the origin of the material: Dicoccoides: western/southern race, ssp. judaicum, eastern/northern race, durum wheat, Mor emmer; Ethiopian group; European group, including Wem-1, Wem-2, WEM-Sp types and T. karamyschevii; Balkan group, including Bal and Vol types; Asian-group, including Irn and Trc types and T. ispahanicum). Lines of T. dicoccoides which clustered within domesticated lines are indicated with green arrows; T. dicoccon lines which clustered within wild emmer are indicated with red arrows. (TIF) [file pone.0128556.s012.tif]
