# Supplementary material for: Chromosomal Passports Provide New Insights into Diffusion of Emmer Wheat
Source: PLoS One. 2015 May 29;10(5):e0128556. doi: 10.1371/journal.pone.0128556 (PMC4449015; doi:10.1371/journal.pone.0128556)
Supplement: S1 File — (DOCX) [file pone.0128556.s013.docx]

**S1 File. Germplasm**

A major problem that we uncovered in our study was connected with the authenticity of the materials as all samples came from *ex situ* repositories. We cannot be sure in every individual case that the sample’s origin corresponds to the information available. Some lines studied here were probably taxonomically misclassified, contaminated or simply mixed up over the course of seed propagation and maintenance *ex situ*. For example, some accessions assigned as *T. dicoccon* had naked seeds. Based on C-banding we had to classify these as T*. durum, T. aethiopicum* Jakubz., or hexaploid *T. aestivum* L. or *T. compactum* Host. Several accessions contained a seed mix of wild and domestic emmer genotypes. Some hulled accessions proved to be diploid, or even belonged to *T. timopheevii* Zhuk. All these accessions were excluded from our study.

Unfortunately in the case of early collections, the original collecting locations are often lost, or lines that were exchanged among genebanks lost their original authenticity. For instance, the accession PI 41024 from Samara was deposited in the USDA National Small Grains Collection in 1915 by Prof. R. Regel (Bureau of Applied Botany, St. Petersburg), and the original line is still maintained in the genebank of VIR (k-417). However, our karyotype analysis showed that these two accessions carry totally different genotypes today; k-417 was similar to other wheats from the same geographic region, whereas PI 41024 was almost identical to emmer lines from Ethiopia. Thus, recent confirmed collections of domesticates and wild forms should be preferred.

Several accessions investigated here were suspect, because they originate from areas (according to genebank information system data) where emmer cultivation has not been reported as a traditional crop in the recent past. So indeed, these accessions could not have been originally collected in these areas. We suspect they were collected elsewhere and then grown at the genebanks in these countries such as Belgium, China, Kuwait, Great Britain, and the Netherlands. Others may have been incorrectly recorded or mixed up during experimental cultivation. Also recent introductions of emmer for experimental work and recent commercialization must be considered.

As recently shown by Jakob et al. [16], gene flow among *ex situ* maintenance, seed contamination or replacement of one line by another as a result of malpractice of personal or other problems will lead to false conclusions regarding the history of the species. We here recommend to consequently remove any doubtful lines from the analysis.

The material that was finally selected for our study was obtained from wheat germplasm collections of **VIR** (All-Russian Research Institute of Plant Industry, St. Petersburg, Russia), 138 accessions; **USDA-ARS** (Aberdeen, Idaho, USA), 123 accessions; **ICARDA** (International Center for Agricultural Research in the Dry Areas, Aleppo, Syria), 96 accessions; **INRA** (Clermont-Ferrand, France), 61 accessions; **IPK** (Leibniz Institute of Plant Genetics and Crop Plant Research, Gatersleben, Germany), 29 accessions, **UH** (Institute of Evolution, University of Haifa, Israel), 22 accessions; **WGRC** (Wheat Genetics Resource Centre, Kansas State University, USA), 6 accessions; and Plant Germplasm Institute, Kyoto University, Kyoto, Japan, 9 accessions. One accession of durum wheat was provided by Dr. E.A. Hussein, Al-Azhar University, Cairo, Egypt, and one accession of *T. karamyschevii* by Dr. I.M. Matitashvili, Institute of Botany, Tbilisi, Georgia. Twelve accessions were represented by duplicates, i.e. the same material was maintained in several genebanks under different accession numbers: IG 127700 = k-7146 (ICARDA and VIR); IG 45483 = TA 10504 (ICARDA and KSU); IG 45428 = PI 286061; IG 45399 = PI 306535; IG 45354 = PI 2789; IG 45311 = PI 94676; IG 88765 = PI 190920; IG 92547 = PI 275998; IG 45398 = PI 277671; IG 45325 = PI 352329a; IG45088 = PI 470738a (all ICARDA and USDA); PI 470737 = TA 2895 (USDA and KSU). In all these cases, only one of the duplicated accessions was considered in the analysis.

Two hundred and twenty (82%) of 269 accessions of domesticated emmer were homogeneous, while the remaining 49 accessions segregated into two (31 accessions), three (10 accessions), four (6 accessions), or five (2 accessions) genotypes that differed from one another in the C-banding patterns and/or in the presence of chromosomal rearrangements. Each cytogenetically distinct genotype was treated as separate entity and designated ‘line’. Altogether our sample included 446 lines of *T. dicoccon* from 47 countries, which were used for evaluating the variability of the C-banding patterns and translocation polymorphism. Only 421 lines were selected for subsequent analysis based on chromosomal passports, whereas 25 lines were excluded for the following reasons:

1) their authenticity is doubted (IG 45337; IG 27125; k-19362; k-44167; k-46482; k-51768; k-275996; PI41024; PI 57536; INRA 27085; k-20368);

2) their collection site information provided is doubted – emmer was not cultivated here in the recent past (IG 45423; PI 352322 (Belgium); k-35890 (Netherlands); PI 278644 (Great Britain); INRA 23799, KU-112, PI 79899 (China); TRI 4342 (Kuwait); (INRA 26648, INRA 26657, INRA 26659 have probably originated from Romania, but not from France; J. David, personal communication);

3) the lines were taxonomically misclassified or represent interspecific hybrids (k-47795; TA 10480).

Ninety-four accessions of wild emmer were cytogenetically homogeneous, five accessions split into two (4 accessions) or three distinct genotypes (1 accession). One of ten *T. durum* accessions consisted of two lines, whereas no segregation was detected in *T. karamyschevii* and *T. ispahanicum* accessions. An overview of the wheat genotypes investigated is presented in Tab. S1.
