# Supplementary material for: Chromosomal Passports Provide New Insights into Diffusion of Emmer Wheat
Source: PLoS One. 2015 May 29;10(5):e0128556. doi: 10.1371/journal.pone.0128556 (PMC4449015; doi:10.1371/journal.pone.0128556)
Supplement: S1 Table — (DOCX) [file pone.0128556.s014.docx]

Table S1 Overview of *T. dicoccon* accessions and their origin

| No | Accession code | Accession number | Chromosomal type | Assignment in k medoid | Country of origin | Collection site, donor, accession name, other information available | Longitude (N) | Latitude (E) | Altitude (m) | Duplicates |
| --- | --- | --- | --- | --- | --- | --- | --- | --- | --- | --- |
|  | Materials studied by C-banding and used in statistical analyses |  |  |  |  |  |  |  |  |  |
|  | dcn001 | IG 45318a^3^ | Eth | 1 | Afghanistan | Unknown (obtained from USDA, PI 94671) | no data | no data | - | - |
|  | dcn002 | IG 45318b^3^ | Trc | 2 | Afghanistan | - | - | - | - | - |
|  | dcn003 | IG 45318c^3^ | Eth | 1 | Afghanistan | - | - | - | - | - |
|  | dcn004 | IG 88750 | Trc | 2 | Afghanistan | unknown (obtained from USDA, PI 94671) | no data | no data | - | - |
|  | dcn005 | TRI 17634 | Trc | 2 | Albania | Tirana | no data | no data | - | - |
|  | dcn006 | INRA 26893 | WEM-1 | 3 | Algeria | unknown | no data | no data | - | - |
|  | dcn007 | INRA 26894 | uncertain | 3 | Algeria | unknown | no data | no data | - | - |
|  | dcn008 | INRA 26895 | Mor | 1 | Algeria | unknown | no data | no data | - | - |
|  | dcn009 | INRA 26896 | Mor | 3 | Algeria | unknown | no data | no data | - | - |
|  | dcn010 | INRA 26897 | Mor | 2 | Algeria | unknown | no data | no data | - | - |
|  | dcn011 | INRA 26898 | Trc | 3 | Algeria | unknown | no data | no data | - | - |
|  | dcn012 | k-13648 | Trc | 2 | Armenia | Echmiadzin, v. Koytul | 40^0^ 20’ | 44^0^ 11’ | - | - |
|  | dcn013 | k-13654 | Trc | 2 | Armenia | Echmiadzin, v. Alikuchak | 40^0^ 32’ | 44^0^ 24’ | - | - |
|  | dcn014 | k-13665 | Trc | 2 | Armenia | Lori, v. Dsekh | 40^0^ 58’ | 44^0^ 39’ | - | - |
|  | dcn015 | k-14039 | Trc | 2 | Armenia | Kamo, v. Shagriz | 40^0^ 34’ | 44^0^ 53’ | - | - |
|  | dcn016 | k-14043 | Trc | 2 | Armenia | Kamo, v. Tzaghkunk | 40^0^ 34’ | 44^0^ 52’ | - | - |
|  | dcn017 | k-14169 | Trc | 2 | Armenia | Yekhegnadzor, v. Solak | 40^0^ 28’ | 44^0^ 42’ | - | - |
|  | dcn018 | k-17560a^2^ | Trc | 2 | Armenia | Goris, v. Agvani | 39^0^ 20’ | 46^0^ 17’ | - | - |
|  | dcn019 | k-17560b^2^ | Trc | 2 | Armenia | - | - | - | - | - |
|  | dcn020 | k-18616 | Trc | 2 | Armenia | Kamo, v. Kishlag (Artsvakar) | 40^0^ 20’ | 45^0^ 09’ | - | - |
|  | dcn021 | k-23634 | Trc | 2 | Armenia | Goris, region v. Tatev | 39^0^ 22’ | 46^0^ 14’ | - | - |
|  | dcn022 | PI 323435 | WEM-2 | 3 | Austria | Vienna | 48^0^ 12‘ | 16^0^ 22’ | 168 | - |
|  | dcn023 | k-16814 | Trc | 2 | Azerbaijan | Zakatala, v. Kashkachay | 41^0^ 23’ | 47^0^ 01’ | - | - |
|  | dcn024 | k-30091 | Trc | 2 | Azerbaijan | Nagorny Karabakh, Stepanakert | 39^0^ 48’ | 46^0^ 44’ | 1596 | - |
|  | dcn025 | k-40170 | Bal | 4 | Azerbaijan | region v. Lachin | 39^0^ 40’ | 46^0^ 33’ | - | - |
|  | dcn026 | k-18774a^2^ | Trc | 2 | Belarus | Mahilyowskaya prov., v. Zapol’ye | 53^0^ 52’ | 29^0^ 44’ | - | - |
|  | dcn027 | k-18774b^2^ | WEM-1 | 3 | Belarus | - | - | - | - | - |
|  | dcn028 | k-39300-1^4^ | hybTrc_Bal | 2 | Belarus | Mahilyowskaya prov., v. Gorki | 54^0^ 17’ | 30^0^ 59’ | - | - |
|  | dcn029 | k-39300-2^4^ | hybTrc_WEM | 3 | Belarus | - | - | - | - | - |
|  | dcn030 | k-39300-3,4,6^4^ | Bal | 4 | Belarus | - | - | - | - | - |
|  | dcn031 | k-39300-5^4^ | Trc | 2 | Belarus | - | - | - | - | - |
|  | dcn032 | IG 45332 | Trc | 2 | Bulgaria | unknown (obtained from USDA, PI 94654) | no data | no data | - | - |
|  | dcn033 | INRA 27089 | Bal | 3 | Bulgaria | unknown | no data | no data | - | - |
|  | dcn034 | INRA 27090 | Trc | 2 | Bulgaria | unknown | no data | no data | - | - |
|  | dcn035 | k-12133 | Trc | 2 | Bulgaria | ‘Limets chernoklasa’, Kraskovo, v. Limets | 41^0^ 22’ | 25^0^ 37’ | - | - |
|  | dcn036 | k-12134a^2^ | Trc | 2 | Bulgaria | ‘Limets beloklasa’, Kraskovo, v. Limets | 41^0^ 22’ | 25^0^ 37’ | - | - |
|  | dcn037 | k-12134b^2^ | Trc | 2 | Bulgaria | - | - | - | - | - |
|  | dcn038 | k-12136 | Trc | 2 | Bulgaria | ‘Limets chernoklasa’, Lovech, v. Yablanitse | 42^0^ 58’ | 24^0^ 03’ | - | - |
|  | dcn039 | k-14236 | Bal | 4 | Bulgaria | ‘Limets’, v. Bela Rada | 43^0^ 58’ | 22^0^ 44’ | - | - |
|  | dcn040 | k-35926 | Trc | 2 | Bulgaria | unknown | no data | no data | - | - |
|  | dcn041 | PI 295065 | WEM-1 | 3 | Bulgaria | unknown, ‘Schwarzer BartWEM-Spelz' | no data | no data | - | - |
|  | dcn042 | PI 94655 | Bal | 4 | Bulgaria | Unknown | no data | no data | - | - |
|  | dcn043 | PI 94659 | Trc | 2 | Bulgaria | Unknown | no data | no data | - | - |
|  | dcn044 | PI 352369 | hybWEM_Dur | 3 | Czech Republic | Central Bohemia | 49^0^ 53’ | 14^0^ 56’ | 393 | - |
|  | dcn045 | PI 355490 | hybTrc_WEM | 3 | Czech Republic | Central Bohemia | 49^0^ 53’ | 14^0^ 56’ | 393 | - |
|  | dcn046 | TRI 9867 | Irn | 2 | Czech Republic | from Botanical garden Brno | 49^0^ 12’ | 16°04' | 227 | - |
|  | dcn047 | TRI 9868 | Bal | 4 | Czech Republic | from Botanical garden Brno | 49^0^ 12’ | 16°04' | 227 | - |
|  | dcn048 | IG 88738 | Bal | 4 | Czechoslovakia | unknown (obtained from USDA, PI 94650) | no data | no data | - | - |
|  | dcn049 | INRA 26651 | hybTrc_WEM | 3 | Czechoslovakia | unknown | no data | no data | - | - |
|  | dcn050 | k-29604 | WEM-2 | 3 | Czechoslovakia | unknown | no data | no data | - | - |
|  | dcn051 | k-29606-1^3^ | Eth | 2 | Czechoslovakia | unknown | no data | no data | - | - |
|  | dcn052 | k-29606-2^3^ | uncertain | 3 | Czechoslovakia | - | - | - | - | - |
|  | dcn053 | k-29606-3,4,5,6^3^ | Trc | 2 | Czechoslovakia | - | - | - | - | - |
|  | dcn054 | TRI 10324 | Bal | 4 | Slovakia | Stará Huta near ZVolen, Central Slovakia | 48° 09’ | 17° 6' 35" | - | - |
|  | dcn055 | IG 45280 | Bal | 4 | Slovakia | W Slovakia, Zapadoslovensky, Biele Karpaty region, Sobotiste | 48° 44'" | 17° 24' | 351 | - |
|  | dcn056 | TRI 27970 | WEM-2 | 3 | Egypt | unknown, ‘Ägyptischer Emmer’ | no data | no data | - | - |
|  | dcn057 | IG 45393 | Trc | 2 | Eritrea | Hamasen. Ukero S of Asmara | 15° 00’ | 38°56' | 2141 | - |
|  | dcn058 | IG 45123 | Eth | 1 | Ethiopia | Shewa | 9° 07' | 38° 23' | 2620 | - |
|  | dcn059 | IG 45124 | Eth | 1 | Ethiopia | Shewa | 9° 07' | 38° 23' | 2620 | - |
|  | dcn060 | IG 45303a^2^ | Eth | 1 | Ethiopia | Gamo Gofa, Chencha | 6° 15' | 37° 34' | 2760 | - |
|  | dcn061 | IG 45303b^2^ | Eth | 1 | Ethiopia | - | - | - | - | - |
|  | dcn062 | IG 45307 | Eth | 1 | Ethiopia | Shewa, 241 km NE of Jima | 8° 41' | 38° 14' | 2113 | - |
|  | dcn063 | IG 45315 | Eth | 1 | Ethiopia | Harerge, Harer market | 9° 20' | 42° 10' | 1527 | - |
|  | dcn064 | IG 45395 | Trc | 2 | Ethiopia | Shewa. Addis Ababa market | 9° 03' | 38° 42' | 2651 | - |
|  | dcn065 | IG 45453 | Trc | 2 | Ethiopia | unknown | no data | no data | 2440 | - |
|  | dcn066 | IG 88877 | Eth | 1 | Ethiopia | Shewa, Modjo | 8° 36' | 39° 07' | 1792 | - |
|  | dcn067 | IG 91674a^2^ | Eth | 1 | Ethiopia | unknown | 9° 12' | 38° 36' | 2440 | - |
|  | dcn068 | IG 91674b^2^ | Eth | 1 | Ethiopia | - | - | - | - | - |
|  | dcn069 | INRA 27087 | hybTrc_Eth | 1 | Ethiopia | unknown | no data | no data | - | - |
|  | dcn070 | INRA 27098 | Eth | 1 | Ethiopia | unknown | no data | no data | - | - |
|  | dcn071 | INRA 27099 | Eth | 1 | Ethiopia | unknown | no data | no data | - | - |
|  | dcn072 | INRA 27100 | Eth | 1 | Ethiopia | unknown | no data | no data | - | - |
|  | dcn073 | INRA 27101 | hybTrc_Eth | 1 | Ethiopia | unknown | no data | no data | - | - |
|  | dcn074 | INRA 27110 | Eth | 1 | Ethiopia | unknown | no data | no data | - | - |
|  | dcn075 | INRA 27115 | Eth | 1 | Ethiopia | unknown | no data | no data | - | - |
|  | dcn076 | INRA 27122 | hybTrc_WEM | 1 | Ethiopia | unknown | no data | no data | - | - |
|  | dcn077 | INRA 27128 | Eth | 1 | Ethiopia | unknown | no data | no data | - | - |
|  | dcn078 | INRA 27129 | Eth | 1 | Ethiopia | unknown | no data | no data | - | - |
|  | dcn079 | INRA 27130 | Eth | 1 | Ethiopia | unknown | no data | no data | - | - |
|  | dcn080 | INRA 27131 | Eth | 1 | Ethiopia | unknown | no data | no data | - | - |
|  | dcn081 | INRA 27133 | Eth | 1 | Ethiopia | unknown | no data | no data | - | - |
|  | dcn082 | INRA 27135 | Eth | 1 | Ethiopia | unknown | no data | no data | - | - |
|  | dcn083 | INRA 27136 | Eth | 1 | Ethiopia | unknown | no data | no data | - | - |
|  | dcn084 | INRA 27137 | Eth | 1 | Ethiopia | unknown | no data | no data | - | - |
|  | dcn085 | INRA 27138 | Trc | 1 | Ethiopia | unknown | no data | no data | - | - |
|  | dcn086 | INRA 27139 | Eth | 1 | Ethiopia | unknown | no data | no data | - | - |
|  | dcn087 | INRA 27140 | Eth | 1 | Ethiopia | unknown | no data | no data | - | - |
|  | dcn088 | INRA 27141 | Eth | 1 | Ethiopia | unknown | no data | no data | - | - |
|  | dcn089 | INRA 27204 | Eth | 1 | Ethiopia | unknown | no data | no data | - | - |
|  | dcn090 | INRA 27205 | Eth | 1 | Ethiopia | unknown | no data | no data | - | - |
|  | dcn091 | INRA 27206 | Eth | 1 | Ethiopia | unknown | no data | no data | - | - |
|  | dcn092 | INRA 27208 | Eth | 1 | Ethiopia | unknown | no data | no data | - | - |
|  | dcn093 | INRA 27220 | Eth | 1 | Ethiopia | unknown | no data | no data | - | - |
|  | dcn094 | INRA 27221 | WEM-2 | 3 | Ethiopia | unknown | no data | no data | - | - |
|  | dcn095 | INRA 27234 | Eth | 1 | Ethiopia | unknown | no data | no data | - | - |
|  | dcn096 | k-13893 | hybTrc_Eth | 2 | Ethiopia | 2-days journey S of Dessie, Oromia prov. | 9°1'48" | 39^0^ 00’ | - | - |
|  | dcn097 | k-19256 | Eth | 1 | Ethiopia | Addis Ababa | 09^0^ 01’ | 38^0^ 44’ | - | - |
|  | dcn098 | k-19285 | Eth | 1 | Ethiopia | 100 km S from Addis Ababa, Oromia prov. | 08^0^ 15’ | 38^0^ 30’ | 2400 | - |
|  | dcn099 | k-24397 | Trc | 3 | Ethiopia | region Yubdo | 08^0^ 56’ | 35^0^ 26’ | - | - |
|  | dcn100 | k-43771 | Eth | 1 | Ethiopia | Addis Ababa, market; Oromia prov. | 09^0^ 01’ | 38^0^ 44’ | - | - |
|  | dcn101 | k-5154 | Eth | 1 | Ethiopia | unknown, ‘Ajar’ | 9°1'48" | 38° 44' 24" | - | - |
|  | dcn102 | KU-9028 | Eth | 1 | Ethiopia | 10 km from Dessie to Haik | 11°8'44" | 39° 38' | 2500 | - |
|  | dcn103 | PI 577791 | Eth | 1 | Ethiopia | Shewa | 9° 07' | 38° 23' | 2620 | - |
|  | dcn104 | k-21582 | WEM-1 | 3 | France | unknown | no data | no data | - | - |
|  | dcn105 | INRA 2779 | hybWEM_Dur | 2 | France | unknown | no data | no data | - | - |
|  | dcn106 | INRA 6807 | WEM-2 | 3 | France | unknown | no data | no data | - | - |
|  | dcn107 | INRA 898 | WEM-1 | 3 | France | unknown | no data | no data | - | - |
|  | dcn108 | INRA 899 | WEM-2 | 3 | France | unknown | no data | no data | - | - |
|  | dcn109 | INRA 900 | WEM-2 | 1 | France | unknown | no data | no data | - | - |
|  | dcn110 | k-21588 | WEM-1 | 3 | France | unknown | no data | no data | - | - |
|  | dcn111 | k-21589a^3^ | WEM-1 | 3 | France | unknown | no data | no data | - | - |
|  | dcn112 | k-21589b^3^ | hybWEM_Bal | 3 | France | - | - | - | - | - |
|  | dcn113 | k-21589c^3^ | hybWEM_Bal | 3 | France | - | - | - | - | - |
|  | dcn114 | IG 45439 | WEM-1 | 3 | Georgia | Tbilisi, collected by Dekaprelevich L. | 41° 43' | 44° 47' | 642 | - |
|  | dcn116 | k-14934 | Trc | 2 | Georgia | near v. Bakuri, Khashuri | 41^0^ 44’ | 43^0^ 31’ | - | - |
|  | dcn117 | k-14937 | Trc | 2 | Georgia | South Ossetia, region Tskhinvali | 42^0^ 13’ | 43^0^ 57’ | - | - |
|  | dcn118 | k-27885 | Trc | 2 | Georgia | South Ossetia, v. Sokhta (Tokhta) | 42^0^ 18’ | 44^0^ 33’ | - | - |
|  | dcn119 | k-27887 | Trc | 2 | Georgia | South Ossetia, region Tskhinvali | 42^0^ 13’ | 43^0^ 57’ | - | - |
|  | dcn120 | TRI 16608 | Trc | 2 | Georgia | Didi Gomareti, Rayon Dmanisi (Lower Kartli), on the Calka Dmanisi Plateau, Georgian village | 41° 43' | 44° 47' | - | - |
|  | dcn121 | INRA 26639 | WEM-1 | 3 | Germany | unknown | no data | no data | - | - |
|  | dcn122 | INRA 26642 | WEM-1 | 3 | Germany | unknown | no data | no data | - | - |
|  | dcn123 | INRA 27123 | hybTrc_WEM | 3 | Germany | unknown | no data | no data | - | - |
|  | dcn124 | INRA 27124 | WEM-1 | 3 | Germany | unknown | no data | no data | - | - |
|  | dcn125 | k-1730 | WEM-1 | 3 | Germany | unknown, ‘Dichter Rötlicher’ | no data | no data | - | - |
|  | dcn126 | k-18623 | WEM-1 | 3 | Germany | Baden-Württemberg, Hohenheim | 48^0^ 42’ | 09^0^ 13’ | - | - |
|  | dcn127 | k-20747 | WEM-2 | 3 | Germany | Erfurt | 50^0^ 59’ | 11^0^ 01’ | - | - |
|  | dcn128 | k-20750 | WEM-2 | 3 | Germany | Hohenheim | 48^0^ 42’ | 09^0^ 13’ | - | - |
|  | dcn129 | k-21433 | Bal | 2 | Germany | Thüringen, t. Erfurt | 50° 59' | 11^0^ 01’ | - | - |
|  | dcn130 | k-21961 | Trc | 2 | Germany | Württembergische Landessaatzuchtanstalt, Hohenheim, Stuttgart | 48° 42' | 09^0^ 13’ | - | - |
|  | dcn131 | k-81 | WEM-1 | 3 | Germany | unknown, ‘Amidonnier Weisser’ | no data | no data | - | - |
|  | dcn132 | TRI 1777a^2^ | WEM-1 | 3 | Germany | ‘Brauner Behaarter Winter-Emmer’ | no data | no data | - | - |
|  | dcn133 | TRI 1777b^2^ | WEM-2 | 3 | Germany | - | - | - | - | - |
|  | dcn134 | IG 45379 | Trc | 2 | Greece | Gretzia (obtained from USDA, PI 94618) | no data | no data | - | - |
|  | dcn135 | IG 45390 | Trc | 2 | Greece | unknown (obtained from USDA, PI 94682) | no data | no data | - | - |
|  | dcn136 | IG 45410 | WEM-2 | 3 | Greece | Gretzia ( obtained from USDA, PI 94620) | no data | no data | 140 | - |
|  | dcn137 | IG 88757 | Trc | 2 | Greece | unknown (obtained from USDA, PI 94682) | no data | no data | - | - |
|  | dcn138 | PI 94682 | Trc | 2 | Greece | Unknown | no data | no data | - | - |
|  | dcn139 | IG 45426 | Trc | 2 | Hungary | Pest | 47° 29' | 19° 04' | - | - |
|  | dcn140 | INRA 26654 | WEM-1 | 3 | Hungary | unknown | no data | no data | - | - |
|  | dcn141 | PI 272527 | WEM-Sp | 3 | Hungary | Pest | 47° 29' | 19° 04' | - | - |
|  | dcn142 | PI 272528 | WEM-1 | 3 | Hungary | Pest | 47° 29' | 19° 04' | - | - |
|  | dcn143 | PI 290517 | WEM-1 | 3 | Hungary | Pest, ‘Schwarzer Bart’ | 47° 29' | 19° 04' | - | - |
|  | dcn144 | k-14928a^2^ | Eth | 1 | India | unknown, ‘Khapli’ | no data | no data | - | - |
|  | dcn145 | k-14928b^2^ | WEM-1 | 3 | India | - | - | - | - | - |
|  | dcn146 | k-19475 | Eth | 1 | India | unknown, ‘Khapli’ | no data | no data | - | - |
|  | dcn147 | k-44154 | Eth | 1 | India | province Andhra Pradesh | 17^0^ 23’ | 78^0^ 28’ | - | - |
|  | dcn148 | k-45514a^3^ | Eth | 1 | India | unknown, ‘NP 200’ | no data | no data | - | - |
|  | dcn149 | k-45514b^3^ | hybEth_Dur | 2 | India | - | - | - | - | - |
|  | dcn150 | k-45514c^3^ | Eth | 3 | India | - | - | - | - | - |
|  | dcn151 | k-46466 | Trc | 2 | India | unknown | no data | no data | - | - |
|  | dcn152 | k-7141 | Eth | 1 | India | unknown | no data | no data | - | - |
|  | dcn153 | k-8572 | WEM-1 | 3 | India | unknown | no data | no data | - | - |
|  | dcn154 | IG88724a^2^ | Irn | 2 | Iran | unknown (obtained from USDA, PI 94624) | no data | no data | - | - |
|  | dcn155 | IG88724^b^ | Trc | 2 | Iran | - | - | - | - | - |
|  | dcn156 | k-14322 | Trc | 2 | Iran | ‘Parvik’, Southern Khorossan, Gonabad region, Dzhumein vil. | 32° 31' 3" | 56^0^ 59’ | 1645 | - |
|  | dcn157 | k-44924 | Bal | 4 | Iran | unknown | no data | no data | - | - |
|  | dcn158 | k-45541 | Irn | 2 | Iran | unknown | no data | no data | - | - |
|  | dcn159 | k-45542a^2^ | Irn | 2 | Iran | unknown | no data | no data | - | - |
|  | dcn160 | k-45542b^2^ | Irn | 2 | Iran | - | - | - | - | - |
|  | dcn161 | k-45543a^3^ | Trc | 2 | Iran | unknown | no data | no data | - | - |
|  | dcn162 | k-45543b^3^ | Trc | 2 | Iran | - | - | - | - | - |
|  | dcn163 | k-45543c^3^ | hybIrn_Dsc | 2 | Iran | - | - | - | - | - |
|  | dcn164 | k-45544 | Irn | 2 | Iran | unknown | no data | no data | - | - |
|  | dcn165 | k-7146 | WEM-1 | 3 | Iran | N Khorossan, Kachkabad vil | 37° 19' 21" | 56° 59' 24" | 1568 | IG127700 |
|  | dcn166 | k-7506 | Irn | 2 | Iran | N Khorossan, Kachkabad vil | 37° 19' 21" | 56° 59' 24" | 1568 | - |
|  | dcn167 | k-7507 | Trc | 2 | Iran | N Khorossan, Kachkabad vil | 37° 19' 21" | 56° 59' 24" | 1568 | - |
|  | dcn168 | PI 347230 | Irn | 2 | Iran | Unknown | no data | no data | - | - |
|  | dcn169 | PI 624903 | Irn | 2 | Iran | Cordestan, Saghez | 36° 14' | 46° 16' | 1458 | - |
|  | dcn170 | PI 624904 | Irn | 2 | Iran | Cordestan, Saghez | 36° 14' | 46° 16' | 1458 | - |
|  | dcn171 | PI 624908 | Irn | 2 | Iran | Cordestan, Saghez | 36° 14' | 46° 16' | 1458 | - |
|  | dcn172 | PI 626391 | Irn | 2 | Iran | Esfahan, Yazd | 32° 41' | 51° 41' | 1573 | - |
|  | dcn173 | PI 626468 | Irn | 2 | Iran | Esfahan, Yazd | 32° 41' | 51° 41' | 1573 | - |
|  | dcn174 | TA 10504 | Irn | 2 | Iran | 5 km before Dandi, after Gorgan village | 36° 32' 10" | 47° 39' 29" | 1776 | IG 45483 |
|  | dcn175 | INRA 27088 | Bal | 4 | Italy | unknown | no data | no data | - | - |
|  | dcn176 | INRA 27097 | WEM-1 | 3 | Italy | unknown | no data | no data | - | - |
|  | dcn177 | k-21307 | WEM-2 | 3 | Italy | ‘Zariola’, v. Pellegrino, Parme | 44^0^ 44’ | 09^0^ 55’ | 410 | - |
|  | dcn178 | k-21309 | WEM-2 | 3 | Italy | v. Pellegrino, Parme | 44^0^ 44’ | 09^0^ 55’ | 410 | - |
|  | dcn179 | k-21310 | WEM-2 | 3 | Italy | v. Colle Paganica | 42^0^ 30’ | 13^0^ 16’ | 775 | - |
|  | dcn180 | k-21416 | WEM-1 | 3 | Italy | v. Colle Paganica | 42^0^ 30’ | 13^0^ 16’ | 775 | - |
|  | dcn181 | k-21419 | WEM-2 | 3 | Italy | v. Colle Paganica | 42^0^ 30’ | 13^0^ 16’ | 775 | - |
|  | dcn182 | IG 45254a^3^ | Dsc | 3 | Jordan | Al Balqa, Al-Za’tary | 32° 04' | 35° 45' 37" | 960 | - |
|  | dcn183 | IG 45254b^3^ | Dsc | 2 | Jordan | - | - | - | - | - |
|  | dcn184 | IG 45254c^3^ | Dsc | 1 | Jordan | - | - | - | - | - |
|  | dcn185 | K-34678 | Trc | 2 | Kazakhstan | region Asubulak | 49^0^ 33’ | 83^0^ 03’ | - | - |
|  | dcn186 | k-46995 | Vol | 4 | Kazakhstan | ‘Polba Kokchetavskaya’, Kokshetau region | 51° 10' | 71° 26' | - | - |
|  | dcn187 | k-19091 | Trc | 2 | Latvia | region v. Pasiene | 56^0^ 17’ | 28^0^ 10’ | - | - |
|  | dcn188 | k-38185-1^4^ | WEM-1 | 3 | Latvia | region v. Pasiene | 56^0^ 17’ | 28^0^ 10’ | - | - |
|  | dcn189 | k-38185-2,3^4^ | Trc | 2 | Latvia | - | - | - | - | - |
|  | dcn190 | k-38185-5^4^ | hybTrc_WEM | 3 | Latvia | - | - | - | - | - |
|  | dcn191 | k-38185-6^4^ | Trc | 2 | Latvia | - | - | - | - | - |
|  | dcn192 | IG 127703 | Mor | 3 | Morocco | Unknown | no data | no data | - | - |
|  | dcn193 | IG 127704 | Mor | 3 | Morocco | Unknown | no data | no data | - | - |
|  | dcn194 | IG 45317 | Mor | 3 | Morocco | Unknown | no data | no data | - | - |
|  | dcn195 | IG 45411 | Trc | 2 | Morocco | Unknown | no data | no data | - | - |
|  | dcn196 | IG 88730 | Mor | 3 | Morocco | Unknown | no data | no data | - | - |
|  | dcn197 | k-15837 | Mor | 3 | Morocco | unknown | no data | no data | - | - |
|  | dcn198 | k-15840a^3^ | Mor | 3 | Morocco | unknown | no data | no data | - | - |
|  | dcn199 | k-15840b^3^ | Mor | 3 | Morocco | - | - | - | - | - |
|  | dcn200 | k-15840c^3^ | hybTrc_Mor | 3 | Morocco | - | - | - | - | - |
|  | dcn201 | k-22246 | Mor | 3 | Morocco | unknown | no data | no data | - | - |
|  | dcn202 | IG 45068 | Eth | 1 | Oman | Salalah | 17° 01' | 54° 06' | 178 | - |
|  | dcn203 | IG 45069 | Eth | 1 | Oman | Salalah | 17° 01' | 54° 06' | 178 | - |
|  | dcn204 | IG 45070 | Eth | 1 | Oman | Al Bāţinah, 30 km W Rustag valley bottom | 23° 24' 58" | 57° 08' | 700 | - |
|  | dcn205 | IG 45073 | Eth | 1 | Oman | Al Kauril | 23° 10' | 56° 31' | 200 | - |
|  | dcn206 | IG 45091 | Eth | 1 | Oman | 2 km NE Al Hamra | 23° 06' 48" | 57° 17' 24" | 500 | - |
|  | dcn207 | PI 532305 | Eth | 1 | Oman | 60 km SW of Sohar, Western Hajar Province. Farm store | 23° 36' 36" | 58° 32' 24" | - | - |
|  | dcn208 | IG 45363 | hybWEM_Dsc | 3 | Palestine | unknown | no data | no data | - | - |
|  | dcn209 | IG 45444 | uncertain | 2 | Palestine | unknown | no data | no data | - | - |
|  | dcn210 | PI 352367 | WEM-2 | 3 | Palestine | Unknown | no data | no data | - | - |
|  | dcn211 | PI 355496 | WEM-2 | 1 | Palestine | Ancient Palestine | no data | no data | - | - |
|  | dcn212 | TRI 16880 | WEM-2 | 1 | Israel | Unknown | no data | no data | - | - |
|  | dcn213 | TRI 16879 | WEM-2 | 3 | Israel | Unknown | no data | no data | - | - |
|  | dcn214 | TRI 3424 | hybWEM_Dsc | 3 | Israel | unknown | no data | no data | - | - |
|  | dcn215 | PI 286061 | WEM-1 | 3 | Poland | Unknown | no data | no data | - | IG 45428 |
|  | dcn216 | PI 191781 | WEM-1 | 3 | Portugal | Unknown | no data | no data | - | - |
|  | dcn217 | k-45863 | hybWEM_Trc | 3 | Romania | unknown | no data | no data | - | - |
|  | dcn218 | k-45926-1^3^ | WEM-1 | 3 | Romania | unknown | no data | no data | - | - |
|  | dcn219 | k-45926-2,3,4,5,6^3^ | WEM-1 | 3 | Romania | - | - | - | - | - |
|  | dcn220 | k-45926-4^3^ | WEM-1 | 3 | Romania | - | - | - | - | - |
|  | dcn221 | PI 306531 | hybWEM_Trc | 1 | Romania | Unknown | no data | no data | - | - |
|  | dcn222 | PI 306533a^2^ | hybWEM_Trc | 2 | Romania | Unknown | no data | no data | - | - |
|  | dcn223 | PI 306533b^2^ | hybWEM_Trc | 3 | Romania | - | - | - | - | - |
|  | dcn224 | PI 306534 | WEM-2 | 3 | Romania | Unknown | no data | no data | - | - |
|  | dcn225 | PI 306535 | Trc | 2 | Romania | Unknown | no data | no data | - | IG 45399 |
|  | dcn226 | PI 306536 | WEM-1 | 3 | Romania | Unknown | no data | no data | - | - |
|  | dcn227 | PI 306537 | WEM-1 | 3 | Romania | Unknown | no data | no data | - | - |
|  | dcn228 | PI 306538 | hybTrc_WEM | 3 | Romania | Unknown | no data | no data | - | - |
|  | dcn229 | PI 362071 | WEM-1 | 3 | Romania | Cluj | 46° 46' | 23° 35' | - | - |
|  | dcn230 | IG 127707 | Vol | 2 | Russia | Saratov, t. Novouzensk | 50° 29' | 48° 09' | 31 | - |
|  | dcn231 | IG 127708 | Trc | 2 | Russia | Yekaterinburg, t. Krasnoufimsk, collective farm | 56° 36' | 57° 45' | 248 | - |
|  | dcn232 | IG 127709a^2^ | hybTrc_WEM | 3 | Russia | Astrakhan, t. Yenotayevka | 47°14' | 47° 01' | -19 | - |
|  | dcn233 | IG 127709^b^ | hybTrc_WEM | 2 | Russia | - | - | - | - | - |
|  | dcn235 | IG 45354 | Vol | 4 | Russia | Yaroslavl Region | 57° 36' | 39° 52' | 100 | PI 2789 |
|  | dcn236 | IG 45355 | Trc | 2 | Russia | Krasnodar, from Stepnaya Experiment Station, State Institute of Experimental Agronomy | 45° 46' 51" | 38° 33' 31" | 4 | - |
|  | dcn237 | IG 45386 | Trc | 2 | Russia | Dagestan | 42° 08' | 47° 5' 41" | 2197 | - |
|  | dcn238 | k-10456 | Trc | 2 | Russia | Tatarstan, t. Chistopol | 55^0^ 21’ | 50^0^ 37’ | - | - |
|  | dcn239 | k-13927 | Trc | 2 | Russia | Dagestan, v. Yersi | 42^0^ 00’ | 48^0^ 01’ | - | - |
|  | dcn240 | k-22481-1^5^ | Trc | 2 | Russia | Chuvashia, v. Raskildino | 55^0^ 41’ | 46^0^ 34’ | - | - |
|  | dcn241 | k-22481-3^5^ | Vol | 4 | Russia | - | - | - | - | - |
|  | dcn242 | k-22481-4^5^ | Vol | 4 | Russia | - | - | - | - | - |
|  | dcn243 | k-22481-5^5^ | Vol | 4 | Russia | - | - | - | - | - |
|  | dcn244 | k-22481-6^5^ | Vol | 4 | Russia | - | - | - | - | - |
|  | dcn245 | k-22482 | Vol | 4 | Russia | Chuvashia | 55° 33' | 46° 55' | - | - |
|  | dcn246 | k-25516 -1,4,5^4^ | hybTrc_Bal | 2 | Russia | Chuvashia, v. Verkhny Kurgan | 55^0^ 40’ | 46^0^ 50’ | - | - |
|  | dcn247 | k-25516 -2^4^ | Vol | 4 | Russia | - | - | - | - | - |
|  | dcn248 | k-25516 -3^4^ | Vol | 4 | Russia | - | - | - | - | - |
|  | dcn249 | k-25516 -6^4^ | Vol | 2 | Russia | - | - | - | - | - |
|  | dcn250 | k-30728-1,2,5^4^ | Trc | 2 | Russia | Nizhny Novgorod, v. Simbeley | 56° 19' | 43° 59' | - | - |
|  | dcn251 | k-30728-3,6^4^ | Trc | 2 | Russia | - | - | - | - | - |
|  | dcn252 | k-30728-4^4^ | Vol | 4 | Russia | - | - | - | - | - |
|  | dcn253 | k-30728-P^4^ | Vol | 4 | Russia | - | - | - | - | - |
|  | dcn254 | k-33153a^2^ | Trc | 2 | Russia | Perm Region | 57° 29' 37" | 55° 43' 53" | - | - |
|  | dcn255 | k-33153b^2^ | Trc | 2 | Russia | - | - | - | - | - |
|  | dcn256 | k-37396 | Trc | 2 | Russia | Kabardino-Balkaria, v. Shaurdat | 43^0^ 07’ | 43^0^ 25’ | - | - |
|  | dcn257 | k-38152 | Trc | 2 | Russia | Kabardino-Balkaria, region v. Cherek (Babugent) | 43^0^ 15’ | 43^0^ 32’ | - | - |
|  | dcn258 | k-40606-1,10^2^ | hybTrc_Vol | 2 | Russia | Bashkortostan, Belebey, state farm | 54^0^ 06’ | 54^0^ 07’ | - | - |
|  | dcn259 | k-40606-9^2^ | Trc | 2 | Russia | - | - | - | - | - |
|  | dcn260 | k-417-12^4^ | Vol | 4 | Russia | ‘Polupolba’, Orenburg Region, v. Pavlovka | 54^0^ 13’ | 52^0^ 52’ | - | - |
|  | dcn261 | k-417-2,6^4^ | Vol | 3 | Russia | - | - | - | - | - |
|  | dcn262 | k-417-7^4^ | Vol | 4 | Russia | - | - | - | - | - |
|  | dcn263 | k-417-10,11^4^ | Vol | 4 | Russia | - | - | - | - | - |
|  | dcn264 | k-42065-2,3,4,5^2^ | Vol | 4 | Russia | ‘Polba-3’, Udmurtia | 57° 04' 01" | 53° 01' 40" | - | - |
|  | dcn265 | k-42065-6^2^ | hybTrc_Vol | 2 | Russia | - | - | - | - | - |
|  | dcn266 | k-6246-1^5^ | Bal | 4 | Russia | Ulyanovsk region, Novouzensk | 50^0^ 29’ | 48^0^ 09’ | - | - |
|  | dcn267 | k-6246-2^5^ | Vol | 4 | Russia | - | - | - | - | - |
|  | dcn268 | k-6246-3^5^ | Vol | 2 | Russia | - | - | - | - | - |
|  | dcn269 | k-6246-4^5^ | Vol | 2 | Russia | - | - | - | - | - |
|  | dcn270 | k-6246-5^5^ | Vol | 4 | Russia | - | - | - | - | - |
|  | dcn271 | k-6249-3,4,5,6,9^2^ | Trc | 2 | Russia | Ulyanovsk region, v. Abramovka | 54^0^ 12’ | 47^0^ 41’ | - | - |
|  | dcn272 | k-6249-8^2^ | Trc | 2 | Russia | - | - | - | - | - |
|  | dcn273 | k-64408 | Bal | 4 | Russia | ‘Belka’, Leningrad region | 59° 57' | 30° 18' | - | - |
|  | dcn274 | k-64738 | Trc | 2 | Russia | ‘Runo’, Krasnodar |  |  |  | - |
|  | dcn275 | k-7490-1^4^ | hybTrc_Bal | 4 | Russia | Perm region, v. Sosnovo | 57^0^ 40’ | 54^0^ 35’ | - | - |
|  | dcn276 | k-7490-2^4^ | Trc | 2 | Russia | - | - | - | - | - |
|  | dcn277 | k-7490-7^4^ | hybTrc_Bal | 4 | Russia | - | - | - | - | - |
|  | dcn278 | k-7490-9^4^ | Trc | 2 | Russia | - | - | - | - | - |
|  | dcn279 | k-7492 | Vol | 4 | Russia | Kirov region, Nolinsk | 57^0^ 33’ | 49^0^ 59’ | - | - |
|  | dcn280 | k-7508a^2^ | Trc | 2 | Russia | Yekaterinburg region, Krasnoufimsk | 56^0^ 36’ | 57^0^ 45’ | - | - |
|  | dcn281 | k-7508b^2^ | Trc | 2 | Russia | - | - | - | - | - |
|  | dcn282 | k-7514 | Vol | 4 | Russia | Yekaterinburg region, Chistopol | 55^0^ 21’ | 50^0^ 37’ | - | - |
|  | dcn283 | k-7516 | Trc | 2 | Russia | Ulyanovsk region, v. Sredne-Timersyany | 54^0^ 32’ | 47^0^ 43’ | - | - |
|  | dcn284 | k-859-1,4^2^ | Vol | 4 | Russia | Orenburg region, Buguruslan | 53^0^ 39’ | 52^0^ 26’ | - | - |
|  | dcn285 | k-859-2,3^2^ | Trc | 2 | Russia | - | - | - | - | - |
|  | dcn286 | k-9934 | WEM-1 | 3 | Russia | Polba krasnaya, Leningrad region, v. Porechye, state farm | 59^0^ 45’ | 31^0^ 16’ | - | - |
|  | dcn287 | PI 41025a^3^ | Vol | 4 | Russia | Samara | 53^0^ | 50^0^ | - | - |
|  | dcn288 | PI 41025b^3^ | Vol | 4 | Russia | - | - | - | - | - |
|  | dcn289 | PI 41025c^3^ | Trc | 2 | Russia | - | - | - | - | - |
|  | dcn290 | PI 94616 | Bal | 4 | Russia | Ural region | 56° 50' | 60° 35' | - | - |
|  | dcn291 | PI 94660-1^2^ | Vol | 4 | Russia | Yaroslavl region | 57° 57' | 38° 24' | 121 | - |
|  | dcn292 | PI 94660-2^2^ | Vol | 2 | Russia | - | - | - | - | - |
|  | dcn293 | PI 94676 | Eth | 1 | Russia | Krasnodar, Otrada, Kubanska | 45° 18' | 40° 54' | 116 | IG 45311 |
|  | dcn294 | PI 94677 | Eth | 1 | Russia | Krasnodar, Otrada, Kubanska | 45° 18' | 40° 54' | 116 | - |
|  | dcn295 | PI 94664 | Eth | 1 | Saudi Arabia | Unknown (from VIR, ‘Early WEM-Spelt') | no data | no data | - | - |
|  | dcn296 | IG 127692 | hybWEM_WEM-Sp | 3 | WEM-Spain | ‘Escandia’, Navarra, v. Aldaz | 43^0^ 00’ | -0° 11' | 584 | - |
|  | dcn297 | IG 45096 | WEM-1 | 3 | WEM-Spain | Navarra, v. Sarasa | 42° 49' | -1° 45' | 584 | - |
|  | dcn299 | INRA 27076 | WEM-1 | 3 | WEM-Spain | unknown | no data | no data | - | - |
|  | dcn300 | INRA 27094 | WEM-1 | 3 | WEM-Spain | unknown | no data | no data | - | - |
|  | dcn301 | k-20410 | WEM-1 | 3 | WEM-Spain | Castilla la Mancha, 61 km S of Cuenca | 39° 57' 30" | -1° 51' 13" | 946 | - |
|  | dcn302 | k-20541 | WEM-1 | 3 | WEM-Spain | Asturias, Palakios, Pola-de-Lena | 43° 09' 05" | -4° 11' 15" | 606 | - |
|  | dcn303 | k-20579a^2^ | WEM-Sp | 3 | WEM-Spain | Asturias, Pinevas, Aller | 43° 10' | -5°38' | - | - |
|  | dcn304 | k-20579b^2^ | WEM-Sp | 3 | WEM-Spain | - | - | - | - | - |
|  | dcn305 | k-21177 | hybWEM_WEM-Sp | 3 | WEM-Spain | Navarra, v. ZaBal | 42^0^ 43’ | 2^0^ 00’ | - | - |
|  | dcn306 | k-21278 | WEM-1 | 3 | WEM-Spain | Navarra, t. Pamplona | 42^0^ 49’ | 1^0^ 39’ | - | - |
|  | dcn307 | PI 190920 | WEM-Sp | 3 | WEM-Spain | Zaragoza | 41° 39' | -0° 54' | - | IG 88765 |
|  | dcn308 | PI 191091 | WEM-Sp | 3 | WEM-Spain | Oviedo, Malvedo | 43° 06' | -4° 12' | 484 | - |
|  | dcn309 | PI 254193 | WEM-1 | 3 | WEM-Spain | Zaragoza, selection from PI 190926 | 41°35' | -1^0^ 00’ | - | - |
|  | dcn310 | PI 256031 | WEM-Sp | 3 | WEM-Spain | Oviedo, Malvedo | 43° 06' | -5° 48' | 484 | - |
|  | dcn311 | PI 275997 | WEM-1 | 3 | WEM-Spain | Huesca, Graus | 43° 23' | -6° 04' | 56 | - |
|  | dcn312 | PI 275998 | WEM-Sp | 3 | WEM-Spain | Oviedo, Puente Fierros | 43° 04' | -5° 46' | 272 | IG 92547 |
|  | dcn313 | PI 275999 | WEM-Sp | 3 | WEM-Spain | Oviedo, Puente Fierros | 43° 04' | -5° 46' | 272 | - |
|  | dcn314 | PI 276002 | hybWEM_WEM-Sp | 3 | WEM-Spain | Cuenca, Canada del Hoyo | 39°58' | -1° 54' | 1001 | - |
|  | dcn315 | PI 276003 | WEM-Sp | 3 | WEM-Spain | Navarra | 42° 45' | -1° 40' | - | - |
|  | dcn316 | PI 276004 | WEM-1 | 3 | WEM-Spain | Huesca, Graus | 42° 11' | 0° 20' | 540 | - |
|  | dcn317 | PI 276005 | WEM-Sp | 3 | WEM-Spain | Oviedo, Somiedo | 43° 06' | -6° 5' | 1012 | - |
|  | dcn318 | PI 276009a^2^ | WEM-Sp | 3 | WEM-Spain | La Coruna, Muros | 42°47' | -9° 02' | 0 | - |
|  | dcn319 | PI 276009b^2^ | WEM-Sp | 3 | WEM-Spain | - | - | - | - | - |
|  | dcn320 | PI 276010 | WEM-Sp | 3 | WEM-Spain | Oviedo, Salas | 43° 24' | -6° 16' | 297 | - |
|  | dcn321 | PI 276013a^2^ | WEM-Sp | 3 | WEM-Spain | Oviedo, Somiedo | 43° 06' | -6° 15' | 1012 | - |
|  | dcn322 | PI 276013b^2^ | WEM-Sp | 3 | WEM-Spain | - | - | - | - | - |
|  | dcn323 | PI 276016 | WEM-Sp | 3 | WEM-Spain | Oviedo, Vega Peridiello | 43°2 1' | -6° 01' | 185 | - |
|  | dcn324 | PI 277671 | WEM-Sp | 3 | WEM-Spain | Navarra | 42° 45' | -1° 40' | - | IG 45398 |
|  | dcn325 | PI 308879 | WEM-2 | 2 | WEM-Spain | Zaragoza | 41° 35' | -1^0^ 00’ | - | - |
|  | dcn326 | PI 352336 | WEM-1 | 3 | WEM-Spain | Oviedo ‘Asturie B6’ | 43° 22’ | -5° 51' | - | - |
|  | dcn327 | k-36527 | WEM-1 | 3 | Sweden | unknown | no data | no data | - | - |
|  | dcn328 | TRI 19294 | WEM-2 | 3 | Sweden | ‘von Kojanus, Landskrona’ | no data | no data | - | - |
|  | dcn329 | INRA 23757 | WEM-2 | 3 | Switzerland | unknown | no data | no data | - | - |
|  | dcn330 | k-12946 | WEM-1 | 3 | Switzerland | unknown | no data | no data | - | - |
|  | dcn331 | k-14292 | hybTrc_WEM | 3 | Switzerland | from Zürich Botanical Garden | 47^0^ 22’ | 08^0^ 32’ | - | - |
|  | dcn332 | PI 277129 | WEM-2 | 3 | Switzerland | Zürich, Experiment Station, Zurich-Oerlikon | 47^0^ 25’ | 8^0^ 33’ | 439 | - |
|  | dcn333 | PI 355467 | WEM-1 | 3 | Switzerland | unknown | no data | no data | - | - |
|  | dcn334 | PI 352348 | WEM-2 | 1 | Syria | Unknown | no data | no data | - | - |
|  | dcn335 | PI 355498 | WEM-2 | 3 | Syria | Unknown | no data | no data | - | - |
|  | dcn336 | IG 45087 | Trc | 2 | Turkey | Kars, 12 km SW of Arpacay | 40° 46' | 43° 17' | 1590 | - |
|  | dcn337 | IG 45089 | Irn | 2 | Turkey | Kars, 12 km SW of Arpacay | 40° 46' | 43° 17' | 1590 | - |
|  | dcn338 | IG 45128 | Trc | 2 | Turkey | Kars, 12 km SW of Arpacay | 40° 46' | 43° 17' | 1590 | - |
|  | dcn339 | IG 45335 | Trc | 2 | Turkey | Kars, 12 km SW of Arpacay | 40° 46' | 43° 17' | 1590 | - |
|  | dcn340 | IG 45336 | Trc | 2 | Turkey | Kars, 12 km SW of Arpacay | 40° 46' | 43° 17' | 1590 | - |
|  | dcn341 | INRA 27113 | Eth | 1 | Turkey | unknown | no data | no data | - | - |
|  | dcn342 | k-14380 | Bal | 4 | Turkey | Kastamonu, Tosya | 41° 23' | 33° 49' | 863 | - |
|  | dcn343 | k-20967 | Bal | 4 | Turkey | Kastamonu, Khatran-Dagh mountain range, Paphlagonia | 41^0^ 23’ | 33^0^ 46’ | 1200 | - |
|  | dcn344 | k-20968 | Bal | 4 | Turkey | Kastamonu, Khatran-Dagh mountain range, Paphlagonia | 41^0^ 23’ | 33^0^ 46’ | 1200 | - |
|  | dcn345 | k-20969 | WEM-1 | 3 | Turkey | Kastamonu, Taşköprü | 41° 31' | 34° 13' | 571 | - |
|  | dcn346 | k-20993 | Bal | 4 | Turkey | Kastamonu, Khatran-Dagh mountain range, Paphlagonia | 41^0^ 23’ | 33^0^ 46’ | 1200 | - |
|  | dcn347 | k-21007 | Bal | 4 | Turkey | around Kastamonu city | 41^0^ 23’ | 33^0^ 46’ | 1200 | - |
|  | dcn348 | PI 182743 | WEM-2 | 2 | Turkey | Mardin, Derik | 37° 22' | 40° 16' | 725 | - |
|  | dcn349 | PI 319868 | Eth | 1 | Turkey | Unknown (collected by J. Harlan) | no data | no data | - | - |
|  | dcn350 | PI 319869 | Eth | 1 | Turkey | Unknown (collected by J. Harlan) | no data | no data | - | - |
|  | dcn351 | PI 352329 | Bal | 3 | Turkey | Kastamonu, Tosya | 41° 01' | 34° 02' | 830 | IG 45325 |
|  | dcn352 | PI 355454 | WEM-1 | 3 | Turkey | Asia Minor | no data | no data | - | - |
|  | dcn353 | PI 355507 | Bal | 4 | Turkey | Kastamonu, Tosya | 41° 01' | 34° 02' | 830 | - |
|  | dcn354 | PI 470737 | Trc | 2 | Turkey | Kars, 12km SW of Arpacay | 40° 46' | 43° 17' | 1590 | TA 2895 |
|  | dcn355 | PI 470738a^2^ | hybTrc_Bal | 2 | Turkey | Kars, 12km SW of Arpacay | 40° 46' | 43° 17' | 1590 | IG 45088 |
|  | dcn356 | PI 470738b^2^ | Trc | 2 | Turkey | - | - | - | - | - |
|  | dcn357 | PI 470739 | Irn | 2 | Turkey | Kars, 12km SW of Arpacay | 40° 46' | 43° 17' | 1590 | IG 45089 |
|  | dcn358 | PI 606325 | Bal | 4 | Turkey | v. Duragan near Sinop. Grown by Muzeffer Basboga | 41° 25' | 35° 03' | 1000 | - |
|  | dcn359 | TRI 17023 | Bal | 4 | Turkey | Kastamonu Province, Sipahiler | 39° 52' | 32° 50' | - | - |
|  | dcn360 | TRI 17058 | hybBal_WEM | 2 | Turkey | Unknown | no data | no data | - | - |
|  | dcn361 | TRI 584 | WEM-1 | 3 | Turkey | ‘Weißer Amidonia’ | no data | no data | - | - |
|  | dcn362 | IG 45408 | Trc | 2 | Ukraine | Kharkiv | 50° 0' | 36° 14' | 176 | - |
|  | dcn363 | k-14999 | Trc | 2 | Ukraine | Lviv | 49° 51' | 24° 01' | - | - |
|  | dcn364 | k-15007a^2^ | WEM-1 | 3 | Ukraine | Lviv | 49° 51' | 24° 01' | - | - |
|  | dcn365 | k-15007b^2^ | WEM-1 | 3 | Ukraine | - | - | - | - | - |
|  | dcn366 | k-19352a^2^ | Bal | 4 | Ukraine | Lviv | 49° 51' | 24° 01' | - | - |
|  | dcn367 | k-19352b^2^ | WEM-1 | 3 | Ukraine | - | - | - | - | - |
|  | dcn368 | k-19360 | WEM-1 | 3 | Ukraine | Lviv | 49° 51' | 24° 01' | - | - |
|  | dcn369 | k-19361 | hybTrc_WEM | 1 | Ukraine | Lviv | 49° 51' | 24° 01' | - | - |
|  | dcn370 | k-19377 | WEM-2 | 1 | Ukraine | Lviv | 49° 51' | 24° 01' | - | - |
|  | dcn371 | PI 94614 | Trc | 2 | Ukraine | Kharkiv | 50° 0' | 36° 14' | - | - |
|  | dcn372 | PI 94738 | Trc | 2 | Ukraine | Kharkiv | 50° 0' | 36° 14' | - | - |
|  | dcn373 | PI 94741 | Vol | 4 | Ukraine | Kharkiv | 50° 0' | 36° 14' | - | - |
|  | dcn374 | IG 99244 | hybTrc_Eth | 1 | Yemen | Dhamar | 14° 18' | 44° 28' | 2200 | - |
|  | dcn375 | k-25459 | hybTrc_Eth | 2 | Yemen | unknown | no data | o data | - | - |
|  | dcn376 | TA 10514 | hybTrc_Eth | 2 | Yemen | Al Dubarin | 13° 52' | 44° 12' | 2088 | - |
|  | dcn377 | TA 10515 | hybTrc_Eth | 1 | Yemen | Maris | 14° 18' | 44° 28' | 2626 | - |
|  | dcn378 | TA 10516 | hybTrc_Eth | 1 | Yemen | 12 km N of Dhamar | 14° 39' | 44° 21' | 2414 | - |
|  | dcn379 | TRI 25486 | hybTrc_Eth | 1 | Yemen | Al Khadira | 15° 21' | 44° 12' | - | - |
|  | dcn380 | TRI 28027a^2^ | Eth | 1 | Yemen | Habur, Wadi below town NN | 15° 21' | 44° 12' | - | - |
|  | dcn381 | TRI 28027b^2^ | Eth | 1 | Yemen | - | - | - | - | - |
|  | dcn382 | TRI 28072a^2^ | hybTrc_Eth | 1 | Yemen | Dawran, 10 km SE of Ma’bar | 15° 21' | 44° 12' | - | - |
|  | dcn383 | TRI 28072b^2^ | hybTrc_Eth | 1 | Yemen | - | - | - | - | - |
|  | dcn384 | TRI 28861 | Eth | 1 | Yemen | 47 km on Saddah-Khawlan track | 15° 20' 54" | 44°12' 23" | - | - |
|  | dcn385 | INRA 27119 | Bal | 4 | Yugoslavia | unknown | no data | no data | - | - |
|  | dcn386 | INRA 27126 | Bal | 4 | Yugoslavia | unknown | no data | no data | - | - |
|  | dcn387 | k-40030 | Bal | 4 | Yugoslavia | unknown, ‘Pirevina’ | no data | no data | - | - |
|  | dcn388 | k-40032a^2^ | Bal | 4 | Yugoslavia | unknown, ‘Pirevina’ | no data | no data | - | - |
|  | dcn389 | k-40032b^2^ | Bal | 4 | Yugoslavia | - | - | - | - | - |
|  | dcn390 | PI 434993 | Bal | 4 | Montenegro | Vranic, Niksic region | 42° 48' | 18° 56' | 628 | - |
|  | dcn391 | IG 45377 | Bal | 4 | Bosnia and Herzegovina | Zevajt, Celebici region | 43° 26' | 18° 53' | 969 | PI 434998 |
|  | dcn392 | k-23036 | Bal | 4 | Bosnia and Herzegovina | Zenica | 44° 18' | 17° 33' | 1282 | - |
|  | dcn393 | k-38904 | Bal | 4 | Bosnia and Herzegovina | Saraevo | 43° 51' | 18° 25' | 550 | - |
|  | dcn394 | k-38915 | Bal | 4 | Bosnia and Herzegovina | Humac vil, Ljubushki region | 43^0^ 12’ | 18^0^ 24’ | 80 | - |
|  | dcn395 | k-38917 | Bal | 4 | Bosnia and Herzegovina | Drinovci | 43^0^ 21’ | 17^0^ 19’ | 287 | - |
|  | dcn396 | PI 362699 | Bal | 4 | Bosnia and Herzegovina | Gacko-Kula Fazlagica | 43° 09' | 18° 32' | 940 | - |
|  | dcn397 | PI 374685 | Bal | 4 | Bosnia and Herzegovina | Donji Budanj-Foca | 43° 31' | 18° 40' | 678 | - |
|  | dcn398 | PI 434995a^2^ | Bal | 4 | Bosnia and Herzegovina | Lug, Trebinje area | 42° 47' | 18° 13' | 260 | - |
|  | dcn399 | PI 434995b^2^ | Bal | 4 | Bosnia and Herzegovina | - | - | - | - | - |
|  | dcn400 | PI 434998 | Bal | 4 | Bosnia and Herzegovina | Zavajt, Celebici region | 43° 25' | 18° 53' | 963 | - |
|  | dcn401 | PI 434999a^3^ | Bal | 4 | Bosnia and Herzegovina | Kule Fazlagica, Gacko Region | 43° 08' | 18° 31' | 950 | - |
|  | dcn402 | PI 434999b^3^ | Bal | 4 | Bosnia and Herzegovina | - | - | - | - | - |
|  | dcn403 | PI 434999c^3^ | Bal | 4 | Bosnia and Herzegovina | - | - | - | - | - |
|  | dcn404 | PI 264964 | Bal | 4 | Croatia | Licko-senjska zupan, GoWEM-Spic-Senj road | 44° 53' | 15° 13' | 566 | - |
|  | dcn405 | IG 45406a^2^ | Bal | 4 | Montenegro | Zupa-Ducic, Niksic region | 42° 46' | 18° 49' | 967 | - |
|  | dcn406 | IG 45406b^2^ | Bal | 4 | Montenegro | - | - | - | - | - |
|  | dcn407 | PI 362696 | Bal | 4 | Montenegro | Dusca-Niksic | 42° 21' | 19° 18' | 34 | - |
|  | dcn408 | PI 362697 | Bal | 4 | Montenegro | Potkovaca-Pjevlje | 43° 28' | 19° 06' | 975 | - |
|  | dcn409 | PI 434992 | Bal | 4 | Montenegro | Vranic, Niksic region | 42° 48' | 18° 56' | 628 | - |
|  | dcn410 | PI 434996 | Bal | 4 | Montenegro | Otilovic, Pljevlja region | 43° 19' | 19° 26' | 1098 | - |
|  | dcn411 | PI 434997a^2^ | Bal | 4 | Montenegro | Otilovic, Pljevlja region | 43° 19' | 19°26' | 1098 | - |
|  | dcn412 | PI 434997b^2^ | Bal | 4 | Montenegro | - | - | - | - | - |
|  | dcn413 | PI 254192 | Bal | 4 | Serbia | Belgrade | 44 50' | 20° 30' | 71 | - |
|  | dcn414 | PI 265004 | Bal | 4 | Serbia | near Zrze, 20 km from Prizren | 42° 05' | 20° 41' | 1187 | - |
|  | dcn415 | PI 345126 | WEM-2 | 3 | Serbia | Belobaba-Prijepolje | 43° 21' | 19° 42' | 637 | - |
|  | dcn416 | PI 345471 | Bal |  | Serbia | Kokin Brod-Donja Bistrica | 43° 31' | 19° 48' | 956 | - |
|  | dcn417 | PI 350000 | Bal |  | Serbia | Bistrica | 43° 28' | 19° 42' | 643 | - |
|  | dcn418 | PI 362438 | Bal |  | Serbia | Bistrica-Tara | 43° 28' | 19° 42' | 643 | - |
|  | dcn419 | PI 362439 | Bal |  | Serbia | Bistrica-Tara | 43° 28' | 19° 42' | 643 | - |
|  | dcn420 | PI 362500 | Bal |  | Serbia | Knjazevac | 43° 28' | 19° 42' | 643 | - |
|  | dcn421 | PI 362501a^2^ | Bal |  | Serbia | Knjazevac | 43° 35' | 22° 15' | 225 | - |
|  | dcn422 | PI 362501b^2^ | Bal |  | Serbia | - | - | - | - | - |
|  | dcn423 | PI 434994 | Bal |  | Serbia | Zupa-Ducic, Niksic region | 42° 46' | 18° 49' | - | - |
|  | dcn424 | PI 94657 | Bal |  | Serbia | Belgrade | 44° 50' | 20° 30' | 71 | - |
|  | dsc001 | IG 113301a^2^ | Dsc |  | Iran | Ilam | 33° 37' | 46° 27' | 1456 | - |
|  | dsc002 | IG 113301b^2^ | Dsc | - | Iran | - | - | - | - | - |
|  | dsc003 | IG 113302 | Dsc | - | Iran | Ilam | 33° 37' | 46° 27' | -1456 | - |
|  | dsc004 | KU-8942 | Dsc | - | Iran | 58.8 km N from Kermanshah to Ravansar | 34° 43' | 46° 39' |  | - |
|  | dsc005 | PI 428016 | Dsc | - | Iran | Bakhtaran, 50 km W of Shahabad | 34° 22' | 46° 06' | 1721 | - |
|  | dsc006 | IG 109085 | Dsc |  | Iraq | Ninawa, Top of Jebel Sinjar | 36° 23' | 41° 47' | 1220 | - |
|  | dsc007 | IG 131232 | Dsc |  | Iraq | SSW of Rowanduz | 36° 36' | 44° 29' | 580 | KU-8737 |
|  | dsc008 | IG 131233 | Dsc |  | Iraq | 53 km ENE from Dohuk to Amadiyah Rowanduz | 37° 06' | 43° 38' | 780 | KU-8821A |
|  | dsc009 | k-42632 | Dsc |  | Iraq | unknown | no data | no data | - | - |
|  | dsc010 | KU-8537 | Dsc |  | Iraq | 20.3 km S from Sulaymaniyah to Qara Dagh | 35^0^ 29’ | 45^0^ 19’ 31’’ | - | - |
|  | dsc011 | UH-202A | Dsc |  | Israel | Giv’at-Ko’akh | 32° 02' | 34° 57' | 115 | - |
|  | dsc012 | UH-321H | Dsc |  | Israel | West Bank, Mt. Gerizim, S of Nablus | 32° 12' | 35° 16' | 881 | - |
|  | dsc013 | UH-8915H | Dsc |  | Israel | Jordan valley, Gitit | 32° 06' | 35° 24' | 306 | - |
|  | dsc014 | UH-359 | Dsc |  | Israel | Amirim | 32° 56' | 35° 27' | 537 | - |
|  | dsc015 | UH-A17 | Dsc |  | Israel | Arbel | 32° 49' | 35° 50' | 136 | - |
|  | dsc016 | UH-A3 | Dsc |  | Israel | Arbel | 32° 49' | 35° 50' | 136 | - |
|  | dsc017 | UH-A4 | Dsc |  | Israel | Arbel | 32° 49' | 35° 50' | 136 | - |
|  | dsc018 | UH-G1-6 | Jud |  | Israel | Gamla | 32° 54' | 35° 45' | 369 | - |
|  | dsc019 | UH-Gw1 | Jud |  | Israel | Gamla | 32° 54' | 35° 45' | 369 | - |
|  | dsc020 | IG 46301 | Dsc |  | Israel | Hazafon, Rosh Pinna | 32° 58' | 35° 32' | 600 | - |
|  | dsc021 | IG 46303 | Dsc |  | Israel | West Bank, Sanhedriyya | 31° 48' | 35° 13' | 750 | - |
|  | dsc022 | IG 46306 | Dsc |  | Israel | Hadarom, Kokhav Hashahar | 31° 38' | 34° 40' | 140 | - |
|  | dsc023 | UH-J4-1 | Jud |  | Israel | Jehudiyya | 32° 56' | 35° 41' | 151 | - |
|  | dsc024 | UH-J4-2 | Jud |  | Israel | Jehudiyya | 32° 56' | 35° 41' | 151 | - |
|  | dsc025 | UH-J5-2 | Jud |  | Israel | Jehudiyya | 32° 56' | 35° 41' | 151 | - |
|  | dsc026 | KU-14507 | Dsc |  | Israel | Tayiba | 32° 16' | 35° 01' | 91 | - |
|  | dsc027 | UH-MO | Dsc |  | Israel | Mitzpe Ofer | 32° 37' | 34° 59' | - | - |
|  | dsc028 | UH-NO | Dsc |  | Israel | Nahal Orvim | 33° 09' | 35° 41' | 680 | - |
|  | dsc029 | UH-2160 | Dsc |  | Israel | Mt Gilboa | 32° 33' | 35° 21' | 348 | - |
|  | dsc030 | UH-425 | Dsc |  | Israel | Jordan valley, Gitit | 32° 06' | 35° 24' | 306 | - |
|  | dsc031 | UH-NM5 | Dsc |  | Israel | Nahal Mearot | 32° 40' 16" | 34° 58' 21" | 30 | - |
|  | dsc032 | UH-NM6 | Dsc |  | Israel | Nahal Mearot | 32° 40' 16" | 34° 58' 21" | 30 | - |
|  | dsc033 | PI 414719 | Dsc |  | Israel | N Israel, Bet Qeshet, Lower Galilee | 32° 43' 08" | 35° 23' 55" | 229 | - |
|  | dsc034 | PI 414720 | Dsc |  | Israel | Bat Shelomo, Samarian Mountains | 32° 35' 49" | 35° 0' 07" | 105 | - |
|  | dsc035 | PI 414721 | Jud 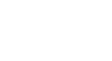 |  | Israel | Mountain of Beatitudes, Korazim Sill | 32° 54' | 35° 32' | 79 | - |
|  | dsc036 | PI 414722 | Dsc |  | Israel | Zefat, Upper Galilee | 31° 46' 59" | 35° 13' |  | - |
|  | dsc037 | PI 428105 | Dsc |  | Israel | N Israel, 1-2 km S of Rosh Pinna towards Safad | 32° 58' | 35° 20' | 549 | - |
|  | dsc038 | PI 466978 | Dsc |  | Israel | Jerusalem, Bet Me’ir | 31° 48' | 35° 20' | 467 | - |
|  | dsc039 | PI 467005 | Jud | - | Israel | N Israel, Tabigha | 32° 52' | 35° 20' | 203 | - |
|  | dsc040 | PI 467014 | Jud | - | Israel | N Israel, Tabigha | 32° 52' | 35° 20' | 203 | - |
|  | dsc041 | PI 467019 | Jud | - | Israel | N Israel, Tabigha | 32° 52' | 35° 20' | 203 | - |
|  | dsc042 | PI 470972 | Dsc | - | Israel | Jerusalem, Bet Me’ir | 31^0^ 48‘ | 35^0^ 02‘ | 467 | - |
|  | dsc043 | PI 471714 | Dsc | - | Israel | Unknown | no data | no data | - | - |
|  | dsc044 | PI 479782 | Dsc | - | Israel | West Bank, Kokhav HaShahar. | 31° 57' | 35° 20' | 696 | - |
|  | dsc045 | PI 538699 | Dsc | - | Israel | West Bank, between Ramla and Jericho, ca. 6 km E of Taibe | 31° 50' | 35° 27' | 249 | - |
|  | dsc046 | TA 1057 | Dsc | - | Israel | Hazafon, 1 km E of Rosh Pinna | 32° 59' | 35° 32' | 549 | - |
|  | dsc047 | TRI 17214 | Jud | - | Israel | unknown | no data | no data |  | - |
|  | dsc048 | UH-TZ124A | Dsc | - | Israel | Gamla | 32° 54' | 35° 45' | 369 | - |
|  | dsc049 | UH-TZ45A | Dsc | - | Israel | Mt Gilboa | 32° 33' | 35° 21' | 348 | - |
|  | dsc050 | UH-TZ51A | Dsc | - | Israel | West Bank, Mt. Gerizim, S of Nablus | 32° 12' | 35° 16' | 881 | - |
|  | dsc051 | UH-H-8-1 | Dsc | - | Israel | Hermon | 33° 18' | 35° 46' | 1481 | - |
|  | dsc052 | IG 115808a^2^ | Dsc | - | Jordan | At Tafilah, Qadisieh 1 km S of the old site A 300m W of TV transmitter | 30° 40' | 35° 37' | 1480 | - |
|  | dsc053 | IG 115808b^2^ | Dsc | - | Jordan | - | - | - | - | - |
|  | dsc054 | IG 139129 | Dsc | - | Jordan | Irbid, Al Hadib | 32° 04' | 35° 44' | 1008 | - |
|  | dsc055 | IG 139130 | Dsc | - | Jordan | Irbid, Al Hadib | 32° 04' | 35° 44' | 1008 | - |
|  | dsc056 | IG 44828 | Dsc | - | Jordan | At Tafilah, Rashadiah near cement factory road Shoubak | 30° 42' | 35° 39' | 1550 | - |
|  | dsc057 | IG 46353 | Dsc | - | Jordan | Amman, Um Al-Ammad 2 km N | 31° 46' | 35° 53' | 760 | - |
|  | dsc058 | IG 46386 | Dsc | - | Jordan | Amman, 7 km W Naur on Dead Sea highway | 31° 51' | 35° 48' | 700 | - |
|  | dsc059 | IG 46486a^2^ | Dsc | - | Jordan | Irbid, Sakhra v. Irbid to Ajlun | 32° 21' | 35° 40' | 1180 | - |
|  | dsc060 | IG 46486b^2^ | Dsc | - | Jordan | - | - | - | - | - |
|  | dsc061 | IG 110815 | Dsc | - | Lebanon | Biqaa, between Kfarkouk and Aiha | 33° 19' | 35° 31' | 1216 | - |
|  | dsc062 | IG 46524 | Dsc | - | Lebanon | Biqaa Al Gharbi, 2 km from Sohmbr E of Kfar Mechki | 33° 31' | 35° 43' | 980 | - |
|  | dsc063 | IG 46526 | Dsc | - | Lebanon | Rachaiya before Ain Hircha road from Rashaiya | 33° 27' | 35° 49' | 1020 | - |
|  | dsc064 | IG 46528 | Dsc | - | Lebanon | Baalbek, 500m before the end of v. Sa'ide road to Zahle | 34° 01' | 36° 05' | 1080 | - |
|  | dsc065 | IG 46531a^2^ | Dsc | - | Lebanon | Rachaiya, 6 km from Kfar Qoug towards Bakaa | 33° 34' | 35° 54' | 1470 | - |
|  | dsc066 | IG 46531b^2^ | Dsc | - | Lebanon | - | - | - | - | - |
|  | dsc067 | PI 352322 | Dsc | - | Lebanon | Nabatiye, Mt. Hermon | 33° 25' | 35° 52' | 2655 | - |
|  | dsc068 | PI 355455 | WEM-2 | - | Lebanon | Nabatiye, Mt. Hermon Minor | 33° 25' | 35° 52' | 2655 | - |
|  | dsc069 | PI 470978 | Dsc | - | Lebanon | Nabatiye, Mt. Hermon | 33° 25' | 35° 52' | 2655 | - |
|  | dsc070 | TRI 18478 | Dsc | - | Lebanon | El Beqaa, Aiha-Kfarkouk, above ‘sahlet’ | 33° 31' | 35° 52' | 1508 | - |
|  | dsc071 | IG 117894 | Dsc | - | Syria | Aleppo, 4 km N of St. Simeon road to Afrin | 36° 22' | 36° 51' | 400 | - |
|  | dsc072 | IG 119408 | Dsc | - | Syria | Homs, 2 km W of Garaata to Kafr Na'an 6km W Homs-Hama highway | 34° 53' | 36° 40' | 395 | - |
|  | dsc073 | IG 119430 | Dsc | - | Syria | Homs, Deir Ful 4 km NE of the village. Protected forest plantation (similar area 2 km N) | 34° 57' | 36° 51' | 470 | - |
|  | dsc074 | IG 119437 | Dsc | - | Syria | Hama, 4 km SW of Bsirin | 35^0^ 00’ | 36° 42’ | 340 | - |
|  | dsc075 | IG 119450 | Dsc | - | Syria | Idlib, Kafr Nabil; 2 km S | 35° 36’ | 36° 33' | 560 | - |
|  | dsc076 | IG 139957 | Dsc | - | Syria | Dar'a, Saham | 32° 47' | 35° 59' | 463 | - |
|  | dsc077 | IG 139970 | Dsc | - | Syria | Sweida, S of Mushanaf | 32° 43' | 36° 47' | 1582 | - |
|  | dsc078 | IG 139993 | Dsc | - | Syria | Damascus, Ras Al Nabe'a 5 km W of N. Jeroudeih Beit Jin | 33° 20' | 35° 51' | 1288 | - |
|  | dsc079 | IG 45298 | Dsc | - | Syria | Aleppo, Abbeen; Deir Al Jamal | 32° 47' | 35° 59' | 463 | - |
|  | dsc080 | IG 45506 | Dsc | - | Syria | Aleppo, Aleppo-Abeen road after Aleppo-Afrin road | 36^0^ 30’ | 37^0^ 00’ | 350 | - |
|  | dsc081 | IG 46439 | Dsc | - | Syria | Sweida, 4 km E of Junction from Sewida and Salkhad | 32° 29' | 36° 37' | 1447 | - |
|  | dsc082 | IG 46472 | Dsc | - | Syria | Damascus, end of Wadi El Hanoun 6 km W Rawda | 33^0^ 38’ | 35^0^ 57’ | 1280 | - |
|  | dsc083 | k-17157 | WEM-2 | - | Syria | Dar'a region, v. An Namir | 32^0^ 47’ | 36^0^ 12’ |  | - |
|  | dsc084 | PI 470944 | Dsc | - | Syria | Al Qunaytirah, Kazrin | 32^0^ 59' | 35^0^ 41‘ | 388 | - |
|  | dsc085 | IG 116171 | Dsc | - | Turkey | Gaziantep, Magaracik village on the way from Polateli; 4 km from Polateli | 37°9' | 36° 51' | 640 | - |
|  | dsc086 | IG 116174 | Dsc | - | Turkey | Gaziantep, 2 km E of Gebe | 37° 29' | 36° 47' | 605 | - |
|  | dsc087 | IG 116178 | Dsc | - | Turkey | Gaziantep, 3 km from Ugruca v. toward Gazintep | 37° 29' | 37° 15' | 775 | - |
|  | dsc088 | IG 116179a^3^ | Dsc | - | Turkey | Gaziantep, 1 km N of Akcaburc | 37° 20' | 37° 15' | 830 | - |
|  | dsc089 | IG 116179b^3^ | Dsc | - | Turkey | - | - | - | - | - |
|  | dsc090 | IG 116179c^3^ | Dsc | - | Turkey | - | - | - | - | - |
|  | dsc091 | IG 116181 | Dsc | - | Turkey | Gaziantep, 2 km from Kara Yusuflu road to Yavuzeli | 37° 28' | 37° 19' | 750 | - |
|  | dsc092 | IG 46149 | Dsc | - | Turkey | Urfa, 27 km E Siverek | 37° 40' | 39° 33' | 1100 | - |
|  | dsc093 | IG 46171 | Dsc | - | Turkey | Urfa, 27 km E Siverek | 37° 40' | 39° 33' | 1100 | - |
|  | dsc094 | IG 46183 | Dsc | - | Turkey | Urfa, 27 km E Siverek | 37° 40' | 39° 33' | 1100 | - |
|  | dsc095 | IG 46185 | Dsc | - | Turkey | Urfa, 27 km E Siverek | 37° 40' | 39° 33' | 1100 | - |
|  | dsc096 | IG 46250 | Dsc | - | Turkey | Diyarbakir, 36.2 km W Diyarbakir in the Karakadaj | 37° 56' | 40° 31' | 1200 | - |
|  | dsc097 | PI 596289 | Dsc | - | Turkey | Diyarbakir, 20 km E of Karacadag on road Siverek to Diyarbakir, 7 km E of v. Karabache | 37° 51' | 39° 49' | 1320 | - |
|  | dsc098 | KU-1952 | Dsc | - | Turkey | Maras, 45 km SE of Maras (Maras - Gaziantep) | 37° 35' | 36° 56' | - | - |
|  | dsc099 | KU-1957 | Dsc | - | Turkey | Maras, 45 km SE of Maras (Maras - Gaziantep) | 37° 35' | 36° 56' | - | - |
|  | dsc100 | KU-1972B | Dsc | - | Turkey | Maras, 45 km SE of Maras (Maras - Gaziantep) | 37° 35' | 36° 56' | - | - |
|  | dsc101 | KU-1991 | Dsc | - | Turkey | Maras, 45 km SE of Maras (Maras - Gaziantep) | 37° 35' | 36° 56' | - | - |
|  | dsc102 | PI 428045 | Dsc | - | Turkey | 36.2 km W of Diyarbakir in the Karacadag | 37° 53' | 39° 52' | 1200 | - |
|  | dsc103 | PI 428051 | Dsc | - | Turkey | 36.2 km W of Diyarbakir in the Karacadag | 37° 53' | 39° 52' | 1200 | - |
|  | dsc104 | PI 428063 | Dsc | - | Turkey | 51 km W of Diyarbakir in the Karacadag | 37° 48' | 39° 46' | 1400 | - |
|  | dsc105 | PI 428145 | Dsc | - | Turkey | Mardin, 34.9 km W of Idil | 37° 17' | 41° 36' | 1000 | - |
|  | dur001 | d | Dur | - | Egypt | landrace, unknown site, provided by E. Hussein | no data | no data | - | - |
|  | dur002 | e | Dur | - | Egypt | - | - | - | - | - |
|  | dur003 | k-38439 | Dur | - | Kyrgyzstan | unknown, ‘Kubanka Karakolskaya’ | 42° 52' 30" | 74° 36' 44" | - | - |
|  | dur004 | k-38390 | Dur | - | Russia | ‘Melyanopus-1932’, Saratov | 51° 32' | 46° 01' | - | - |
|  | dur005 | k-38399 | Dur | - | Russia | ‘Caesium 3/10’, unknown | no data | no data | - | - |
|  | dur006 | k-39099 | Dur | - | Russia | ‘Chakinskaya 226’, Tambov region | 52° 43' | 41° 34' | - | - |
|  | dur007 | k-42422 | Dur | - | Russia | Krasnokutka-10, south Ural | 51° 33' | 46° 02' |  | - |
|  | dur008 | k-59890 | Dur | - | Russia | ‘Bezenchukskaya-182’, from N.M. Tulaikov Samarsky Agricultural-Research Institute, Samara | 51° 33' | 46° 02' | 158 | - |
|  | dur009 | k-62650 | Dur | - | Russia | ‘Valentina’, from ARISER, Saratov | 51° 33' | 46° 02' | 158 | - |
|  | dur010 | k-54976 | Dur | - | Ukraine | Kharkiv | 50° 0' | 36° 14' | - | - |
|  | isp001 | TRI 6177 | Irn | - | Iran | Sengbaran, 31 km on round trip | 35° 42' | 51° 25' | - | - |
|  | isp02 | TRI 7117 | Irn | - | Iran | Sengbaran, 31 km on round trip | 35° 42' | 51° 25' | - | - |
|  | isp003 | TRI 7257 | Irn | - | Iran | unknown | no data | no data | - | - |
|  | isp004 | TRI 7260 | Irn | - | Iran | unknown, from Arboretum and Botanical Garden Ottawa | no data | no data | - | - |
|  | kar001 | TRI 4568 | WEM-2 | - | Georgia | unknown, from NIAVT Tápiószele, Hungary | no data | no data | - | - |
|  | kar002 | TRI 4607 | WEM-2 | - | Georgia | unknown, from sort. E. Schiemann: T 606/54, 174 | no data | no data | - | - |
|  | kar003 | TRI 7486 | WEM-2 | - | Georgia | Unknown (obtained from VIR, k-28205) | no data | no data | - | - |
|  | kar004 | IM-1 | WEM-2 | - | Georgia | unknown, from Tbilisi Botanical Institute (provided by Dr. I. Matitashvili) | no data | no data | - | - |
|  | kar005 | TRI 9630 | WEM-2 | - | Georgia | unknown | no data | no data | - | - |
|  | Material studied by C-banding but not considered in NJ and Gap-nmds analyses |  |  |  |  |  |  |  |  |  |
|  | * | INRA 27125 | Bal | - | Germany | unknown | - | - | - | - |
|  | * | PI 41024 | Eth | - | Russia | Samara (provided in 1915 by Robert Regel, Bureau of Applied Botany, St. Petersburg, Russian Federation, k-417) | - | - | - | - |
|  | * | PI 275996 | Eth | - | WEM-Spain | Huesca, Graus | - | - | - | - |
|  | * | k-19362 | WEM-1 | - | Ukraine | L'viv | - | - | - | - |
|  | * | PI 57536 | Eth | - | Ukraine | Dnepropetrovsk (obtained from Bureau of Applied Botany, Leningrad, Russian Federation, k-1466) | - | - | - | - |
|  | * | k-51768 | WEM-1 | - | Uzbekistan | Uzbekistan | - | - | - | - |
|  | * | IG 45423 | WEM-1 | - | Belgium | unknown (obtained from USDA, PI 355476) | - | - | - | - |
|  | * | INRA 26647 | WEM-1 | - | France | unknown | - | - | - | - |
|  | * | INRA 26648 | WEM-1 | - | France | unknown | - | - | - | - |
|  | * | INRA 26657 | WEM-1 | - | France | unknown | - | - | - | - |
|  | * | INRA 26659 | WEM-1 | - | France | unknown | - | - | - | - |
|  | * | TRI 4342 | WEM-1 | - | Kuwait | unknown | - | - | - | - |
|  | * | k-35890 | WEM-1 | - | NEtherlands | unknown | - | - | - | - |
|  | * | k-20638 | WEM-1 | - | WEM-Spain | Madrid, seed farming station | - | - | - | - |
|  | * | PI 79899 | Trc | - | China | Heilongjiang, 1.Harbin Experiment Station | - | - | - | - |
|  | * | TA 10480 | uncertain | - | Turkey | 9 km NE of Pazarcik, Kahramanmaraş Province. Obtained from ICARDA, IG 44976) | - | - | - | - |
|  | * | k-47795 | Trc | - | Russia | Leningrad region | - | - | - | - |
|  | * | IG 45337 | Vol | - | Portugal | Lisboa (obtained from USDA, PI 56234) | - | - | - | - |
|  | * | PI 278644 | WEM-2 | - | Great Britain | Unknown (obtained from Dept. of Scientific & Industrial Res. Crops Research Division Christchurch, South Island, New Zealand | - | - | - | - |
|  | * | PI 352332 | WEM-2 | - | Belgium | Namur, Station d'Amelioration des Plantes (provided by M. Ingold, Federal Agricultural Research Station, Nyon, Vaud, Switzerland, T-430) | - | - | - | - |
|  | * | k-44167 | Trc | - | India | Delhi | - | - | - | - |
|  | * | k-46482 | Bal | - | India | unknown, ‘NP 201’ | - | - | - | - |
|  | * | INRA 23799 | uncertain | - | China | unknown | - | - | - | - |
|  | * | KU-112 | Eth | - | China | ’Khapli’, Peiping breeding station | - | - | - | - |
|  | * | INRA 27085 | hybTrc_Eth | - | Saudi Arabia | unknown | - | - | - | - |

Accession code: dcn – *T. dicoccon;* dcs – *T.* *dicoccoides;* dur – *T. durum;* isp – *T. ispahanicum;* kar – *T. karamyschevii.* *lines excluded from the analysis.

Accession numbers: k-numbers – VIR, Russia; TRI-numbers – IPK, Germany; IG-numbers – ICARDA, Syria; PI-numbers – USDA-ARS, USA; INRA-numbers **–** INRA, France; TA-numbers **–** WGRC, USA; KU-numbers – Kyoto University, Japan; UH-numbers – University of Haifa, Israel. Two Egyptian landraces of *T. durum* (d, e) were provided by Dr. E.A. Hussein, Al-Azhar University, Cairo, Egypt; the seeds of IM-1 accession of *T. karamyschevii* were provided by Dr. I.M. Matitashvili, Institute of Botany, Tbilisi, Georgia. Superscripts ^2-5^ – the number of lines discriminated within the accession.

Karyotypic group determined on the basis of visual analysis: Wem-1, Wem-2 and WEM-Sp – chromosomal types within the European group; Irn and Trc – chromosomal types within the Asian group; Bal and Vol chromosomal types within the Balkan group; Eth – Ethiopian group; Dur and Mor – Durum and Moroccan chromosomal types; Dcs – type of banding typical for *T. dicoccoides*; Jud – type of banding typical for *T. dicoccoides* subsp. *judaicum*; hybTrc_Dur – hybrid between Trc type emmer and *T. durum*; hybWEM_WEM-sp – hybrid between Wem-1 and Spanish types; hybWEM_Bal – hybrid between WEM and Bal types; hybWEM_Dur – hybrids between Wem-1 type and *T. durum*; hybWEM_Dcs – hybrids between Wem-1 type and wild emmer; hybIrn_Dcs – hybrids between Iranian type and wild emmer; hybTrc_Eth – hybrid between Transcaucasian and Ethiopian types; hybTrc_Bal – hybrid between Transcaucasian and Bal types; hybTrc_Mor – hybrid between Trc and Mor types; hybTrc_Vol – hybrid between Trc and Vol types; hybTrc_WEM – hybrid between Trc and Wem-1 types; uncertain – karyotype of a line cannot be assigned to any known karyotypic groups.

Assignment in k-medoid analysis: the number of cluster is given according to Fig. 4; “-” – lines not included in analysis;

Collection site, donor, accession name – detailed location (if available), donor information, accession name (cultivar or designation of research material); information completed or corrected using the online systems Genesys (for ICARDA IG numbers), GBIS (for IPK TRI numbers), GRIN (for USDA-ARS PI numbers).
