# Supplementary material for: Chromosomal Passports Provide New Insights into Diffusion of Emmer Wheat
Source: PLoS One. 2015 May 29;10(5):e0128556. doi: 10.1371/journal.pone.0128556 (PMC4449015; doi:10.1371/journal.pone.0128556)
Supplement: S3 Table — Translocations described earlier. No.: 1chromosomal rearrangements described in [46]; 2chromosomal rearrangements described in [35], Translocation type: the superscripts correspond to the numbers of the respective translocations in a catalogue [46]. Structure of rearranged chromosomes—formed as a result of translocation/inversion; #: total number of lines carrying the respective translocation type; Geographical distribution: country in which the translocation type was identified (the number of lines carrying the respective translocation type is given in parenthesis). (DOCX) [file pone.0128556.s016.docx]

**S3 Table. List of chromosomal rearrangements identified in *Triticum dicoccon* and their geographical distribution*.*** Translocations described earlier.

| No. | Translocation type | Structure of rearranged chromosomes | # | Geographical distribution |
| --- | --- | --- | --- | --- |
|  | Normal karyotype | Normal | 334 | Afghanistan (3); Albania (1); Algeria (2); Armenia (10); Austria (1); Azerbaijan (2); Belgium (1); Bosnia and Herzegovina (10); Bulgaria (11); Belarus (3); China (2); Croatia (1); Czech Republic (4); former Czechoslovakia (5); Egypt (1); Ethiopia (39); France (8); Georgia (4); Germany (7); Great Britain (1); Greece (4); Hungary (3); India (10); Iran (19); Italy (2); Jordan (3); Kazakhstan (2); Latvia (3); Montenegro (9); Morocco (4); Oman (6); Palestine (7); Portugal (2); Romania (7); Russia (51); Saudi Arabia (2); Serbia (12); Slovakia (2); Spain (19); Sweden (1); Switzerland (1); Syria (2); Turkey (23); Ukraine (7); Uzbekistan (1); Yemen (11); former Yugoslavia (5) |
| 1^1^ | T7A:5B^4^ | T7AS.7AL-5BS + T5BL.5BS-7AL | 48 | Algeria (INRA 26893, INRA 26894); Belgium (IG 45423); Bulgaria (PI 295065); Belarus (k-18774b, k-39300-2); former Czechoslovakia (INRA 26651); France (INRA 898; INRA 6807; INRA 26647, INRA 26648, INRA 26657, INRA 26659); Germany (INRA 26639, INRA 27123, k-1730; k-18623, k-81); Hungary (INRA 26654, PI 290517); Iran (IG 127700=k-7146); Italy (INRA 27097, k-21416); Kuwait (TRI4342); Latvia (k-38185-6); Netherlands (k-35890); Poland (IG 45428= PI 286061); Romania (k-45926, PI 306536, PI 306537, PI 306538, PI 362071); Russia (IG 127709a, k-9934); Spain (INRA 27076, INRA 27094, k-20541, k-20368, PI 254193); Sweden (k-36527); Switzerland (INRA 23757, PI 355467); Turkey (PI 355454, TRI 584, k-20969); Ukraine (k-15007, k-19352-2, k-19360) |
| 2^1^ | T2A:6A^11^ | T2AS:6AL +T6AS:2AL | 2 | Ethiopia (INRA 27098, k-19285) |
| 3^1^ | T5A:6A^23^ | T5AS:6AS + T5AL:6AS | 1 | Ethiopia (k-24397) |
| 4^1^ | T4A:1B^38^ | T4AS:1BL + T1BS:4AL | 1 | Algeria (INRA 26896) |
| 5^1^ | T5A:1B^15^ | T1BS:5AL + T5AS:1BL | 1 | Ethiopia (IG 45123) |
| 6^1^ | T4B:6B-1 | T4BS:6BS + T4BL:6BL | 3 | Spain (IG 45398=PI 277671, PI 191091, PI 256031) |
| 7^1^ | T4B:6B-2^37^ | T4BS:6BL + T6BS:4BL | 2 | India (k-14928a, k-19475) |
| 8^1^ | T3B:4B-2^29^ | T3BS:4BL + T:4BS:3BL | 2 | Azerbaijan (k-30091); Russia (k-30728-3,6) |
| 9^1^ | T3B:6B-1^33^ | T3BS:6BS + T3BL:6BL | 5 | Belarus (k-18774a); Georgia (k-14934, TRI 16608); Ukraine (k-14999) |
| 10^1^ | T6B:7B-1^36^ | T6BS:7BL + T7BS:6BL | 3 | Bosnia and Herzegovina (IG 45377= PI 434998, k-38904, PI 434995t,) |
| 11^1^ | T5B:7B^6^ | T5BL.5BS-7BL + T7AS.7AL-5BS | 1 | Spain (k-308879) |
| 12^1^ | T4A:1B + T1A: 6A:4B: 6B^45,46,47^ | T4AS:1BL + T1BS:4AL + T6AL.6AS-1AL + T4BS:1AL-6AS + T4BL:6AL + T6BS:4BL | 3 | Morocco (IG 45317, IG 88730, k-22246) |
| 13^1^ | T4A:1B + T1A:6A + T4B:6B^45,46,47^ | T4AS:1BL + T1BS:4AL + T4BS:6BL + T6BS:4BL + T1AS.1AL-6AS + T6AL.6AS-1AL | 3 | Morocco (IG 127703, IG 127704, k-15837) |
| 14^1^ | Inv 3B^45^ | *per*Inv3B | 1 | Russia (k-22482) |
| 15^1^ | Inv5A^47^ | *par*Inv5AL | 3 | Spain (PI 276002, IG 45096); Iran (k-45543c) |
| 16^2^ | T7A:5B + T6A:1B | T7AS.7AL-5BS + T5BL.5BS-7AL + T6AS:1BL + T1BS:6AL | 1 | Germany (k-26642) |
| 17^2^ | Inv (T7A:5B) | *per*Inv(T5BL.5BS-7AL) + T7AS.7AL-5BS) | 2 | Switzerland (k-12946); Ukraine (k-19362) |

No.: ^1^chromosomal rearrangements described in [45];

^2^chromosomal rearrangements described in [34],

Translocation type: the superscripts correspond to the numbers of the respective translocations in a catalogue [45].

Structure of rearranged chromosomes: formed as a result of translocation/inversion;

#: total number of lines carrying the respective translocation type;

Geographical distribution: country in which the translocation type was identified (accession number of lines carrying the respective translocation is given in parenthesis).
