# Supplementary material for: Chromosomal Passports Provide New Insights into Diffusion of Emmer Wheat
Source: PLoS One. 2015 May 29;10(5):e0128556. doi: 10.1371/journal.pone.0128556 (PMC4449015; doi:10.1371/journal.pone.0128556)
Supplement: S4 Table — Novel translocations not included in the catalogue [46]. (DOCX) [file pone.0128556.s017.docx]

**S4 Table.** **List of chromosomal rearrangements identified in *Triticum dicoccon* and their geographical distribution*.*** Novel translocations not included in the catalogue [45].

| No. | Translocation type | Structure of rearranged chromosomes | # | Geographical distribution |
| --- | --- | --- | --- | --- |
| 18 | T2A:1B | T2AS.2SL-1BS+T1BL.1BS-2AL | 1 | Italy (INRA 27088) |
| 19 | T1A:4B + T4B:6B | T4AS:1BL + T1BS:4AL + T4BS:6BL + T6BS:4BL | 1 | Algeria (INRA 26897) |
| 20 | T1A:7B | T1AS:7BL + T7BS:1AL | 1 | Russia (PI 41025b) |
| 21 | T2A:4A | T2AS.2AL-4AS + T4AL.4AS-2AL | 1 | Russia (k-6246-1) |
| 22 | T2A:5B-3 | T2AS:5BS + T2AL:5BL | 1 | Ethiopia (INRA 27130) |
| 23 | T2B:4B | T2BS:4BS + T2BL:4BL | 1 | China (PI 79899) |
| 24 | T2B:7B | T2BS.2BL-7BS + T7BS.7BL-2BL | 6 | Russia (k-417-2, 6, 7, 10, 11, k-859) |
| 25 | T3A:4B | T3AL.3AS-4BL + T4BS.4BL-3AS | 3 | Germany (k-1777b); Italy (k-21310, k-21419) |
| 26 | T3B:4B-3 | T3BS.3BL-4BS + T4BL.4BS-3BL | 1 | Switzerland (PI 277129) |
| 27 | T4A:2B | T4AS.4AL-2BL + T2BS.2BL-4AL | 1 | Spain (PI 276013b) |
| 28 | T4A:4B-1 | T4BS.4BL-4AL + T4AS.4AL-4BL | 1 | Russia (k-30728-p) |
| 29 | T4A:4B-2 | T4AS:4BL + T4BS:4AL | 1 | Ukraine (k-19361) |
| 30 | T4A:5A | T4AS.4AL-5AS + T4AL-5AS.5AL | 2 | Ethiopia (IG 45124); Russia (k-64408) |
| 31 | T4A:6B | T4AS.4AL-6BS + T6BL.6BS-4AL | 1 | Russia (IG 45354=PI 2789) |
| 32 | T4A:7B | T4AS:7BS + T4AL:7BL | 1 | Spain (k-20579) |
| 33 | T4B:5B | T4BS:5BS + T4BL:5BL | 1 | Ukraine (PI 57536) |
| 34 | T4B:7B | T4BS:7BS + T4BL:7BL | 1 | Afghanistan (IG 45318b) |
| 35 | T6A:2B + T7A:3B | T2BS:6AL + T6AS:2BL +T3BS:7AL+ T7AS:3BL | 1 | Ethiopia (PI 577791) |
| 36 | T2A:4A + T6B:7B-2 | T2AS.2AL-4AS+T4AL.4AS-2AL+T6BS:7BS+T6BL:7BL | 1 | Russia (k-6246-5) |
| 37 | T3A:4B + T3B:6B-2 | T3AL.3AS-4BL + T4BS.4BL-3AS + T3BS:6BL +T6BS:3BL | 1 | Greece (IG 45410) |
| 38 | T7A:5B:5A + inv 4A | T5AS.5AL-7AS + T5AL-7AS.7AL-5BS + T5BL.5BS-7AL + perInv4A | 1 | Ukraine (k-15007-1) |
| 39 | T7A:5B + T2A:2B:6B | T7AS.7AL-5BS + T5BL.5BS-7AL + T2AS:6BS + T2BS:2AL + T2BL:6BL | 1 | Romania (k-45926-1) |
| 40 | T7A:5B + T3A:4A | T7AS.7AL-5BS + T5BL.5BS-7AL + T3AL.3AS-4AS + T3AS-4AS.4AL | 1 | Latvia (k-38185-1) |
| 41 | Inv5A-2 | *per*Inv5A | 1 | Turkey (TA 10480) |
| 42 | Inv3B-2 | *par*Inv3BS | 1 | Ethiopia (INRA 27141) |
| 43 | Inv2B | *per*Inv2B-1 | 2 | Russia (k-6249a (lines #3, 4, 5, 6, 9) |

Structure of rearranged chromosomes: formed as a result of translocation/inversion;

#: total number of lines carrying the respective translocation type;

Geographical distribution: country in which the translocation type was identified (accession number of lines carrying the respective translocation is given in parenthesis).
