# Supplementary material for: Chromosomal Passports Provide New Insights into Diffusion of Emmer Wheat
Source: PLoS One. 2015 May 29;10(5):e0128556. doi: 10.1371/journal.pone.0128556 (PMC4449015; doi:10.1371/journal.pone.0128556)
Supplement: S5 Table — (DOCX) [file pone.0128556.s018.docx]

| **S5 Table.** **Translocations identified in *T. dicoccoides*** | | | | |
| --- | --- | --- | --- | --- |
| No. | Translocation type | Structure of rearranged chromosomes | # | Geographical distribution |
| 1 | T1B:3B | T1BS:3BS + T1BL:3BL | 1 | Israel (UH-Gw-1) |
| 2 | T2B:4B-2 | T2BS.2BL-4BS + T4BL.4BS-2BL | 1 | Israel (UH-NM5) |
| 3 | T2B:5B-1 | T2BL.2BS-5BS + T5BL.5BS-2BS | 1 | Iraq (IG 109085) |
| 4 | T2B:5B-2 | T2BS:5BS + T2BL:5BL | 1 | Israel (TA 1057) |
| 5 | T2B:6B | T2BS:6BL + T6BS:2BL | 1 | Israel (UH-A3) |
| 6 | T3A:5A | T3AL.3AS-5AS + T5AL.5AS-3AS | 3 | Israel (UH-J4-1, J4-2, J5-1) |
| 7 | T3B:4B-2^29^ | T3BS:4BL + T:4BS:3BL | 2 | Lebanon (IG 46531b); Syria (IG 46472) |
| 8 | T3B:5B | T3BS:5BL + 5BS:T3BL | 1 | Lebanon (PI 470978) |
| 9 | T3B:6B | T3BS:6BS + T3BL:6BL | 1 | Turkey (PI 428063) |
| 10 | T4B:7B | T4BS:7BL + T7BS:4BL | 1 | Israel (IG 46306) |
| 11 | T5B:6B | T5BS:6BL + T6BS:5BL | 1 | Israel (PI 466978) |
| 12 | T7A:5B | T7AS.7AL-5BS + T5BL.5BS-7AL | 3 | Iraq (k-42642); Lebanon (PI 355455): Syria (k-17157) |
| 13 | T3B:5B:7B | T3BS:7BS + T5BS:3BL + T5BL:7BL | 1 | Israel (PI 428105) |
| 14 | T2A:7B + T4A:2B | T2AS:7BL + T7BS:2AL + T4AS:2BS + 4AL:T2BL | 1 | Israel (PI 538699) |
| 15 | T3A:5A + T5B:7B-2 | T3AL.3AS-5AS + T5AL.5AS-3AS + T5BS:7BL T7BS:5BL_ | 1 | Israel (TRI 17214) |
| 16 | T6A:5B | T6AS:5BL + T5BS:6AL | 1 | Syria (IG 139970) |
| 17 | T6B:7B | T6BS:7BL + T7BS:6BL | 1 | Jordan (IG 115808a) |
| 18 | Inv 2B | *per*Inv2B | 1 | Turkey (PI 596289) |

(DOC)
